# Variation in US Hospital Mortality Rates for Patients Admitted With COVID-19 During the First 6 Months of the Pandemic

**DOI:** 10.1001/jamainternmed.2020.8193

**Published:** 2020-12-22

**Authors:** David A. Asch, Natalie E. Sheils, Md Nazmul Islam, Yong Chen, Rachel M. Werner, John Buresh, Jalpa A. Doshi

**Affiliations:** 1 Division of General Internal Medicine, University of Pennsylvania, Philadelphia; 2 Leonard Davis Institute of Health Economics, University of Pennsylvania, Philadelphia; 3 UnitedHealth Group, Minnetonka, Minnesota; 4 Department of Biostatistics, Epidemiology, and Informatics, University of Pennsylvania, Philadelphia; 5 Cpl Michael J. Crescenz VA Medical Center, Philadelphia, Pennsylvania

## Abstract

**Importance:**

It is unknown how much the mortality of patients with coronavirus disease 2019 (COVID-19) depends on the hospital that cares for them, and whether COVID-19 hospital mortality rates are improving.

**Objective:**

To identify variation in COVID-19 mortality rates and how those rates have changed over the first months of the pandemic.

**Design, Setting, and Participants:**

This cohort study assessed 38 517 adults who were admitted with COVID-19 to 955 US hospitals from January 1, 2020, to June 30, 2020, and a subset of 27 801 adults (72.2%) who were admitted to 398 of these hospitals that treated at least 10 patients with COVID-19 during 2 periods (January 1 to April 30, 2020, and May 1 to June 30, 2020).

**Exposures:**

Hospital characteristics, including size, the number of intensive care unit beds, academic and profit status, hospital setting, and regional characteristics, including COVID-19 case burden.

**Main Outcomes and Measures:**

The primary outcome was the hospital’s risk-standardized event rate (RSER) of 30-day in-hospital mortality or referral to hospice adjusted for patient-level characteristics, including demographic data, comorbidities, community or nursing facility admission source, and time since January 1, 2020. We examined whether hospital characteristics were associated with RSERs or their change over time.

**Results:**

The mean (SD) age among participants (18 888 men [49.0%]) was 70.2 (15.5) years. The mean (SD) hospital-level RSER for the 955 hospitals was 11.8% (2.5%). The mean RSER in the worst-performing quintile of hospitals was 15.65% compared with 9.06% in the best-performing quintile (absolute difference, 6.59 percentage points; 95% CI, 6.38%-6.80%; *P* < .001). Mean RSERs in all but 1 of the 398 hospitals improved; 376 (94%) improved by at least 25%. The overall mean (SD) RSER declined from 16.6% (4.0%) to 9.3% (2.1%). The absolute difference in rates of mortality or referral to hospice between the worst- and best-performing quintiles of hospitals decreased from 10.54 percentage points (95% CI, 10.03%-11.05%; *P* < .001) to 5.59 percentage points (95% CI, 5.33%-5.86%; *P* < .001). Higher county-level COVID-19 case rates were associated with worse RSERs, and case rate declines were associated with improvement in RSERs.

**Conclusions and Relevance:**

Over the first months of the pandemic, COVID-19 mortality rates in this cohort of US hospitals declined. Hospitals did better when the prevalence of COVID-19 in their surrounding communities was lower.

## Key Points

**Question:**

Are hospital outcomes for patients with coronavirus disease 2019 (COVID-19) improving?

**Findings:**

In this cohort study of 38 517 adults who were admitted with COVID-19 to 955 US hospitals, rates of 30-day mortality or referral to hospice varied from 9.06% to 15.65% in the best- and worst-performing quintiles. In the early months of the pandemic, 94% of hospitals in a subset of 398 improved by at least 25%, and the strongest determinant of improvements in hospital-level outcome was a decline in community rates of infection.

**Meaning:**

All else being equal, COVID-19 mortality in hospitals seems to be lower when the prevalence of COVID-19 in their surrounding communities is lower.

## Teaser

This cohort study examines variations in COVID-19 mortality rates and over the first 6 months of the pandemic.

## Introduction

One year after identifying the first cases of coronavirus disease 2019 (COVID-19) in Wuhan, China, we have little understanding of how mortality rates vary by hospital or whether mortality rates are improving. Hospital-level mortality may depend not just on patient risk factors, but also on the hospital where patients are admitted. Individual-level and hospital-level mortality rates are also likely to improve over time with increasing experience with the disease and as new treatments become available. We used data from a large national health insurer in the US to estimate the variation in hospital-level mortality among patients hospitalized with COVID-19 to examine how mortality rates changed and identify hospital-level characteristics that were associated with those mortality rates and their change.

## Methods

### Data Sources

We used deidentified administrative claims data from a large national health insurer in the US that were linked with a daily record of patients who were admitted to a hospital with a primary or secondary diagnosis of COVID-19 (eTable 3 in the Supplement) and their current status (admitted, discharged, transferred, or dead) until June 30, 2020, within the UnitedHealth Group Clinical Discovery Database. Data specific to severe acute respiratory syndrome coronavirus 2 (SARS-CoV-2) and COVID-19 underwent an additional check for errors in sampling and data collection described in the eAppendix in the Supplement. We obtained hospital-level characteristics from the 2018 American Hospital Association Annual Survey,^1^ 2020 Medicare Impact,^2^ and 2020 Provider of Service files.^3^ We obtained cumulative COVID-19 case rates for January 1 to April 30, 2020, and May 1 to June 30, 2020 in the county of each hospital in our sample from *The New York Times* database.^4^ This study was reviewed and deemed exempt by the institutional review board of UnitedHealth Group.

### Patients and Hospitals

We started with all Medicare Advantage and commercial enrollees 18 years or older hospitalized with COVID-19 since January 1, 2020 (eFigure 1 in the Supplement). We then excluded patients with fewer than 6 months of insurance enrollment in 2019 (to permit comorbidity measurement using historical claims), were readmitted to or transferred to another facility within 30 days of initial admission (to prevent misattributing hospital-level outcomes among patients who were admitted to multiple hospitals), were admitted with COVID-19 after June 30, 2020 (to provide sufficient follow-up to measure 30-day event rates to July 31), and were admitted to hospitals with missing information or fewer than 10 patients (to improve the statistical reliability of our hospital-level mortality estimates). The hospitals where these patients were admitted constituted our main study sample.

### Outcome Measure

To reflect an outcome that was close to a 30-day any-site mortality rate, we measured a hospital’s risk standardized event rate (RSER), which reflected a composite of either inpatient mortality or referral to hospice within 30 days of initial admission for COVID-19 based on the National Quality Forum–approved hierarchical generalized linear models while accounting for clustering of patients within hospitals.^5^ We considered the composite of mortality or hospice referral as a more complete representation of the outcome of interest. We repeated all analyses using mortality alone (eFigures 2 and 3 in the Supplement).

### Statistical Analysis

The hierarchical models to estimate the RSER used the methods of George et al^6^ and Silber et al^7,8,9^ and are detailed in the eAppendix and eMethods in the Supplement. We modeled the log odds of patient events as a function of patient-level clinical and demographic variables, including age, sex, Elixhauser comorbidity indicators (eTable 3 in the Supplement),^10^ nursing facility admission source, and the number of days between January 1, 2020, and admission. The latter variable was chosen to account for likely improvements in patient outcomes as hospitals gained experience with COVID-19. As a sensitivity analysis, we also calculated the RSER for each hospital using the methods of Drye et al^11^ and Normand et al.^12^ The estimates from these measures were nearly identical (eFigure 9 in the Supplement). We computed each hospital’s RSER by calculating the average of the predicted risk of mortality or referral to hospice for all patients in the sample as if they had (hypothetically) been treated at this hospital.^10,11,12,13^ This approach uses a fixed patient population to fairly compare hospitals.

To examine changes in hospital RSERs, we repeated these analyses in the subset of hospitals with 10 or more patients admitted for COVID-19 in the early and later part of the observation period using a period indicator variable. We again used a fixed patient population: each hospital’s RSER was calculated as the average of the predicted risk of mortality or referral to hospice for all patients in the subsample as if they had (hypothetically) been treated at this hospital during this period. In overall, early-, and later-period analyses, we ordered hospitals into quintiles based on their RSERs. We used paired Wilcoxon sign rank tests to compare differences in RSERs across periods. We visualized each hospital’s change in RSER using a Bland-Altman plot.^13^

We estimated linear regressions to measure associations between hospital-level characteristics, including the number of beds, number of intensive care unit beds, profit status, academic hospital status, hospital setting (urban/nonurban), and a hospital’s (1) RSER in the sample of 955 hospitals and (2) difference in RSER between the early and late period in the sample of 398 hospitals. The regressions included the census region of the hospital location as a fixed effect and a measure of the COVID-19 case load in the hospital’s county, which was measured as the number of cumulative cases per 10 000 residents for January 1 to April 30, 2020, in the early period and May 1 to June 30, 2020, in the late period. They also include a binary indicator to note if cases increased during the late period.

All statistical tests were 2-sided, with a significance level of *P *> .05. All analyses were conducted using R, version 3.6.3 (R Foundation).^14^ All statistical code is included in the eMethods in the Supplement.

## Results

A total of 955 hospitals were included, reflecting 38 517 inpatients with COVID-19 who were admitted between January 1, 2020, and June 30, 2020 (Table 1) from 43 states and Washington DC. Overall, 3179 patients (8.25%) died, and 1433 patients (3.7%) were referred to hospice. The mean (SD) hospital-level risk-standardized rate of 30-day inpatient mortality or referral to hospice was 11.82% (2.50%) (eFigure 4 in the Supplement). In hierarchical models, several individual-level risk factors were strongly associated with the odds of 30-day inpatient mortality or referral to hospice. Men had odds 1.29 times higher than women (95% CI, 1.20-1.38; *P* < .001), patients older than 85 years had odds 14.52 times higher than those aged 18 to 45 years (95% CI, 10.75-19.61; *P* < .001), patients transferred from a nursing facility had odds 2.43 times higher than those admitted from the community (95% CI, 2.22-2.65; *P* < .001), patients with metastatic cancer had odds 1.85 times higher than those without (95% CI, 1.57-2.18; *P* < .001), and patients admitted within the first 90 days of 2020 had odds 2.58 times higher than those admitted 150 days or more into 2020 (95% CI, 2.29-2.90; *P* < .001) (eFigure 2A in the Supplement). Results that used mortality as the sole outcome were similar (eFigure 2B in the Supplement).

**Table 1.** Hospital and Patient Characteristics^a^

Characteristic	Total sample	Analysis of early and late periods		
		Both periods	Early period, January 1-April 30, 2020	Late period, May 1-June 30, 2020
Patients, No.	38 517	27 801	10 428	17 373
Hospitals, No.	955	398	398	398
Patient-level characteristics				
Age, mean (SD), y	70.2 (15.5)	70.9 (15.2)	70.9 (15.1)	70.9 (15.3)
Age range, y				
18-45	2802 (7.3)	1855 (6.7)	691 (6.6)	1164 (6.7)
45-55	2891 (7.5)	1912 (6.9)	735 (7.0)	1177 (6.8)
55-65	5867 (15.2)	3916 (14.1)	1453 (13.9)	2463 (14.2)
65-75	10 283 (26.7)	7621 (27.4)	2903 (27.8)	4718 (27.2)
75-85	9863 (25.6)	7445 (26.8)	2776 (26.6)	4669 (26.9)
≥85	6811 (17.7)	5052 (18.2)	1870 (17.9)	3182 (18.3)
Male sex	18 888 (49.0)	13 552 (48.7)	5230 (50.2)	8322 (47.9)
Mean Elixhauser score (SD)	8.5 (11.0)	8.8 (11.2)	8.9 (11.3)	8.7 (11.1)
Elixhauser comorbidities				
Iron deficiency anemia	12 420 (32.2)	9266 (33.3)	3614 (34.7)	5652 (32.5)
Blood loss anemia	1574 (4.1)	1159 (4.2)	428 (4.1)	731 (4.2)
Congestive heart failure	9663 (25.1)	7171 (25.8)	2708 (26.0)	4463 (25.7)
Chronic obstructive pulmonary disease	12 654 (32.9)	9117 (32.8)	3467 (33.2)	5650 (32.5)
Coagulopathy	2786 (7.2)	2119 (7.6)	784 (7.5)	1335 (7.7)
Depression	8188 (21.3)	5876 (21.1)	2194 (21.0)	3682 (21.2)
Diabetes without chronic complications	14 139 (36.7)	10 275 (37.0)	3987 (38.2)	6288 (36.2)
Diabetes with chronic complications	12 148 (31.5)	8857 (31.9)	3426 (32.9)	5431 (31.3)
Substance use disorder	1437 (3.7)	1036 (3.7)	379 (3.6)	657 (3.8)
Hypertension	29 335 (76.2)	21 413 (77.0)	8004 (76.8)	13 409 (77.2)
Hypothyroidism	8096 (21.0)	5900 (21.2)	2305 (22.1)	3595 (20.7)
Lymphoma	734 (1.9)	591 (2.1)	229 (2.2)	362 (2.1)
Fluid and electrolyte disorder	10 238 (26.6)	7541 (27.1)	2811 (27.0)	4730 (27.2)
Metastatic cancer	1456 (3.8)	1092 (3.9)	396 (3.8)	696 (4.0)
Neurological disorder	9048 (23.5)	6615 (23.8)	2555 (24.5)	4060 (23.4)
Obesity	10 100 (26.2)	7156 (25.7)	2702 (25.9)	4454 (25.6)
Paralysis	1938 (5.0)	1396 (5.0)	570 (5.5)	826 (4.8)
Peripheral vascular disease	9899 (25.7)	7430 (26.7)	2952 (28.3)	4478 (25.8)
Psychosis	2578 (6.7)	1887 (6.8)	766 (7.3)	1121 (6.5)
Chronic kidney disease	9832 (25.5)	7346 (26.4)	2760 (26.5)	4586 (26.4)
Solid tumor without metastasis	5352 (13.9)	4042 (14.5)	1455 (14.0)	2587 (14.9)
Valvular disorder	7723 (20.1)	5778 (20.8)	2099 (20.1)	3679 (21.2)
Weight loss	3528 (9.2)	2661 (9.6)	1036 (9.9)	1625 (9.4)
Transferred from a nursing facility	4244 (11.0)	3034 (10.9)	1690 (16.2)	1344 (7.7)
Insurance type				
Medicare Advantage	29 081 (75.5)	21 716 (78.1)	8047 (77.2)	13 669 (78.7)
Commercial	9436 (24.5)	6085 (21.9)	2381 (22.8)	3704 (21.3)
Hospital-level characteristics				
Hospital size				
0-150 Beds	222 (23.2)	63 (15.8)	NA	NA
150-300 Beds	336 (35.2)	121 (30.4)		
300-450 Beds	194 (20.3)	88 (22.1)		
≥450 Beds	203 (21.3)	126 (31.7)		
No. of ICU beds				
0-20	319 (33.4)	107 (26.9)	NA	NA
20-60	346 (36.2)	135 (33.9)		
≥60	290 (30.4)	156 (39.2)		
Hospital setting				
Urban	892 (93.4)	385 (96.7)	NA	NA
Nonurban	63 (6.6)	13 (3.3)		
Hospital region				
Northeast	264 (27.6)	126 (31.7)	NA	NA
South	285 (29.8)	133 (33.4)		
Midwest	315 (33.0)	113 (28.4)		
West	91 (9.5)	26 (6.5)		
Profit status				
Nonprofit	705 (73.8)	307 (77.1)	NA	NA
For profit	139 (14.6)	46 (11.6)		
Other	111 (11.6)	45 (11.3)		
Academic hospital	154 (16.1)	94 (23.6)	NA	NA

Abbreviations: ICU, intensive care unit; NA, not applicable.

^a^ Unless otherwise indicated, data are reported as number (percentage) of patients.

**Figure 1.**
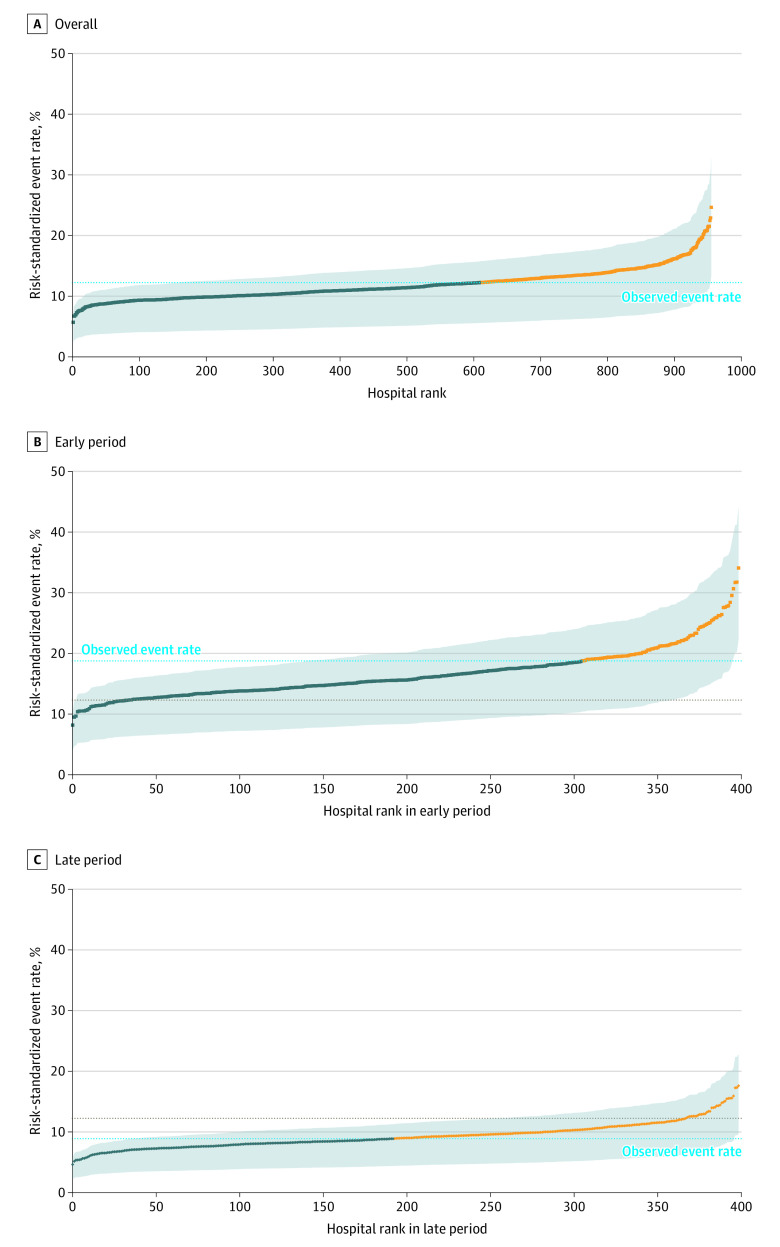
Hospital-Specific Risk-Standardized Event Rates for 30-Day Mortality or Referral to Hospice A, Risk-standardized event rates for all 955 hospitals and 38 517 patients. B, Risk-standardized event rates for 398 of these hospitals with patients admitted during the early period (January 1 to April 30, 2020 [gray dotted line indicates observed event rate during this period]). C, Substantially lower risk-standardized event rates for the same 398 hospitals (not necessarily in the same order) with patients admitted in the late period (May 1 to June 30, 2020 [gray dotted line indicates observed event rate during this period]). The blue dashed line representing the overall 2-period observed risk rate is the same in B and C to facilitate comparison. The dark blue dots represent the hospitals with a risk-standardized event rate below the overall observed rate, and the yellow dots represent those above. The gray shaded area indicates the interquartile range for the risk-standardized event estimate. A numerically higher rank corresponds to worse performance.

The RSERs varied considerably across hospitals, ranging from 5.70% to 24.65% (Figure 1). The mean RSER in the worst-performing quintile of hospitals was 15.65% compared with 9.06% in the best-performing quintile of hospitals (absolute difference, 6.59 percentage points; 95% CI, 6.38%-6.80%; *P* < .001) (Table 2; eTable 1 in the Supplement). Variation was substantial regardless of the county-level cumulative COVID-19 case burden (eTable 2 in the Supplement). Hospital ranks based on the composite of mortality or hospice referral and on mortality alone were statistically significantly correlated (Kendall rank correlation coefficient, 0.628; *P* < .001) (eFigure 3 in the Supplement).

**Table 2.** Risk Standardized 30-Day Mortality or Referral to Hospice Rates or Risk-Standardized 30-Day Mortality Rates Overall and During the Early and Late Periods

Quintile	RSER (95% CI)		
	Overall (N = 955)	Early period (n = 398)	Late period (n = 398)
**Mortality or referral to hospice**			
Q1	9.06 (8.96-9.16)	12.19 (11.97-12.42)	6.88 (6.73-7.03)
Q2	10.28 (10.24-10.33)	14.13 (14.04-14.23)	8.11 (8.06-8.17)
Q3	11.36 (11.31-11.41)	15.78 (15.66-15.90)	8.99 (8.92-9.05)
Q4	12.74 (12.68-12.81)	17.95 (17.80-18.10)	9.99 (9.92-10.07)
Q5	15.65 (15.34-15.96)	22.73 (21.99-23.48)	12.47 (12.10-12.84)
**Quintile**	**RSMR (95% CI)**		
	**Overall (N = 955)**	**Early period (n = 398)**	**Late period (n = 398)**
**Mortality alone**			
Q1	5.17 (5.09-5.25)	7.26 (7.07-7.44)	3.32 (3.23-3.41)
Q2	6.12 (6.09-6.16)	9.20 (9.10-9.30)	4.20 (4.15-4.25)
Q3	7.08 (7.04-7.12)	10.85 (10.74-10.97)	5.12 (5.06-5.18)
Q4	8.43 (8.35-8.50)	13.10 (12.93-13.26)	6.13 (6.06-6.21)
Q5	11.88 (11.51-12.24)	18.61 (17.85-19.36)	8.69 (8.32-9.05)

Abbreviations: Q, quintile; RSER, risk-standardized event rate; RSMR, risk-standardized 30-day mortality rates.

**Figure 2.**
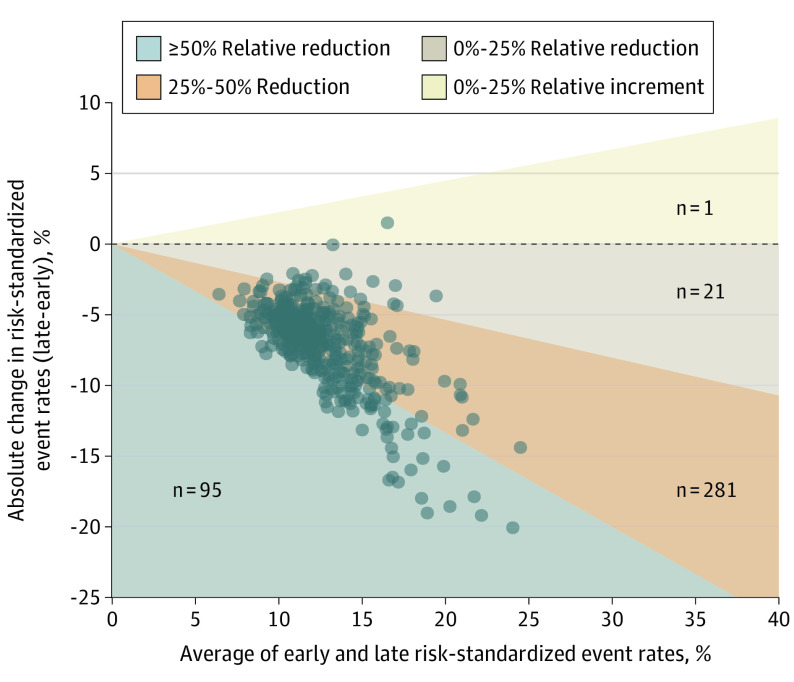
Two-Period Change in Hospital-Level Risk-Standardized Event Rates Between the Early and Late Periods Of 398 hospitals with at least 10 inpatients admitted with coronavirus disease 2019 (COVID-19) during each period, 397 improved their scores from the early period to the late period, shown by vertical distance below the horizontal dashed line in the Bland-Altman plot. The 95 hospitals in the grey region revealed at least a 50% reduction in event rate. The 281 hospitals in the orange region revealed a 25% to 50% reduction in event rate. The 21 hospitals in the beige region revealed a reduction of less than 25% in event rates. The single hospital in the yellow region revealed only a small increase in score. The general sloping of the scatter downward to the right suggests that hospitals with worse overall scores tended to show the most improvement.

A total of 398 hospitals (41.7%) had sufficient patient volume during the early (admitted January 1, 2020, to April 30, 2020) and late (admitted May 1, 2020, to June 30, 2020) periods to compare mortality rates. The 27 801 patients (72.2%) in this subset had characteristics similar to the overall set of patients, although hospitals were slightly more likely to be larger and academic (Table 1). The overall mean (SD) RSER declined from 16.56% (3.99%) to 9.29% (2.08%) (Figure 1; eFigures 5, 6, and 7 in the Supplement). The mortality rate of all hospitals but 1 improved: 281 hospitals (70.6%) revealed a 25% to 50% reduction in event rates, and 95 hospitals (24.0%) revealed more than a 50% reduction in event rates (Figure 2). The individual positions of hospitals in the rankings changed between periods but were correlated (Kendall rank correlation, 0.4731; *P* < .001), suggesting that better-performing hospitals continued to be better performers (eFigure 10 in the Supplement). From the early to late period, the absolute difference in the rates of mortality or referral to hospice between the worst- and best-performing quintiles of hospitals decreased from 10.54 percentage points (95% CI, 10.03%-11.05%; *P* < .001) to 5.59 percentage points (95% CI, 5.33%-5.86%; *P* < .001) (eTable 1 in the Supplement). Even in the later period, the RSER in the worst-performing quintile of hospitals was 12.47% compared with 6.88% in the best-performing quintile of hospitals (Table 2).

**Figure 3.**
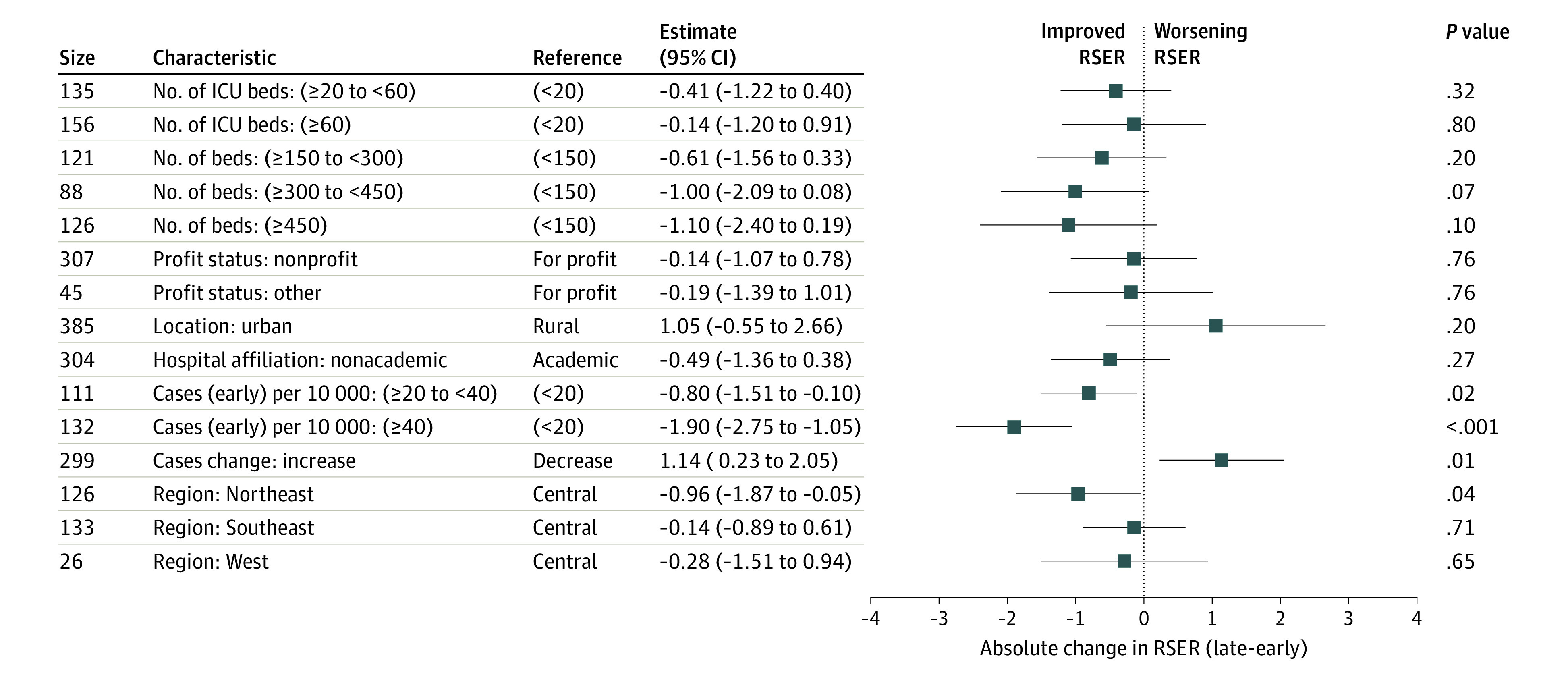
Hospital Characteristics Associated With Change in Risk-Standardized Event Rates Between the Early and Late Periods in 398 Hospitals Negative change in risk-standardized event rates from the late period to the early period (shown to the left of the dotted line) reflect characteristics associated with an improvement in hospital risk-standardized event rates. Higher early period community coronavirus disease 2019 (COVID-19) case rates were associated with decreases in late period risk-standardized event rates, and increases in community COVID-19 case rates were associated with increases in late period risk-standardized event rates, adjusting for other factors. ICU indicates intensive care unit.

We found no association between number of intensive care unit beds, academic status, profit status, or urban/nonurban setting and a hospital’s RSER (eFigure 8 in the Supplement) except that medium to large hospitals, hospitals in the Northeast, and hospitals with high county-level COVID-19 case rates had worse RSERs. These results were largely sustained in a hierarchical model, which simultaneously reflected patient and hospital attributes (eFigure 11 in the Supplement). The characteristic that was associated with the largest change in a hospital’s RSER over the 2 periods was the COVID-19 burden in the community; higher early-period community case rates were associated with improvements in RSER, and increases in community case rates were associated with worsening RSER after adjusting for other factors (Figure 3).

## Discussion

This study had 4 main findings. First, mortality rates for patients with COVID-19 varied significantly across US hospitals. Second, RSERs in all but 1 hospital improved over the first 6 months of the pandemic. Third, while absolute mortality differences across hospitals declined, wide differences persisted. Fourth, the characteristic most associated with RSER and its change was the community case rate of COVID-19; high county-level case rates were associated with worse RSERs and with improvements in RSERs over time. Increases in county-level case rates were associated with worsening RSERs.

This study extended past findings of variation in outcomes and improvement over time.^15,16^ It also confirmed individual-level risk factors that were previously identified, including advanced age, male sex, medical comorbidities, and nursing facility sources.

A central finding of this study was that 94% of hospitals had a relative reduction in COVID-related mortality rates of more than 25% in just a few months. That rate of relative improvement is striking and encouraging, but perhaps not surprising. Early efforts at treating patients with COVID-19 were based on experience with previously known causes of severe respiratory illness. Later efforts could draw on experiences specific to SARS-CoV-2 infection. Remdesivir received a US Food and Drug Administration emergency use approval on May 1, 2020,^17^ the start of the study’s later period, although, to our knowledge, a survival benefit has not been shown.^18^ A June 16, 2020, statement from the RECOVERY investigators reported a substantial survival benefit from dexamethasone in selected patients with respiratory failure.^19,20^ Considerable changes in inpatient management were tried (eg, early vs late assisted ventilation, differences in oxygen flow, prone or supine positioning, and anticoagulation). Those efforts varied in how systematically they were evaluated, but our results suggest that valuable experience was gained. Another possible reason for improvement includes greater use of masks which, theoretically, could reduce the viral inoculum and perhaps disease severity.^21^ In general, health outcomes improve with time, but this novel viral infection and our early access to a large set of patients provided an opportunity to see rapid improvement.

Despite these widespread improvements for nearly all hospitals, this study also revealed large differences in mortality or referral to hospice between the best- and worst-performing hospitals. Decades of quality measurement often reveal differences in outcomes across hospitals.^22,23,24^ The large differences observed in this study could reflect large differences in fundamental quality, but they could also reflect different admission thresholds across hospitals. For example, although we could adjust for differences in medical comorbidities, COVID-19 presents heterogeneously, and we could not adjust for differences in the manifestations of COVID-19 itself.

### Strengths and Limitations

This study has additional limitations. First, the calculated event rates reflect patients from a single insurer and, therefore, also a limited set of hospitals. Nevertheless, this study reflects what is to our knowledge the largest and most comprehensive sample of US patients with COVID-19 to date, covering commercially and Medicare-insured populations. Second, we are unable to measure out-of-hospital mortality. However, most COVID-19 mortality among inpatients occurs in the hospital and so should be observable in our data. We used the composite outcome of death or referral to hospice within 30 days to reflect any-site mortality more comprehensively. That composite reflects what is likely a more complete assessment of the outcome of interest than used in studies that are restricted to inpatient mortality. Nevertheless, the fundamental findings of this study were preserved in sensitivity analyses that used mortality alone as the outcome. Third, we did not measure morbidity and disability outcomes among survivors that may be meaningful. Fourth, to measure county-level disease burden, we used cumulative reported case rates that could be sensitive to varied testing availability and use. However, these values were highly correlated with death rates 1 month later, which would be less sensitive to testing availability or use (correlation coefficient, 0.881; *P* < .001). Fifth, in using insurance claims-based information, we were unable to examine processes of care that may also help to explain variation in outcomes.

This study also has strengths. It represents a geographically and sociodemographically diverse group of 38 517 patients and 955 hospitals, allowing confidence in the estimation of individual-level patient factors associated with mortality, and variation in hospital mortality rates and their changes.

## Conclusions

This study revealed that outcomes for patients with COVID-19 rely not only on individual-level risk factors, but also on the hospital where care is received. This study also revealed that during the first 6 months of the COVID-19 pandemic, mortality rates in US hospitals declined sharply. Nevertheless, the characteristic that is most associated with poor or worsening hospital outcomes is high or increasing community case rates. The association between high community COVID-19 case loads and both worse RSERs and greater improvement in RSERs suggests hospitals do worse when they are burdened with cases and is consistent with imperatives to flatten the curve. As case rates of COVID-19 increase across the nation, hospital mortality outcomes may worsen.

## Notes

Supplement. eAppendix. **eFigure 1.** Derivation and geographic distribution of the study sample **eFigure 2.** Estimated odds ratios of 30-day mortality **eFigure 3.** Correlation of hospital rank based on the composite of mortality of hospice referral or mortality alone **eFigure 4.** Distribution of risk standardized event rates for 955 hospitals and 38,517 patients **eFigure 5.** Distribution of risk standardized event rates for 398 hospitals in the early period **eFigure 6.** Distribution of risk standardized event rates for 398 hospitals in the later period **eFigure 7.** Comparison of early and late period hospital-specific risk standardized rates for 30-day mortality or referral to hospice for 398 hospitals and 27,801 patients. A higher score corresponds to worse performance **eFigure 8.** Hospital characteristics associated with the risk standardized event rates of 955 hospitals **eFigure 9.** Correlation of the direct standardized event rate used in this analysis and the indirect standardized event rate **eFigure 10.** Correlation of the ranking for hospitals in the early and later periods **eFigure 11.** A hierarchical model reflecting both patient-and-hospital attributes. This analysis reveals how much variation in patient-level mortality can be explained by hospital attributes **eTable 1. **Difference in 30-day mortality **eTable 2. **Risk standardized 30-day mortality or referral to hospice rates stratified by county-level cumulative reported COVID-19 case burden as of April 30, 2020 (955 hospitals) **eTable 3. **ICD-10 codes used in the analyses **eMethods.**

## References

[1] American Hospital Association. AHA annual survey database. Accessed July 29, 2020. https://www.ahadata.com/aha-annual-survey-database

[2] US Centers for Medicare & Medicaid Services. FY 2020 final rule and correction notice data files. Accessed June 29, 2020. https://www.cms.gov/Medicare/Medicare-Fee-for-Service-Payment/AcuteInpatientPPS/FY2020-IPPS-Final-Rule-Home-Page-Items/FY2020-IPPS-Final-Rule-Data-Files

[3] US Centers for Medicare & Medicaid Services. 2019 POS file. Accessed September 4, 2020. https://www.cms.gov/research-statistics-data-systems/provider-services-current-files/2019-pos-file

[4] New York Times. Date, county, state, fips, cases, deaths. Accessed November 1, 2020. https://raw.githubusercontent.com/nytimes/covid-19-data/master/us-counties.csv

[5] National Quality Forum. Measure evaluation criteria and guidance for evaluating measures for endorsement. Accessed September 1, 2019. http://www.qualityforum.org/docs/measure_evaluation_criterias.aspx

[6] George EI, Ročková V, Rosenbaum PR, Satopää VA, Silber JH. Mortality rate estimation and standardization for public reporting: Medicare’s Hospital Compare. J Am Stat Assoc. 2017;112:519, 933-947. doi:10.1080/01621459.2016.1276021

[7] Silber JH, Rosenbaum PR, Brachet TJ, . The Hospital Compare mortality model and the volume-outcome relationship. Health Serv Res. 2010;45(5 Pt 1):1148-1167. doi:10.1111/j.1475-6773.2010.01130.x 20579125 PMC2965498

[8] Silber JH, Satopää VA, Mukherjee N, . Improving Medicare’s Hospital Compare Mortality Model. Health Serv Res. 2016;51(suppl 2):1229-1247. doi:10.1111/1475-6773.12478 26987446 PMC4874942

[9] Silber JH, Rosenbaum PR, Niknam BA, . Comparing outcomes and costs of surgical patients treated at major teaching and nonteaching hospitals: a national matched analysis. Ann Surg. 2020;271(3):412-421. doi:10.1097/SLA.0000000000003602 31639108

[10] Elixhauser A, Steiner C, Harris DR, Coffey RM. Comorbidity measures for use with administrative data. Med Care. 1998;36(1):8-27. doi:10.1097/00005650-199801000-00004 9431328

[11] Drye EE, Normand SL, Wang Y, . Comparison of hospital risk-standardized mortality rates calculated by using in-hospital and 30-day models: an observational study with implications for hospital profiling. Ann Intern Med. 2012;156(1 Pt 1):19-26. doi:10.7326/0003-4819-156-1-201201030-00004 22213491 PMC3319769

[12] Normand S-LT, Shahian DM. Statistical and clinical aspects of hospital outcomes profiling. Stat Sci. 2007;22:206-226. doi:10.1214/088342307000000096

[13] Bland JM, Altman DG. Statistical methods for assessing agreement between two methods of clinical measurement. Lancet. 1986;1(8476):307-310. doi:10.1016/S0140-6736(86)90837-8 2868172

[14] R Core Team. R: a language and environment for statistical computing. Accessed December 12, 2020. http://www.R-project.org/.

[15] Gupta S, Hayek SS, Wang W, ; STOP-COVID Investigators. Factors associated with death in critically ill patients with coronavirus disease 2019 in the US. JAMA Intern Med. 2020. doi:10.1001/jamainternmed.2020.3596 32667668 PMC7364338

[16] Horwitz LI, Jones SA, Cerfolio RJ, . Trends in COVID-19 risk-adjusted mortality rates. J Hosp Med. 2020. Published online October 23, 2020. doi:10.12788/jhm.3552 33147129

[17] US Food and Drug Administration. Veklury (remdesivir) EUA letter of approval. Accessed December 12, 2020. https://www.fda.gov/media/137564/download

[18] Spinner CD, Gottlieb RL, Criner GJ, ; GS-US-540-5774 Investigators. Effect of remdesivir vs standard care on clinical status at 11 days in patients with moderate COVID-19: a randomized clinical trial. JAMA. 2020;324(11):1048-1057. doi:10.1001/jama.2020.16349 32821939 PMC7442954

[19] https://www.ox.ac.uk/news/2020-06-16-low-cost-dexamethasone-reduces-death-one-third-hospitalised-patients-severe.

[20] Horby P, Lim WS, Emberson JR, ; RECOVERY Collaborative Group. Dexamethasone in hospitalized patients with covid-19—preliminary report. N Engl J Med. 2020. doi:10.1101/2020.06.22.20137273 32678530 PMC7383595

[21] Gandhi M, Rutherford GW. Facial masking for covid-19—potential for “variolation” as we await a vaccine. N Engl J Med. 2020;383(18):e101. doi:10.1056/NEJMp2026913 32897661 PMC7890559

[22] Chassin MR, Park RE, Lohr KN, Keesey J, Brook RH. Differences among hospitals in Medicare patient mortality. Health Serv Res. 1989;24(1):1-31. 2654083 PMC1065550

[23] Jha AK, Li Z, Orav EJ, Epstein AM. Care in US hospitals —the Hospital Quality Alliance program. N Engl J Med. 2005;353(3):265-274. doi:10.1056/NEJMsa051249 16034012

[24] Tsai TC, Joynt KE, Orav EJ, Gawande AA, Jha AK. Variation in surgical-readmission rates and quality of hospital care. N Engl J Med. 2013;369(12):1134-1142. doi:10.1056/NEJMsa1303118 24047062 PMC4107655

