# Supplementary material for: Variation in US Hospital Mortality Rates for Patients Admitted With COVID-19 During the First 6 Months of the Pandemic
Source: JAMA Intern Med. 2020 Dec 22;181(4):471–8. doi: 10.1001/jamainternmed.2020.8193 (PMC7756246; doi:10.1001/jamainternmed.2020.8193)
Supplement: Supplement. — eAppendix. eFigure 1. Derivation and geographic distribution of the study sample eFigure 2. Estimated odds ratios of 30-day mortality eFigure 3. Correlation of hospital rank based on the composite of mortality of hospice referral or mortality alone eFigure 4. Distribution of risk standardized event rates for 955 hospitals and 38,517 patients eFigure 5. Distribution of risk standardized event rates for 398 hospitals in the early period eFigure 6. Distribution of risk standardized event rates for 398 hospitals in the later period eFigure 7. Comparison of early and late period hospital-specific risk standardized rates for 30-day mortality or referral to hospice for 398 hospitals and 27,801 patients. A higher score corresponds to worse performance eFigure 8. Hospital characteristics associated with the risk standardized event rates of 955 hospitals eFigure 9. Correlation of the direct standardized event rate used in this analysis and the indirect standardized event rate eFigure 10. Correlation of the ranking for hospitals in the early and later periods eFigure 11. A hierarchical model reflecting both patient-and-hospital attributes. This analysis reveals how much variation in patient-level mortality can be explained by hospital attributes eTable 1. Difference in 30-day mortality eTable 2. Risk standardized 30-day mortality or referral to hospice rates stratified by county-level cumulative reported COVID-19 case burden as of April 30, 2020 (955 hospitals) eTable 3. ICD-10 codes used in the analyses eMethods. [file jamainternmed-e208193-s001.pdf]

Supplementary Online Content

Asch DA, Sheils NE, Islam MN, et al. Variation in US hospital mortality rates for patients admitted with COVID-19 during the first 6 months of the pandemic. JAMA Intern Med. Published Online December 22, 2020 doi:10.1001/jamainternmed.2020.8193

**eAppendix.**
**eFigure 1.** Derivation and geographic distribution of the study sampleeFigure 2. Estimated odds ratios of 30-day mortality
**eFigure 2**. Estimated odds ratios of 30-day
**eFigure 3.** Correlation of hospital rank based on the composite of mortality of hospice referral or mortality alone
**eFigure 4.** Distribution of risk standardized event rates for 955 hospitals and 38,517 patients
**eFigure 5.** Distribution of risk standardized event rates for 398 hospitals in the early period
**eFigure 6.** Distribution of risk standardized event rates for 398 hospitals in the later period
**eFigure 7.** Comparison of early and late period hospital-specific risk standardized rates for 30-day mortality or referral to hospice for 398 hospitals and 27,801 patients. A higher score corresponds to worse performance
**eFigure 8.** Hospital characteristics associated with the risk standardized event rates of 955 hospitals
**eFigure 9.** Correlation of the direct standardized event rate used in this analysis and the indirect standardized event rate
**eFigure 10.** Correlation of the ranking for hospitals in the early and later periods
**eFigure 11.** A hierarchical model reflecting both patient-and-hospital attributes. This analysis reveals how much variation in patient-level mortality can be explained by hospital attributes
**eTable 1.** Difference in 30-day mortality
**eTable 2.** Risk standardized 30-day mortality or referral to hospice rates stratified by county-level cumulative reported COVID-19 case burden as of April 30, 2020 (955 hospitals)
**eTable 3.** ICD-10 codes used in the analyses
**eMethods.**

This supplemental material has been provided by the authors to give readers additional information about their work.

© 2020 Asch DA et al. JAMA Internal Medicine.

## Data Sources

We used administrative claims for Medicare Advantage and commercially insured enrollees in a research database from a single large health insurance provider in the United States.

We also used an inpatient COVID-19 dataset which included a daily updated record of COVID-19 inpatient admissions for all insurance enrollees with claims information, representing those admitted to a hospital with a primary or secondary diagnosis of COVID-19 (eTable 3), along with their current disposition (admitted, discharged, transferred, or expired).

## Standardization of data entry and data structure

Medical and pharmacy claims data are captured, predominantly electronically, from sites of care seeking third-party reimbursement for both Medicare and commercial plans using the industry standard data collection forms HCFA/CMS-1500 for facility claims, UB04/CMS-1450 for professional services and outpatient claims, and NCPDP for pharmacy claims or their electronic equivalents. Structured data from these standardized forms are coded using the International Classification of Diseases, Tenth Revision, Clinical Modification (ICD-10-CM), National Drug Codes (NDC), Current Procedural Terminology (CPT) codes, and Logical Observation Identifiers Names and Codes (LOINC) codes, and Diagnosis Related Groups (DRG). This nomenclature ensures consistency of data collection across geographic regions, health systems, and payers throughout the United States.

## Methods to Control for Errors in Sampling and Data Collection

Claims that do not adhere to the form or coding standards described above are rejected from reimbursement, minimizing the risk that inappropriately structured data are included in the database. Data specific to SARS-CoV-2 and COVID-19 has an additional Quality Control layer to control for errors in sampling and data collection; this is described below in the section on Quality Control.

## Quality control

A COVID-19 data source-specific layer of quality control is also present, given the rapidly evolving situation. SARS-CoV-2 lab tests included in the database exclude custom local codes or codes that are not present in the LOINC organization's guidance for mapping SARS-CoV-2 and COVID-19 related LOINC terms. Test information provided via the LOINC code compliments the test type (antibody, RT-PCR, etc.) as well as the result value (detected, not detected, not given/cancelled).

## Differences across groups

While the data for Medicare Advantage and commercially insured enrollees is processed in a similar manner, these groups are substantially different. First, there are systematic differences in patient characteristics, most remarkably the older age and the higher prevalence of all comorbidities. These differences are tabulated in the database. Furthermore, the commercial insurance claims that are available for analyses are a subset of the overall commercially insured population.

## Data Sharing

The data are proprietary and are not available for public use but, under certain conditions, may be made available to editors and their approved auditors under a data use agreement to confirm the findings of the current study.

## Technical details for Statistical Analyses

Let $i$ denote the $i^{\text{th}}$ hospital and $j$ denote the $j^{\text{th}}$ COVID patient admitted in the $i^{\text{th}}$ hospital with $i = 1, \ldots, I$ and $j = 1, \ldots, n_i$. Let $Y_{ij}$ refer to the status of the $j^{\text{th}}$ patient which takes values either 1 if a patient is deceased or referred to hospice within 30-day from hospital admission date, and 0 if neither

© 2020 Asch DA et al. JAMA Internal Medicine.

death nor discharge to hospice happens within 30-day from hospital admission date. Denote by $X_{ijk}$ the $k^{\text{th}}$ patient-specific characteristics (e.g., gender, age, status of transfer from a nursing facility and risk factors such as indication of type-II diabetes, obesity, kidney disease, chronic obstructive pulmonary disease, heart failure, coronary artery disease, cardiomyopathy, and others that are used in calculating Elixhauser comorbidity index, see eTable 3 for the complete list of variables). In addition, we consider variables quantifying days since January 1, 2020 until a patient's admission dates to characterize hospital's effort to adapt to the changing conditions of the pandemic.

## Variable selection

To screen influential variables and avoid overfitting, we fit a series of univariate generalized linear models (GLM) with a logit link function for each covariate of interest. We classify age and counts of days into discrete categories for ease of interpretability and numerical stability. We repeat the process for each covariate, test whether the corresponding effect is zero, and select the ones that are less than a level of significance threshold (i.e. 10%) based on p-value calculated from Wald-Z statistic. Comorbidity variables of alcohol abuse, liver disease, peptic ulcer, rheumatoid arthritis, and pulmonary circulation disorder are excluded as their corresponding p-values are greater than 0.10; HIV/AIDS are also excluded due to having too few cases (prevalence of exposure being less than 1%). We retain the variable referring to the number of patients admitted to a hospital in our subsequent analyses irrespective to its statistical insignificance based on univariate analysis. We use the selected covariates to conduct the subsequent regression analyses.

## Calculation of hospital-specific risk standardized event rates

Hospital specific standardized risk scores are estimated using a hierarchical generalized linear model, following the methods of George, et al.[1] and Silber, et al.[2,3,4] To account for association between hospital adverse event rates and volume of patients being treated in each hospital after adjusting for other risk factors, we add a hospital attribute (i.e., logarithm of volume) in the model. Specifically, we cast the model into a generalized linear mixed model (GLMM) framework -

$$logit\ \Pr(Y_{ij} = 1) = \beta_0 + \sum_{k=1}^{K} X_{ijk}\,\beta_k \ + \gamma log(vol_i) + \ b_{i0};$$

where fixed effect parameters, denoted by $\theta = \ (\beta_0, \beta_1, \dots, \beta_K, \gamma)$, characterize the effects of covariates on likelihood of experiencing the events and quantify the deviation from overall mean $\beta_0$. In addition, $b_{i0}$ follows a Gaussian distribution with mean 0 and unknown variance $\sigma^2_{0b}$. Here the site-specific random effect quantifies deviation from the population mean, $\mu = \beta_0 + \sum_{k=1}^{K} X_{ijk}\,\beta_k + \gamma log(vol_i)$, and characterizes where a particular site "*sits*" in the population. This model can be viewed as a hierarchical model where at the first level, we adjust for patient-specific demographic, comorbidities, and hospital-specific intercept; and in the second level, hospital-specific intercept is further modeled through an overall mean, fixed volume effect, and a random term.

We adopt the approach by Silber, et al.[1-3] to estimate the risk adjusted scores; in addition, we also use a variant of the model described by Drye, at al.[4] and Norman, et al.[5] by adding hospital attributes. Let $\bar{y}$ be patient-level raw event rates in the sample dataset. We fit the model with logit link function, and obtain the site-specific predicted scores as

$$p_{ij} = E(Y_{ij} = 1 \big| X_{ij}, vol_i, b_{i0}) \ = \ 1/\{1 \ + \ exp(\text{-}\beta_0 - \textstyle\sum_{k=1}^{K} X_{ijk}\,\beta_k \ - \ \gamma log(vol_i) - b_{i0})\},$$

© 2020 Asch DA et al. JAMA Internal Medicine.

and the corresponding expected scores as

$$e_{ij} = E(Y_{ij} = 1 \big| X_{ij}, vol_i) = 1/\{1 + exp(\text{-}\beta_0 - \sum_{k=1}^{K} X_{ijk}\, \beta_k - \gamma log(vol_i))\}.$$

Directly standardized event rates, as defined in George, et al.[1] and Silber, et al.[2,3,4], are computed by averaging the event rates for all $N = \sum_{i=1}^{I} n_i$ had each one of them been treated hypothetically in every hospital. Denote $i*$ for a different hospital from $i$, define $p_{i*j} = E(Y_{i*j} = 1 \big| X_{ij}, vol_{i*}, b_{i*0}) = 1/\{1 + exp(\text{-}\beta_0 - \sum_{k=1}^{K} X_{ijk}\, \beta_k - \gamma log(vol_{i*}) - b_{i*0})\}$, where for each patient $j$ in the hospital $i*$, we have estimates for all $I$ hospitals such that $i^* = 1, \dots, I$. Next, directly standardized (DS) event rates for the $i^{\text{th}}$ hospital is computed as

$$s_{i,DS} = \sum_{i*=1}^{I} \sum_{j=1}^{n_{i*}} p_{i*j} \;/\; N.$$

Since $s_{i,DS}$ is an average over all $N$ patients, this eliminates any underlying effect arising from a different patient-mix distribution across sites. We refer the above directly standardized event rate as the score risk-standardized event rate (RSER) for a hospital in the paper.

As a sensitivity analysis, we also calculated the RSER measure using Dyre, et al.,[5] which was defined by the ratio of predicted to expected mortality or discharge to hospice, at a hospital, multiplied by the raw rate at the patient level for the entire sample. Specifically, denote by $s_{i,IS}$ the indirectly standardized (IS) hospital event rate for the $i^{\text{th}}$ hospital.

The standardized rates based on Drye, at al.[5] and Normand, et al.[6] for each hospital are calculated as a ratio of predicted and expected deaths multiplied by patient-level observed event rate for the dataset. Specifically, predicted number of deaths for each site is computed by using the estimated regression coefficients to the corresponding patient level characteristics, adding empirical best linear unbiased predictors (EBLUP), transforming link functions into probabilities, and summing them over all patients. Similarly, the rates for the $i^{\text{th}}$ hospital is calculated as

$$s_{i,IS} = (\sum_{j=1}^{n_i} p_{ij} \;/\; \sum_{j=1}^{n_i} e_{ij}) \times \bar{y}.$$

Note the main difference between $\boldsymbol{s_{i,DS}}$ and $\boldsymbol{s_{i,IS}}$ is that the former approach entails the probabilistic attributes and ensures ease of interpretability.

Here the estimation of fixed and random parameters are based on (restricted) maximum likelihood (RE)ML using both Laplace and adaptive Gaussian quadrature approximation techniques. While both methods provide comparable results, reported results thoughout the paper are based on the quadrature approach which entails approximating the integrals in the likelihood function and is theoretically more robust in reducing approximation error. Alternatively, a penalized quasi likelihood (PQL) method may also be adopted. Empirical best linear unbiased predictors (EBLUP) of random effects are obtained using a Bayesian formulation. We test the significance of the random effects by likelihood ratio test (LRT) that follows a mixture of Chi-squared distribution;[7,8] the corresponding p-value is <0.0001 implying the validity of using site-specific random effects. The estimated variance of the random effects is 0.19 with 95% CI (0.14, 0.24). We further investigate the marginal Akaike Information Criteria (AIC) values between GLM (25581.81) and GLMM (25414.20), and the corresponding marginal Bayesian Information Criteria (BIC) values are 25881.37 and 25589.22, respectively. To check collinearity diagnostics,

© 2020 Asch DA et al. JAMA Internal Medicine.

generalized variance inflation factors (< 2.00) are investigated for the fixed effects. The Spearman's rank correlation coefficient between the estimated fixed effect parameters based on GLMM and GLM is 1.00 (p-value < 0.0001) indicating high stability of regression coefficients. We check the goodness-of-fit of the model by conditional R-square (0.25), Somer's Dxy (0.48), and C-statistic (0.74). Adjusted odds ratios along with 95% confidence intervals and p-values based on Wald Z-statistic for testing the null effects are reported; see eFigure 2.

## Comparing hospital specific risk standardized event rates between early and later period

We cast the GLMM into a longitudinal framework to quantify the evolution of standardized rates over early (January 1, 2020-April 30, 2020) and late (May 1, 2020-June 30, 2020) period. We turn period as a binary time variable (0 = early and 1 = late). There are 398 sites matched between early and late period that have at least 10 COVID-19 hospitalization cases along with other filters for consistency. We fit a GLMM using site-specific random intercept and slope varying over time; we assume that random terms follow a bivariate normal distribution with mean zero and unknown non-diagonal covariance matrix. Let $s_{i0,DS}$ and $s_{i1,DS}$ refer to the risk-adjusted scores associated with early ($t_{ij} = 0$) and late time-period ($t_{ij} = 1$). For brevity and notational convenience, we use $t$ and $t_{ij}$ interchangeably. We write the association model as below

$$logit\ \Pr(Y_{ijt} = 1) = \beta_0 + \sum_{k=1}^{K+1} X_{ijkt}\,\beta_k + \gamma log(vol_{it}) + b_{i0} + b_{i1}t_{ij};$$

where $b_i = (b_{i0}, b_{i1})$ is the set of site-specific random intercept and slope effects. Assume $b_i$ follows multivariate Gaussian distribution mean zero and some non-diagonal covariance matrix. Note we have also added fixed effect of time. Subject-specific slope effects allow us to compute standardized risk scores at $t = 0$ and $t = 1$.

As before, we use Laplace and adaptive Gaussian quadrature approximation technique to obtain EBLUPs. Using the same method as was used to calculate the RSER earlier, we use a logit link function and obtain the predicted scores for the $i$-th subject at time $t$ as $p_{ijt} = 1/\{1 + exp(-\beta_0 - \sum_{k=1}^{K+1} X_{ijkt}\,\beta_k - \gamma log(\mathrm{vol}_{it}) - b_{i0} - b_{i1}t_{ij})\}$, and the corresponding expected rates are defined by $e_{ijt} = 1/\{1 + exp(-\beta_0 - \sum_{k=1}^{K+1} X_{ijkt}\,\beta_k - \gamma log(\mathrm{vol}_{it}))\}$. We obtain risk standardized event rates $p_{i*jt}$; $i^* = 1, \ldots I$, and based on these rates, we can compute the RSER for each hospital at each of the early- and late-periods.

To make inference about the differences between early and late scores, we perform a paired Wilcoxon signed rank test on RSERs estimated at the early- and late-periods, and obtained a p-value <0.0001, which suggested that the medians of RSERs at the early- and late-periods are statistically significantly different. We compute Kendall rank correlation coefficient (0.4731 with p-value <0.0001) to assess the degree of similarity between rankings of hospitals with respect to RSERs associated with early and late period.

## Association between hospital attributes and risk standardized event rates

Here the main objective is to quantify the association between hospital-level RSERs and hospital-attributes. We fit a linear model treating RSERs as continuous response variables while adjusting for hospital attributes (i.e., resident-to-bed ratio, number of ICU beds, number of beds, hospital settings, academic affiliation, and profit status) on 955 sites. To quantify strains of pandemic at different time points for different regions where hospitals are located, county-specific cases per 10K in the early period and relative change in cases between late- and early-period are used. We dichotomize the relative changes into a binary measure indicating 1 if cumulative cases increase in the late period, and 0

© 2020 Asch DA et al. JAMA Internal Medicine.

otherwise. We classify resident-to-bed ratio, number of ICU beds, number of beds, and cumulative cases per 10K during early period into discrete categories for ease of interpretability. Moreover, we add region (i.e., northeast, southeast, west, and central) as fixed effects to quantify the regional-specific variation in the associated hospital's responses. In addition, we include measures of the COVID-19 case load in the hospital's county measured as the number of cumulative cases per 10,000 residents as of May 1. We report the parameter estimates along with 95% confidence intervals and p-values based on Wald test.

As a sensitivity analysis, we also fit a hierarchical model (i.e., GLMM) as used before in computing patient-specific RSERs for all 955 hospitals. In contrast, we add both patient-specific, pandemic-strain specific, and hospital-attributes in a single model; the main objective is to quantify the extent of variation in patient-level event rates that is attributable to hospital attributes while adjusting for patient-specific risk factors. The appropriateness of using hospitals as random effects is evaluated via LRT (p-value < 0.0001). The spearman correlation coefficient between fixed-effect parameter estimates based on GLMM and an alternative model GLM (without random effect) is 1.00 (p-value < 0.0001). We check the goodness-of-fit of the GLMM by conditional R-square (0.26), Somer's Dxy (0.48), and C-statistic (0.74). We report the adjusted odds ratios along with 95% confidence intervals and p-values based on Wald test in eFigure 11.

Using the same set of hospital attributes as defined above, we fit a LM to characterize the variation in RSERs for early and late period associated with 398 sites; where we consider the differences between early and late period as our continuous response.

## Model based comparison between quintiles

For comparison between quintiles, we fit a univariate linear regression model with the risk standardized event rates (RSERs) as outcome and quintile group indicators as covariate. We choose quntile 1 as the reference quintile, which reflects the 20% of hospitals with the best risk standardized event rates.

## Illustration and codes

We created an artificial working dataset mimicking our analytical dataset. We illustrate the adopted methodologies for computing risk standardized event rates on this simulated data for the purpose of reproducibility and include the R-codes.

© 2020 Asch DA et al. JAMA Internal Medicine.

eFigure 1. Derivation and geographic distribution of the study sample.

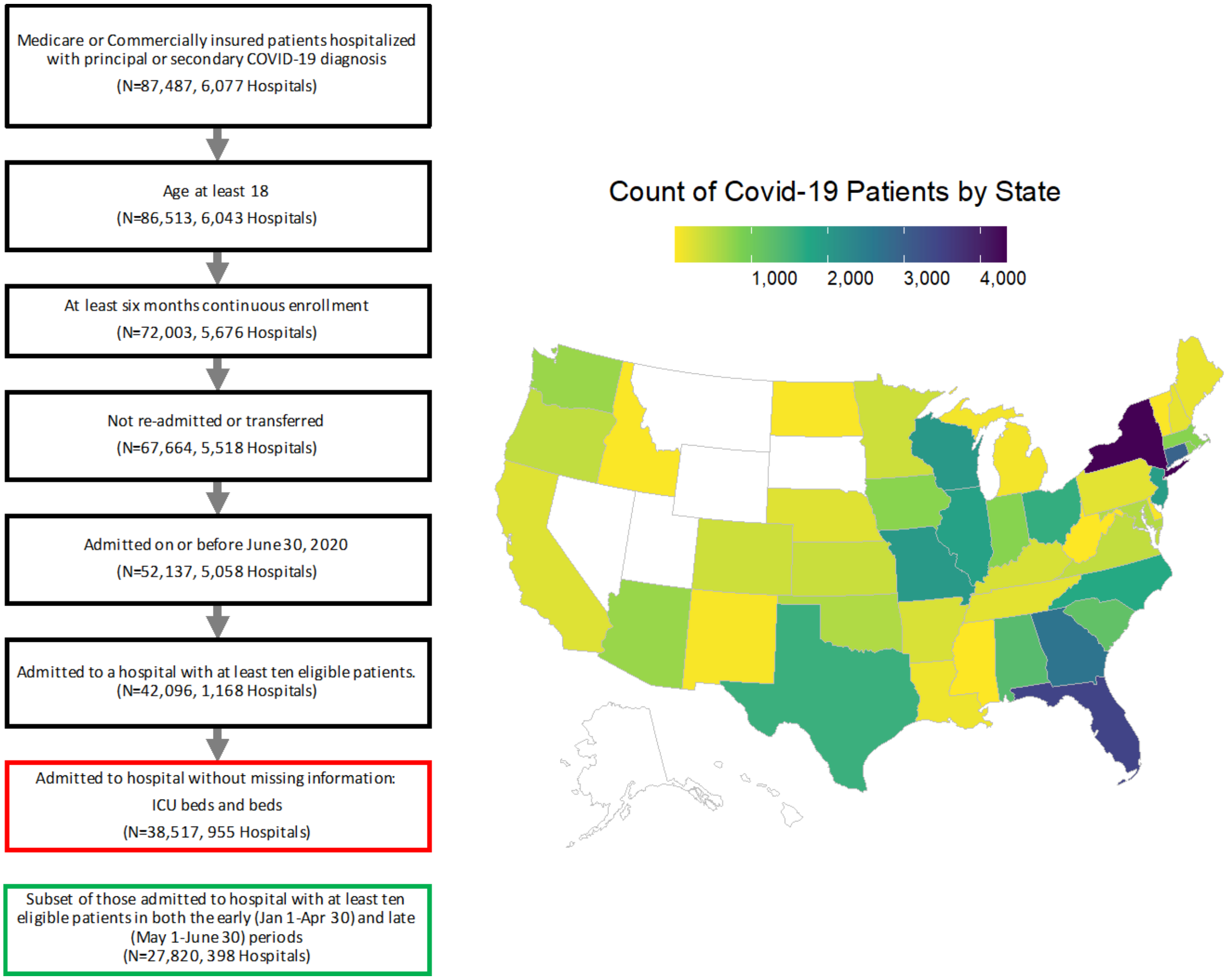


© 2020 Asch DA et al. JAMA Internal Medicine.

eFigure 2A. Estimated odds ratios of 30-day mortality or transfer to hospice for 955 hospitals and 38,517 patients.

| No. events/ COVID patients | Characteristic | Reference | Estimate (95% CI) | p-value |
|---|---|---|---|---|
| 144/3162 | Age::(45,55] | [18,45] | 2.62(1.88,3.65) | <0.0001 |
| 408/6044 | Age::(55,65] | [18,45] | 3.73(2.74,5.06) | <0.0001 |
| 1205/10654 | Age::(65,75] | [18,45] | 6.29(4.67,8.46) | <0.0001 |
| 1518/9698 | Age::(75,85] | [18,45] | 8.89(6.60,11.98) | <0.0001 |
| 1390/5974 | Age::>85 | [18,45] | 14.52(10.75,19.61) | <0.0001 |
| 2445/18888 | Gender::Male | Female | 1.29(1.20,1.38) | <0.0001 |
| 1286/4244 | Transferred from nursing facility::Yes | No | 2.43(2.22,2.65) | <0.0001 |
| 2001/12420 | Iron deficiency anemia::Yes | No | 1.13(1.05,1.22) | 0.0023 |
| 215/1574 | Blood loss anemia::Yes | No | 0.87(0.74,1.02) | 0.1088 |
| 1470/9663 | Congestive heart failure::Yes | No | 1.04(0.95,1.13) | 0.4242 |
| 1646/12654 | Chronic obstructive pulmonary disease::Yes | No | 0.99(0.92,1.06) | 0.7038 |
| 432/2786 | Coagulopathy::Yes | No | 1.11(0.99,1.25) | 0.0843 |
| 1092/8188 | Depression::Yes | No | 1.02(0.94,1.11) | 0.6540 |
| 1972/14139 | Diabetes without chronic complication::Yes | No | 1.02(0.93,1.11) | 0.7052 |
| 1678/12148 | Diabetes with chronic complication::Yes | No | 1.05(0.96,1.16) | 0.3122 |
| 99/1437 | Substance use disorder::Yes | No | 0.65(0.52,0.81) | 0.0002 |
| 3965/29335 | Hypertension::Yes | No | 0.96(0.87,1.06) | 0.4257 |
| 1198/8096 | Hypothyroidism::Yes | No | 1.01(0.94,1.10) | 0.7146 |
| 108/734 | Lymphoma::Yes | No | 1.07(0.85,1.33) | 0.5992 |
| 1449/10238 | Fluid & electrolyte disorder::Yes | No | 0.93(0.85,1.01) | 0.0719 |
| 287/1456 | Metastatic cancer::Yes | No | 1.85(1.57,2.18) | <0.0001 |
| 1547/9048 | Neurological disorder::Yes | No | 1.10(1.02,1.19) | 0.0220 |
| 1042/10100 | Obesity::Yes | No | 0.96(0.88,1.04) | 0.2956 |
| 320/1938 | Paralysis::Yes | No | 0.96(0.84,1.10) | 0.5757 |
| 1686/9899 | Peripheral vascular disease::Yes | No | 1.04(0.97,1.13) | 0.2329 |
| 394/2578 | Psychosis::Yes | No | 1.13(1.00,1.29) | 0.0629 |
| 1485/9832 | Chronic kidney disease::Yes | No | 0.95(0.88,1.03) | 0.2439 |
| 861/5352 | Solid tumor without metastasis::Yes | No | 1.10(0.99,1.21) | 0.0725 |
| 1044/7723 | Valvular disorder::Yes | No | 0.87(0.80,0.94) | 0.0013 |
| 659/3528 | Weight loss::Yes | No | 1.31(1.18,1.45) | <0.0001 |
| 676/3788 | Days from January 1,2020::[0,90) | ≥150 | 2.58(2.29,2.90) | <0.0001 |
| 1967/10876 | Days from January 1,2020::[90,120) | ≥150 | 2.28(2.08,2.50) | <0.0001 |
| 1185/11846 | Days from January 1,2020::[120,150) | ≥150 | 1.26(1.15,1.39) | <0.0001 |
| | Log(volume) | | 1.02(0.96,1.08) | 0.5690 |

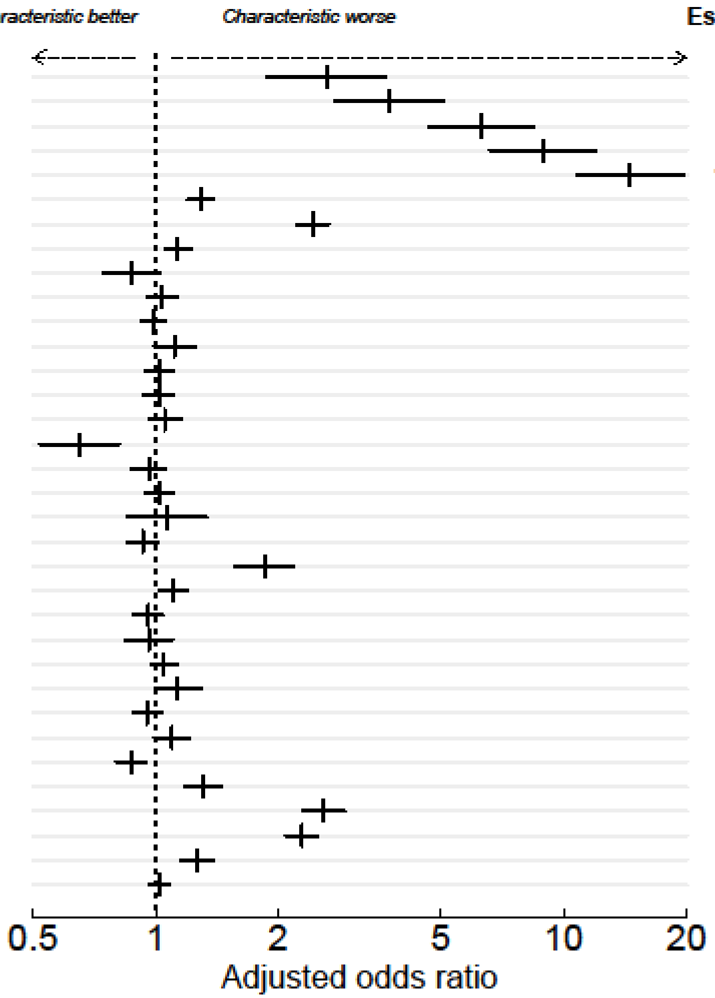


Log(volume) refers to the number of patients admitted to the hospital in our dataset. This variable is included in order to follow the model of Silber, et al[1-3] in order to compute the RSER for each hospital.

© 2020 Asch DA et al. JAMA Internal Medicine.

eFigure 2B. Estimated odds ratios of 30-day mortality for 955 hospitals and 38,517 patients. The analysis in eFigure 2A uses mortality or discharge to hospice as the outcome. The analysis in eFigure 2B uses only mortality.

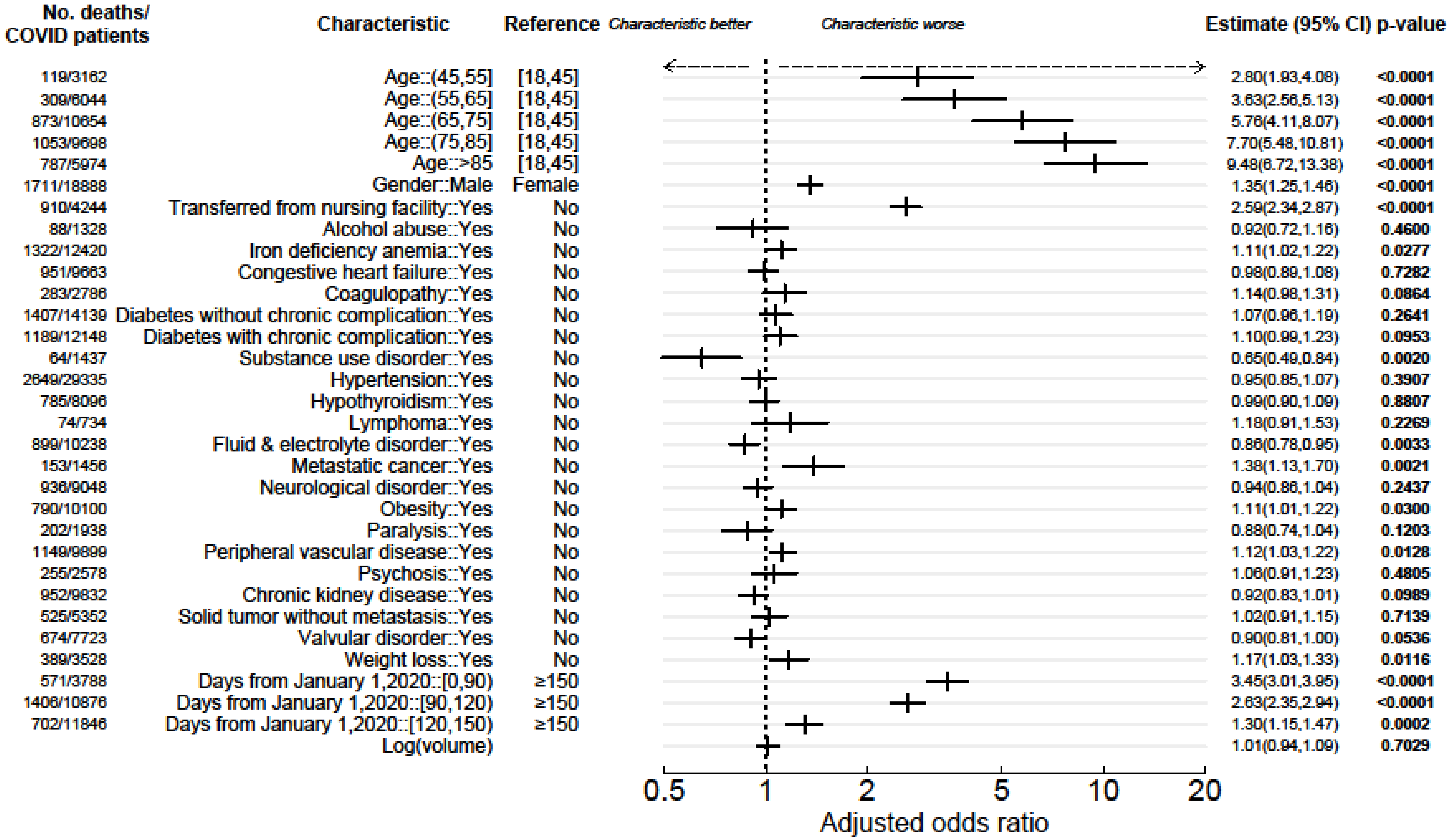


© 2020 Asch DA et al. JAMA Internal Medicine.

eFigure 3. Correlation of hospital rank based on the composite of mortality or hospice discharge or mortality alone.

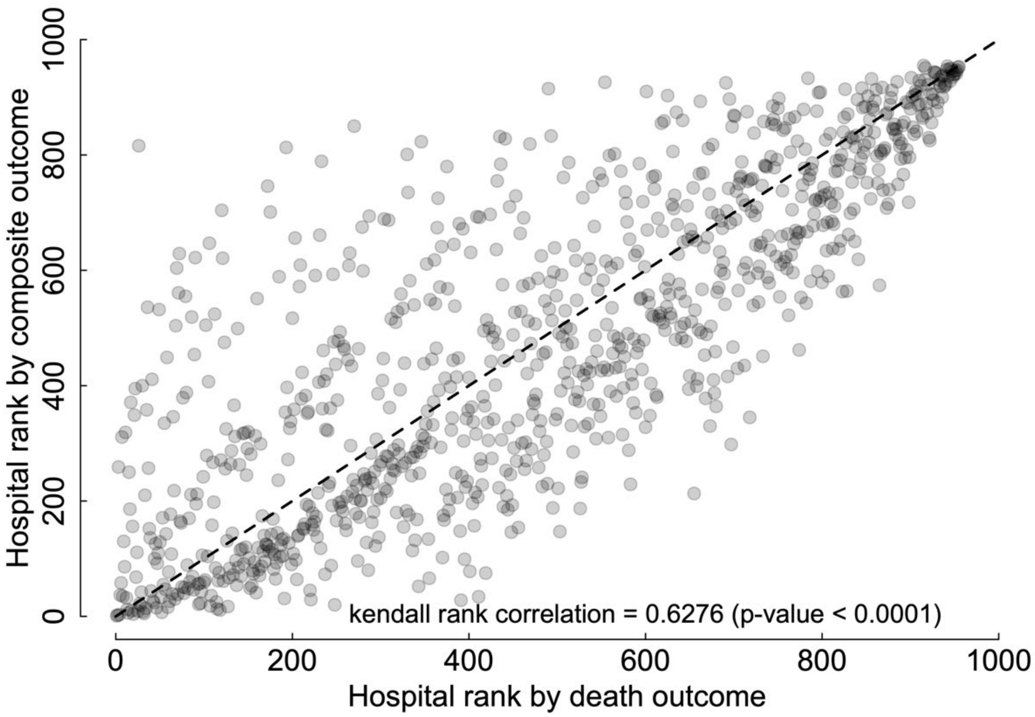


© 2020 Asch DA et al. JAMA Internal Medicine.

eFigure4. Distribution of risk standardized event rates for 955 hospitals and 38,517 patients

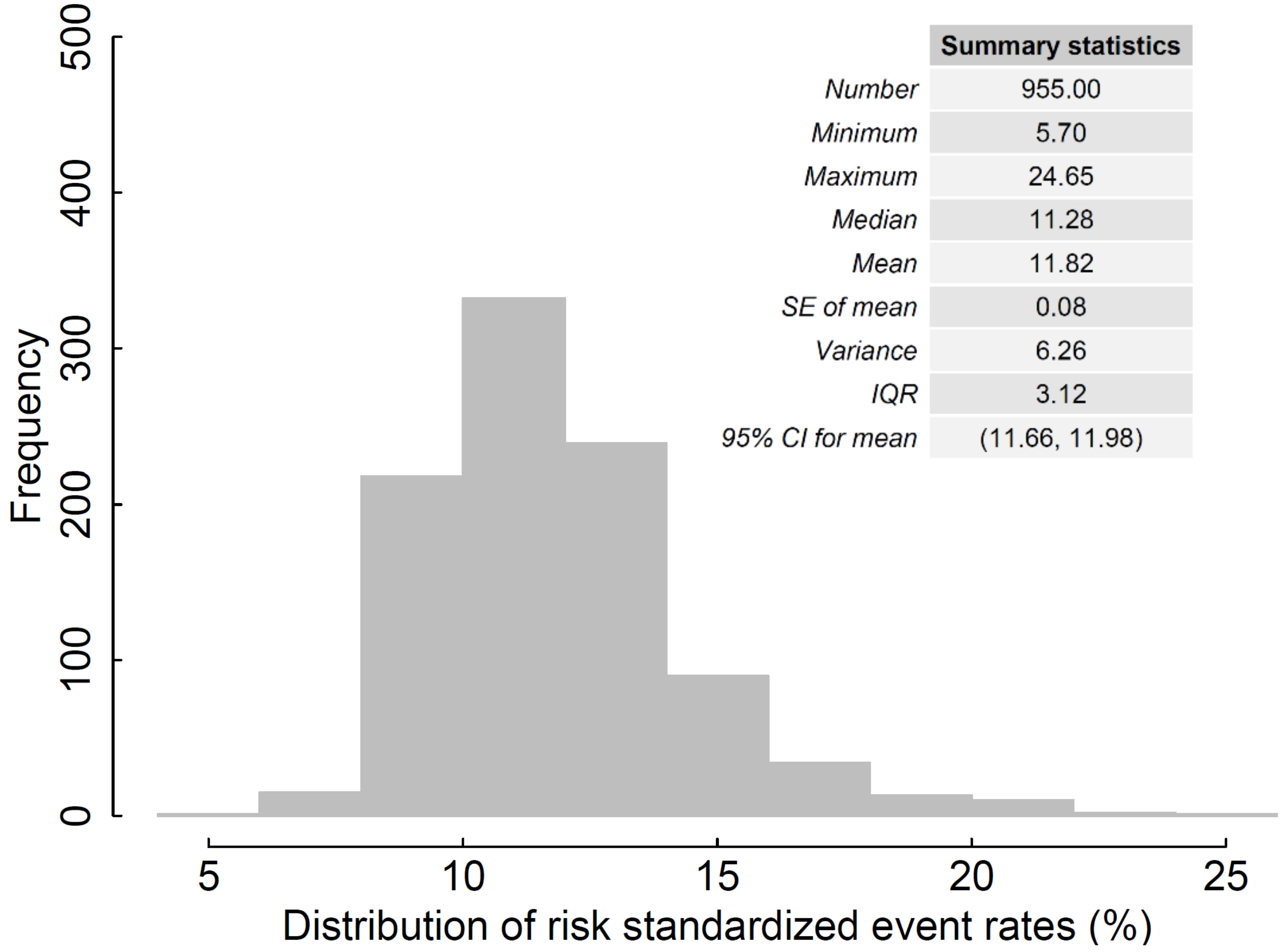


© 2020 Asch DA et al. JAMA Internal Medicine.

eFigure 5. Distribution of risk standardized event rates for 398 hospitals in the early period

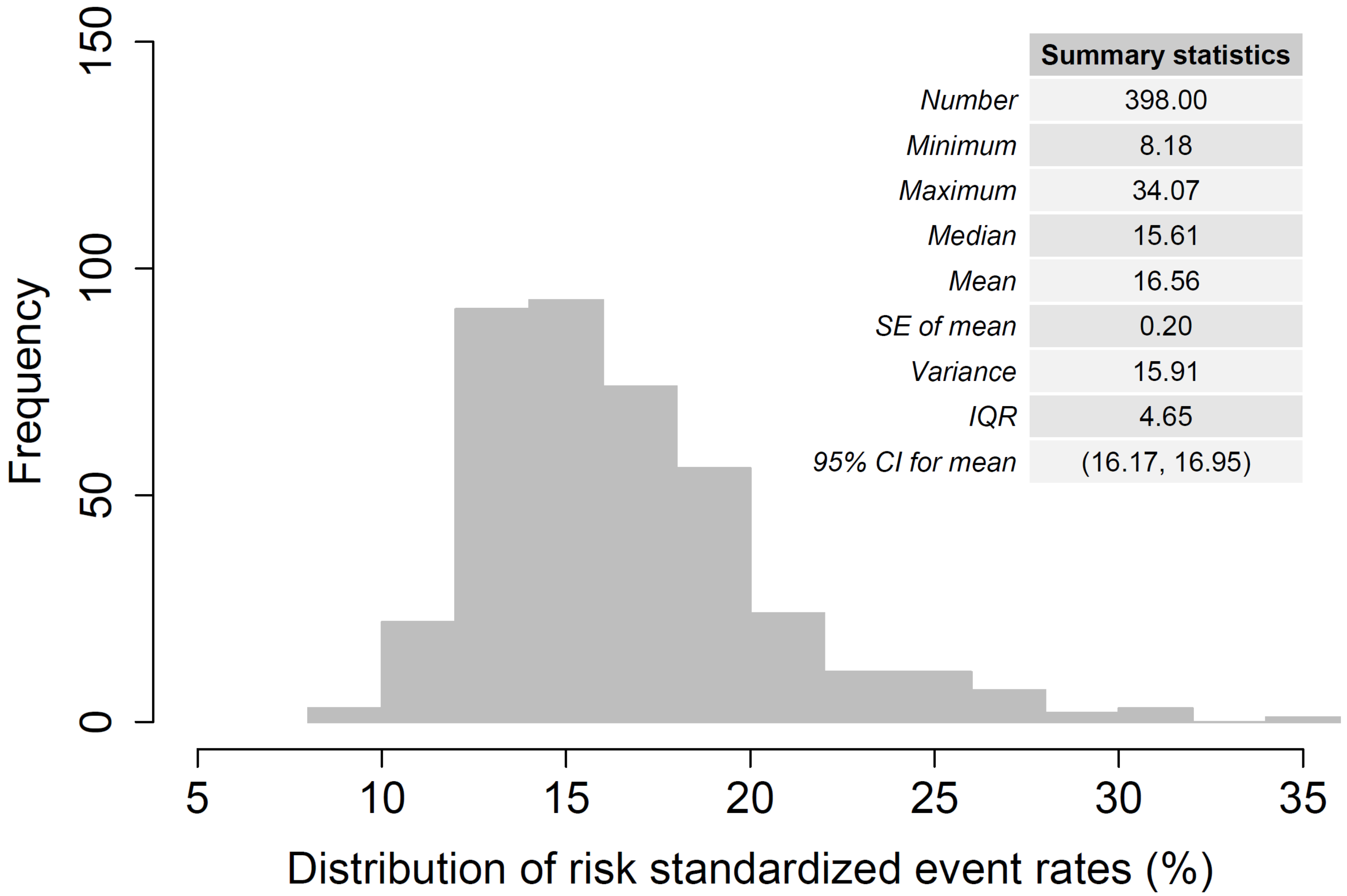


© 2020 Asch DA et al. JAMA Internal Medicine.

eFigure 6. Distribution of risk standardized event rates for 398 hospitals in the later period

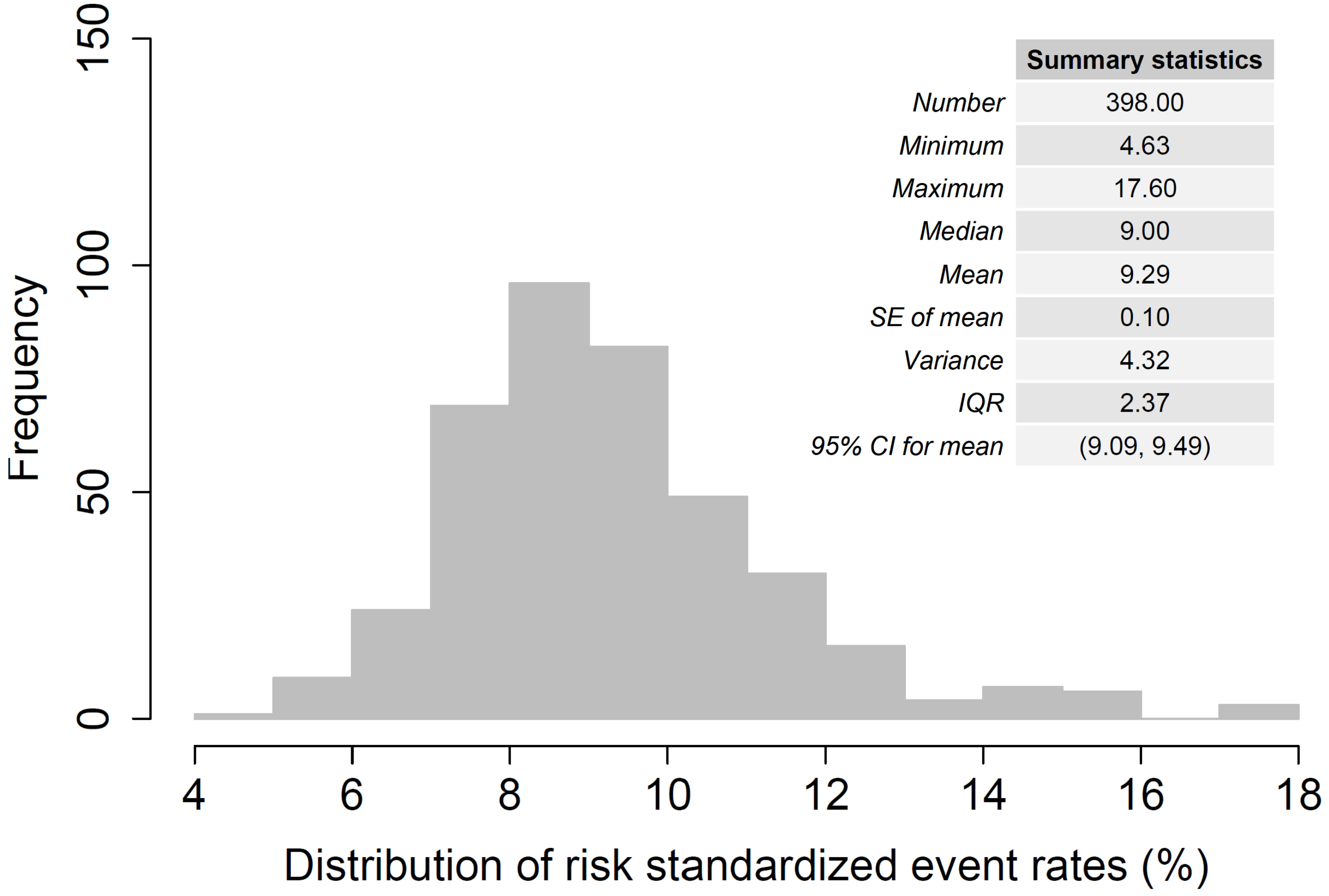


© 2020 Asch DA et al. JAMA Internal Medicine.

eFigure 7. Comparison of early and late period hospital-specific risk standardized rates for 30-day mortality or discharge to hospice for 398 hospitals and 27,801 patients. A higher score corresponds to worse performance.

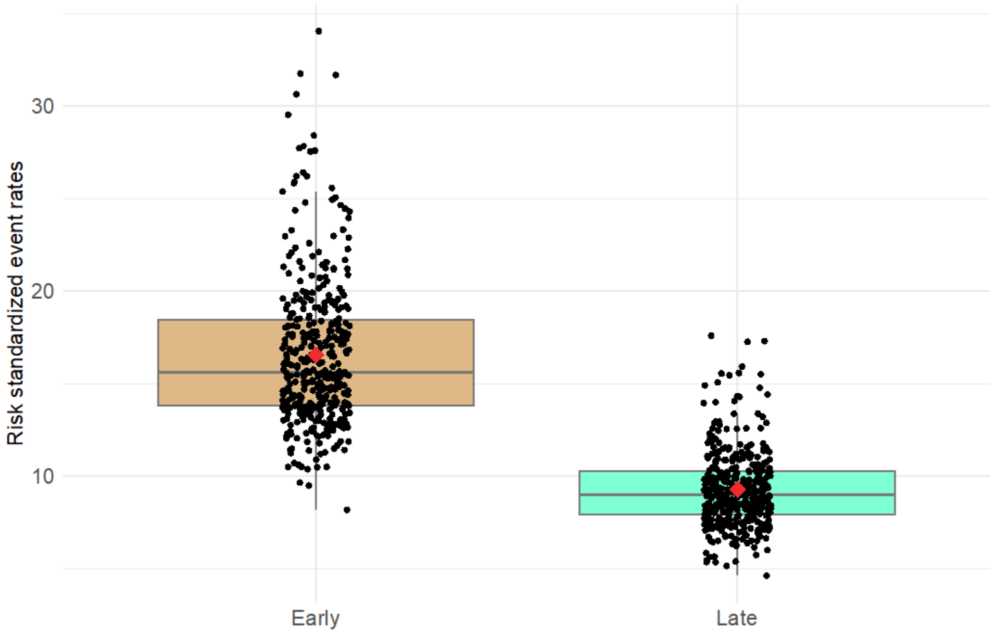


The late period (May 1-July 30, 2020) risk standardized event rates are in general lower and show a narrower range than the early period (Jan 1 to April 30, 2020) rates, suggesting both overall improvement and a reduction in variation across hospitals.

© 2020 Asch DA et al. JAMA Internal Medicine.

eFigure 8. Hospital characteristics associated with the risk standardized event rates of 955 hospitals.

| Size | Characteristic | Reference | Estimate (95% CI) | p-value |
|---|---|---|---|---|
| 346 | Number of ICU beds::[20,60) | [0,20) | 0.05 (-0.37, 0.47) | **0.8141** |
| 290 | Number of ICU beds::≥60 | [0,20) | 0.04 (-0.56, 0.63) | **0.9014** |
| 336 | Number of beds:[150,300) | [0,150) | 0.46 (-0.00, 0.92) | **0.0512** |
| 194 | Number of beds:[300,450) | [0,150) | 0.78 ( 0.21, 1.34) | **0.0074** |
| 203 | Number of beds::≥450 | [0,150) | 0.46 (-0.25, 1.16) | **0.2059** |
| 705 | Profit status::Non-profit | For profit | -0.04 (-0.50, 0.42) | **0.8633** |
| 111 | Profit status::Other | For profit | 0.26 (-0.35, 0.88) | **0.4029** |
| 892 | Location::Urban | Rural | -0.08 (-0.74, 0.58) | **0.8161** |
| 801 | Hospital affiliation::Non-academic | Academic | 0.44 (-0.09, 0.97) | **0.1048** |
| 207 | Cases per 10K:[20,40) | [0,20) | 0.22 (-0.19, 0.62) | **0.2985** |
| 297 | Cases per 10K::≥40 | [0,20) | 0.86 ( 0.43, 1.29) | **0.0001** |
| 264 | Region::North-east | Central | 0.69 ( 0.23, 1.14) | **0.0034** |
| 285 | Region::South-east | Central | 0.40 (-0.00, 0.80) | **0.0517** |
| 91 | Region::West | Central | 0.40 (-0.17, 0.96) | **0.1677** |

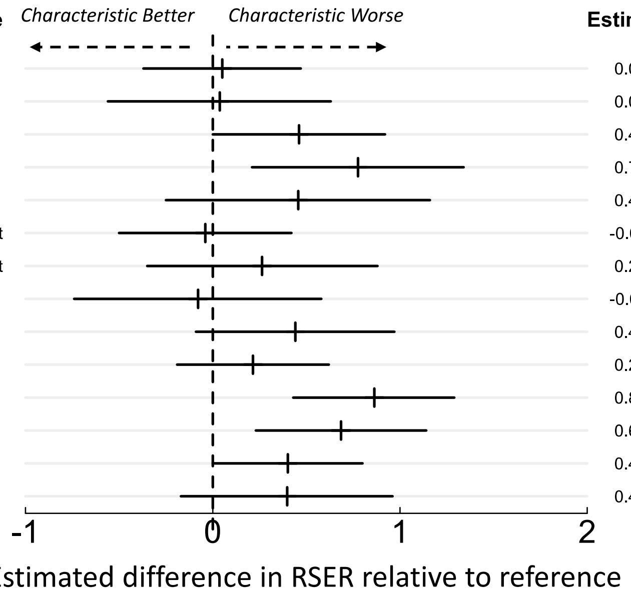


© 2020 Asch DA et al. JAMA Internal Medicine.

eFigure 9. Correlation of the direct standardized event rate used in this analysis and the indirect standardized event rate

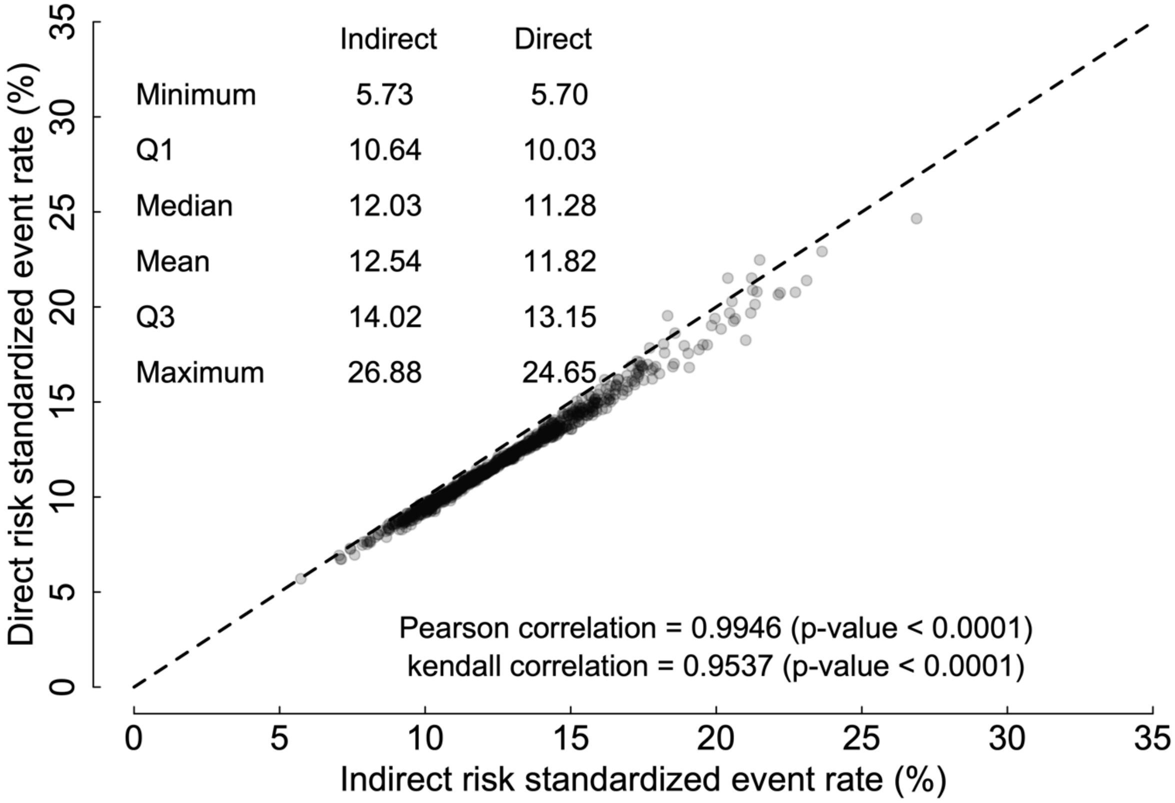


© 2020 Asch DA et al. JAMA Internal Medicine.

eFigure 10. Correlation of the ranking for hospitals in the early and later periods

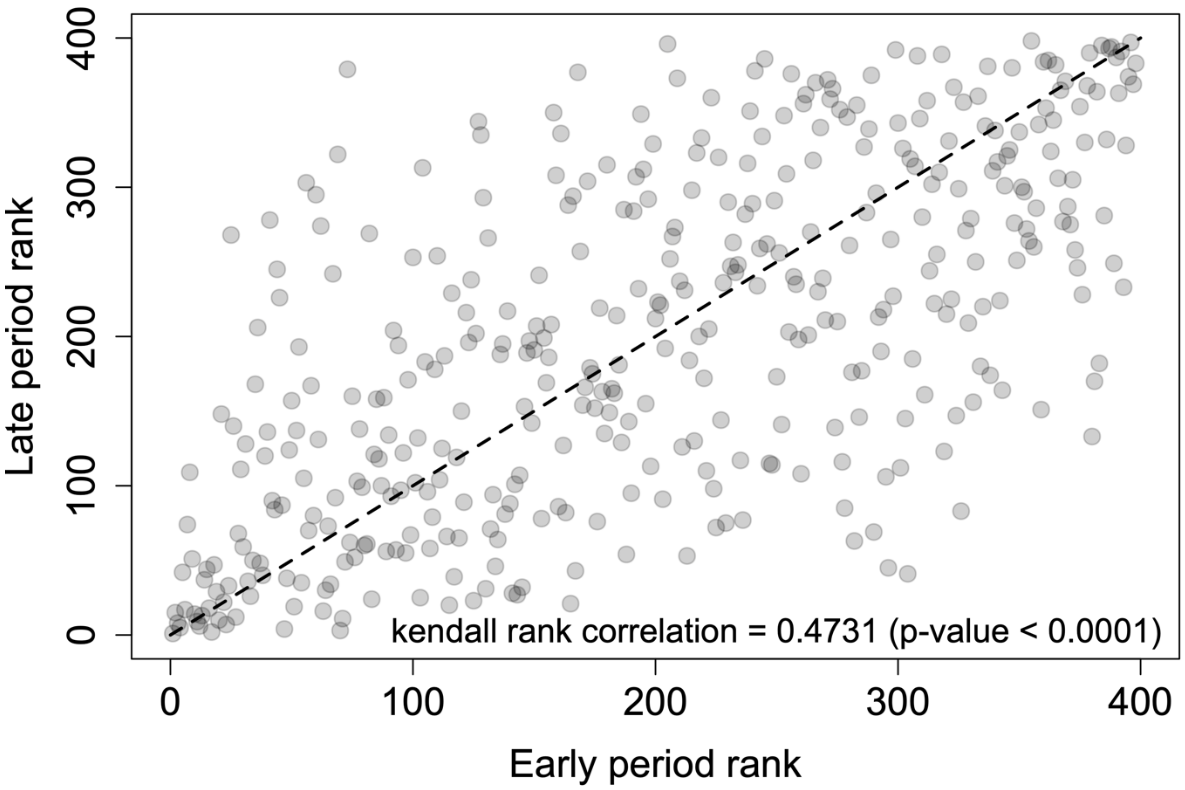


Kendall rank correlation coefficient reflecting the similarity of each hospital's relative position in RSER between early and later periods. The coefficient value of 0.4731 reveals that hospitals with low (better) RSERs in the early period tend to have low (better) RSERs in the later period and vice versa.

© 2020 Asch DA et al. JAMA Internal Medicine.

eFigure 11. A hierarchical model reflecting both patient-and-hospital attributes. This analysis reveals how much variation in patient-level mortality can be explained by hospital attributes.

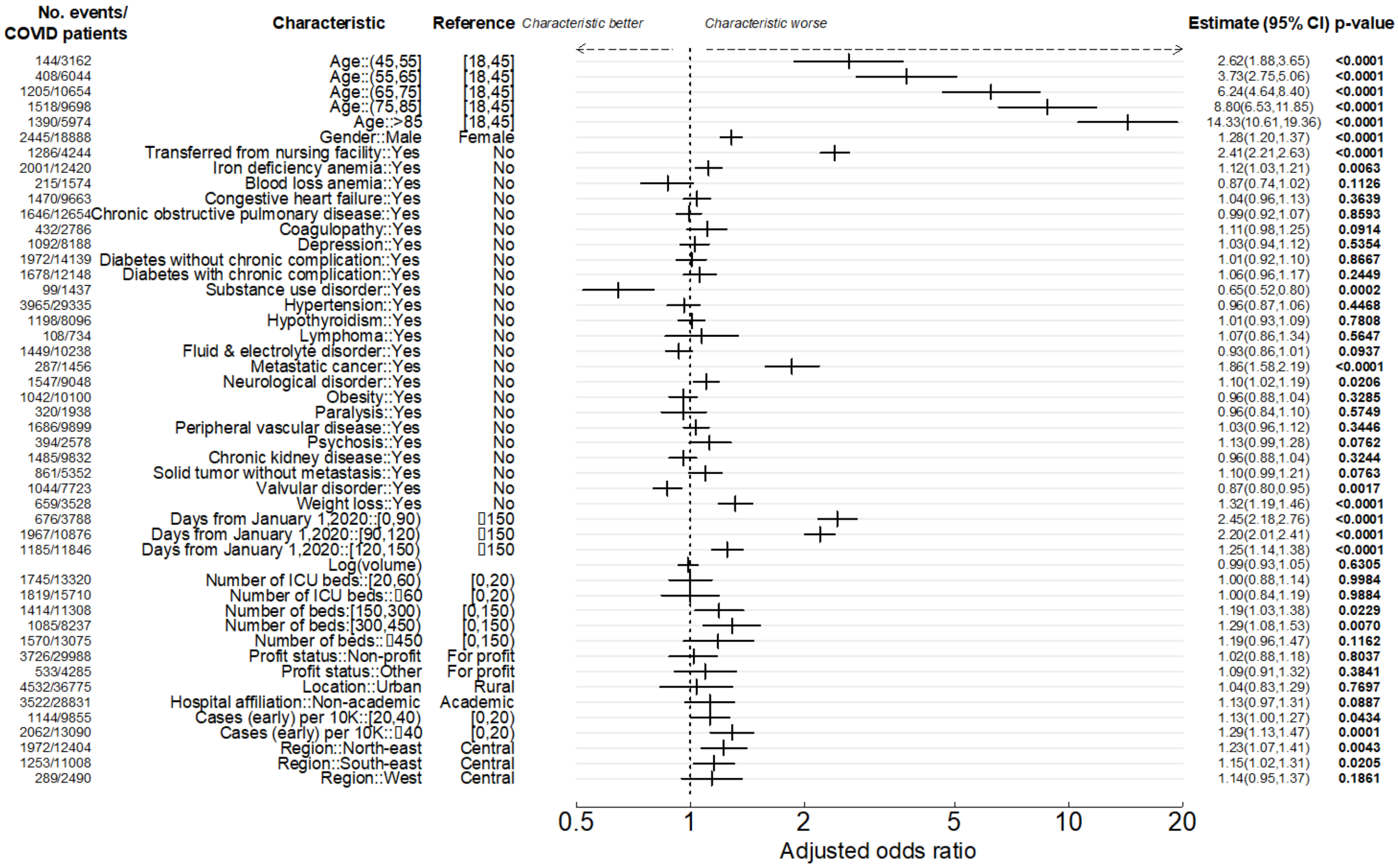


© 2020 Asch DA et al. JAMA Internal Medicine.

eTable 1A. Difference in the 30-day mortality or hospice discharge (Risk standardized event rate, RSER) compared to the best performing quintile (Q1) overall (955 hospitals) and early and late periods (398 hospitals). Q1 is the reference quintile reflecting the 20% of hospitals with the best (lowest) risk standardized event rates. For example, the bottom right cell implies that hospitals' rate of mortality or discharge to hospice in the worst performing quintile (Q5) is 5.59 percentage points greater than those in the best performing quintile (Q1).

| | **Overall** | | **Early Period** | | **Late Period** | |
|---|---|---|---|---|---|---|
| Quintile | RSER Increase | Confidence Interval | RSER Increase | Confidence Interval | RSER Increase | Confidence Interval |
| Q1 | Reference | | Reference | | Reference | |
| Q2 | 1.22 | (1.01-1.43) | 1.94 | (1.43-2.45) | 1.24 | (0.97-1.50) |
| Q3 | 2.29 | (2.08-2.51) | 3.59 | (3.07-4.10) | 2.11 | (1.84-2.37) |
| Q4 | 3.68 | (3.47-3.89) | 5.76 | (5.24-6.27) | 3.11 | (2.85-3.38) |
| Q5 | 6.59 | (6.38-6.80) | 10.54 | (10.03-11.05) | 5.59 | (5.33-5.86) |

eTable 1B. Difference in the 30-day mortality (Risk standardized mortality rate, RSMR) compared to the best performing quintile (Q1) overall (955 hospitals) and early and late periods (398 hospitals). Q1 is the reference quintile reflecting the 20% of hospitals with the best (lowest) risk standardized mortality rates. For example, the bottom right cell implies that hospitals' rate of mortality in the worst performing quintile (Q5) is 5.36 percentage points greater than those in the best performing quintile (Q1).

| | **Overall** | | **Early Period** | | **Late Period** | |
|---|---|---|---|---|---|---|
| Quintile | RSMR Increase | Confidence Interval | RSMR Increase | Confidence Interval | RSMR Increase | Confidence Interval |
| Q1 | Reference | | Reference | | Reference | |
| Q2 | 0.95 | (0.71-1.20) | 1.94 | (1.43-2.46) | 0.88 | (0.63-1.23) |
| Q3 | 1.91 | (1.66-2.15) | 3.60 | (3.09-4.11) | 1.80 | (1.55-2.05) |
| Q4 | 3.26 | (3.01-3.50) | 5.84 | (5.32-6.35) | 2.81 | (2.56-3.06) |
| Q5 | 6.71 | (6.46-6.95) | 11.35 | (10.84-11.86) | 5.36 | (5.11-5.61) |

© 2020 Asch DA et al. JAMA Internal Medicine.

eTable 2. Risk standardized 30-day mortality or discharge to hospice rates stratified by county-level cumulative reported COVID-19 case burden as of April 30, 2020 (955 hospitals)

| | **[0,20) Cases/ 10K people** | | **[20,40) Cases/ 10K people** | | **≥40 Cases/ 10K people** | |
|---|---|---|---|---|---|---|
| Quintile | Mean RSER | Confidence Interval | Mean RSER | Confidence Interval | Mean RSER | Confidence Interval |
| Q1 | 9.15% | (9.04%-9.25%) | 9.03% | (8.79%-9.28%) | 8.88% | (8.59%-9.16%) |
| Q2 | 10.30% | (10.24%-10.36%) | 10.24% | (10.16%-10.32%) | 10.30% | (10.21%-10.39%) |
| Q3 | 11.33% | (11.27%-11.40%) | 11.39% | (11.28%-11.49%) | 11.39% | (11.28%-11.50%) |
| Q4 | 12.74% | (12.65%-12.84%) | 12.81% | (12.66%-12.97%) | 12.70% | (12.59%-12.81%) |
| Q5 | 15.23% | (14.74%-15.72%) | 15.60% | (14.97%-16.23%) | 15.99% | (15.50%-16.47%) |

© 2020 Asch DA et al. JAMA Internal Medicine.

eTable 3. ICD10 codes used in the analyses

**Inclusion Criteria**

| Inclusion Criteria | ICD-10-CM codes |
|---|---|
| COVID-19 | U071, U072, B9729 |

## Elixhauser Comorbidity Indices

| Elixhauser Comorbidity Index | ICD-10-CM codes |
|---|---|
| Acquired immunodeficiency Syndrome | B20 |
| Alcohol Use Disorder | F1010, F1011, F10120, F10121, F10129, F1014, F10150, F10151, F10159, F10180, F10181, F10182, F10188, F1019, F1020, F1021, F10220, F10221, F10229, F10230, F10231, F10232, F10239, F1024, F10250, F10251, F10259, F1026, F1027, F10280, F10281, F10282, F10288, F1029, F10921, F1094, F10950, F10951, F10959, F1096, F1097, F10980, F10981, F10982, F10988, F1099 |
| Iron Deficiency Anemia | D501, D508, D509, D510, D511, D512, D513, D518, D519, D520, D521, D528, D529, D530, D531, D532, D538, D539, D630, D631, D638, D649 |
| Rheumatoid Arthritis | L900, L940, L941, L943, M0500, M05011, M05012, M05019, M05021, M05022, M05029, M05031, M05032, M05039, M05041, M05042, M05049, M05051, M05052, M05059, M05061, M05062, M05069, M05071, M05072, M05079, M0509, M0510, M05111, M05112, M05119, M05121, M05122, M05129, M05131, M05132, M05139, M05141, M05142, M05149, M05151, M05152, M05159, M05161, M05162, M05169, M05171, M05172, M05179, M0519, M0520, M05211, M05212, M05219, M05221, M05222, M05229, M05231, M05232, M05239, M05241, M05242, M05249, M05251, M05252, M05259, M05261, M05262, M05269, M05271, M05272, M05279, M0529, M0530, M05311, M05312, M05319, M05321, M05322, M05329, M05331, M05332, M05339, M05341, M05342, M05349, M05351, M05352, M05359, M05361, M05362, M05369, M05371, M05372, M05379, M0539, M0540, M05411, M05412, M05419, M05421, M05422, M05429, M05431, M05432, M05439, M05441, M05442, M05449, M05451, M05452, M05459, M05461, M05462, M05469, M05471, M05472, M05479, M0549, M0550, M05511, M05512, M05519, M05521, M05522, M05529, M05531, M05532, M05539, M05541, M05542, M05549, M05551, M05552, M05559, M05561, M05562, M05569, M05571, M05572, M05579, M0559, M0560, M05611, M05612, M05619, M05621, M05622, M05629, M05631, M05632, M05639, M05641, M05642, M05649, M05651, M05652, M05659, M05661, M05662, M05669, M05671, M05672, M05679, M0569, M0570, M05711, M05712, M05719, M05721, M05722, M05729, M05731, M05732, M05739, M05741, M05742, M05749, M05751, M05752, M05759, M05761, M05762, M05769, M05771, M05772, M05779, M0579, M0580, M05811, M05812, M05819, M05821, M05822, M05829, M05831, M05832, M05839, M05841, M05842, M05849, M05851, M05852, M05859, M05861, M05862, M05869, M05871, M05872, M05879, M0589, M059, M0600, M06011, M06012, M06019, M06021, M06022, M06029, M06031, M06032, M06039, M06041, M06042, M06049, M06051, M06052, M06059, M06061, M06062, M06069, M06071, M06072, M06079, M0608, M0609, M061, M0620, M06211, M06212, M06219, M06221, M06222, M06229, M06231, M06232, M06239, M06241, M06242, M06249, M06251, M06252, M06259, M06261, M06262, M06269, M06271, M06272, M06279, M0628, M0629, M0630, M06311, M06312, M06319, M06321, M06322, M06329, M06331, M06332, M06339, M06341, M06342, M06349, M06351, M06352, M06359, M06361, M06362, M06369, M06371, M06372, M06379, M0638, M0639, M064, M0680, M06811, M06812, M06819, M06821, M06822, M06829, M06831, M06832, M06839, M06841, M06842, M06849, M06851, M06852, M06859, M06861, M06862, M06869, M06871, M06872, M06879, M0688, M0689, M069, M0800, M08011, M08012, M08019, M08021, M08022, M08029, M08031, M08032, M08039, M08041, M08042, M08049, M08051, M08052, M08059, M08061, M08062, M08069, M08071, |

© 2020 Asch DA et al. JAMA Internal Medicine.

| | |
|---|---|
| | M08072, M08079, M0808, M0809, M081, M0820, M08211, M08212, M08219, M08221, M08222, M08229, M08231, M08232, M08239, M08241, M08242, M08249, M08251, M08252, M08259, M08261, M08262, M08269, M08271, M08272, M08279, M0828, M0829, M083, M0840, M08411, M08412, M08419, M08421, M08422, M08429, M08431, M08432, M08439, M08441, M08442, M08449, M08451, M08452, M08459, M08461, M08462, M08469, M08471, M08472, M08479, M0848, M0880, M08811, M08812, M08819, M08821, M08822, M08829, M08831, M08832, M08839, M08841, M08842, M08849, M08851, M08852, M08859, M08861, M08862, M08869, M08871, M08872, M08879, M0888, M0889, M0890, M08911, M08912, M08919, M08921, M08922, M08929, M08931, M08932, M08939, M08941, M08942, M08949, M08951, M08952, M08959, M08961, M08962, M08969, M08971, M08972, M08979, M0898, M0899, M1200, M12011, M12012, M12019, M12021, M12022, M12029, M12031, M12032, M12039, M12041, M12042, M12049, M12051, M12052, M12059, M12061, M12062, M12069, M12071, M12072, M12079, M1208, M1209, M320, M3210, M3211, M3212, M3213, M3214, M3215, M3219, M328, M329, M3300, M3301, M3302, M3303, M3309, M3310, M3311, M3312, M3313, M3319, M3320, M3321, M3322, M3329, M3390, M3391, M3392, M3393, M3399, M340, M341, M342, M3481, M3482, M3483, M3489, M349, M3500, M3501, M3502, M3503, M3504, M3509, M351, M353, M355, M358, M359, M360, M368, M450, M451, M452, M453, M454, M455, M456, M457, M458, M459, M4600, M4601, M4602, M4603, M4604, M4605, M4606, M4607, M4608, M4609, M461, M4650, M4651, M4652, M4653, M4654, M4655, M4656, M4657, M4658, M4659, M4680, M4681, M4682, M4683, M4684, M4685, M4686, M4687, M4688, M4689, M4690, M4691, M4692, M4693, M4694, M4695, M4696, M4697, M4698, M4699, M488X1, M488X2, M488X3, M488X4, M488X5, M488X6, M488X7, M488X8, M488X9, M4980, M4981, M4982, M4983, M4984, M4985, M4986, M4987, M4988, M4989 |
| Blood Loss Anemia | D500, O9081, O99011, O99012, O99013, O99019, O9902, O9903 |
| Congestive Heart Failure | I0981, I110, I130, I132, I501, I5020, I5021, I5022, I5023, I5030, I5031, I5032, I5033, I5040, I5041, I5042, I5043, I50810, I50811, I50812, I50813, I50814, I5082, I5083, I5084, I5089, I509 |
| Chronic Obstructive Pulmonary Disease | J40, J410, J411, J418, J42, J430, J431, J432, J438, J439, J440, J441, J449, J4520, J4521, J4522, J4530, J4531, J4532, J4540, J4541, J4542, J4550, J4551, J4552, J45901, J45902, J45909, J45990, J45991, J45998, J470, J471, J479, J60, J61, J620, J628, J630, J631, J632, J633, J634, J635, J636, J64, J660, J661, J662, J668, J670, J671, J672, J673, J674, J675, J676, J677, J678, J679, J684 |
| Coagulopathy | D65, D66, D67, D680, D681, D682, D68311, D68312, D68318, D6832, D684, D688, D689, D691, D693, D6941, D6942, D6949, D6951, D6959, D696, D7582, O99111, O99112, O99113, O99119, O9912, O9913 |
| Depression | F320, F321, F322, F323, F328, F3281, F3289, F329, F330, F331, F332, F333, F338, F339, F341, F4321 |
| Diabetes without Chronic Complications | E0800, E0801, E0810, E0811, E089, E0900, E0901, E0910, E0911, E099, E1010, E1011, E109, E1100, E1101, E1110, E1111, E119, E1300, E1301, E1310, E1311, E139, O24011, O24012, O24013, O24019, O2402, O2403, O24111, O24112, O24113, O24119, O2412, O2413, O24311, O24312, O24313, O24319, O2432, O2433, O24811, O24812, O24813, O24819, O2482, O2483, O24911, O24912, O24913, O24919, O2492, O2493 |

© 2020 Asch DA et al. JAMA Internal Medicine.

| | |
|---|---|
| Diabetes with Chronic Complications | E0821, E0822, E0829, E08311, E08319, E08321, E083211, E083212, E083213, E083219, E08329, E083291, E083292, E083293, E083299, E08331, E083311, E083312, E083313, E083319, E08339, E083391, E083392, E083393, E083399, E08341, E083411, E083412, E083413, E083419, E08349, E083491, E083492, E083493, E083499, E08351, E083511, E083512, E083513, E083519, E083521, E083522, E083523, E083529, E083531, E083532, E083533, E083539, E083541, E083542, E083543, E083549, E083551, E083552, E083553, E083559, E08359, E083591, E083592, E083593, E083599, E0836, E0837X1, E0837X2, E0837X3, E0837X9, E0839, E0840, E0841, E0842, E0843, E0844, E0849, E0851, E0852, E0859, E08610, E08618, E08620, E08621, E08622, E08628, E08630, E08638, E08641, E08649, E0865, E0869, E088, E0921, E0922, E0929, E09311, E09319, E09321, E093211, E093212, E093213, E093219, E09329, E093291, E093292, E093293, E093299, E09331, E093311, E093312, E093313, E093319, E09339, E093391, E093392, E093393, E093399, E09341, E093411, E093412, E093413, E093419, E09349, E093491, E093492, E093493, E093499, E09351, E093511, E093512, E093513, E093519, E093521, E093522, E093523, E093529, E093531, E093532, E093533, E093539, E093541, E093542, E093543, E093549, E093551, E093552, E093553, E093559, E09359, E093591, E093592, E093593, E093599, E0936, E0937X1, E0937X2, E0937X3, E0937X9, E0939, E0940, E0941, E0942, E0943, E0944, E0949, E0951, E0952, E0959, E09610, E09618, E09620, E09621, E09622, E09628, E09630, E09638, E09641, E09649, E0965, E0969, E098, E1021, E1022, E1029, E10311, E10319, E10321, E103211, E103212, E103213, E103219, E10329, E103291, E103292, E103293, E103299, E10331, E103311, E103312, E103313, E103319, E10339, E103391, E103392, E103393, E103399, E10341, E103411, E103412, E103413, E103419, E10349, E103491, E103492, E103493, E103499, E10351, E103511, E103512, E103513, E103519, E103521, E103522, E103523, E103529, E103531, E103532, E103533, E103539, E103541, E103542, E103543, E103549, E103551, E103552, E103553, E103559, E10359, E103591, E103592, E103593, E103599, E1036, E1037X1, E1037X2, E1037X3, E1037X9, E1039, E1040, E1041, E1042, E1043, E1044, E1049, E1051, E1052, E1059, E10610, E10618, E10620, E10621, E10622, E10628, E10630, E10638, E10641, E10649, E1065, E1069, E108, E1121, E1122, E1129, E11311, E11319, E11321, E113211, E113212, E113213, E113219, E11329, E113291, E113292, E113293, E113299, E11331, E113311, E113312, E113313, E113319, E11339, E113391, E113392, E113393, E113399, E11341, E113411, E113412, E113413, E113419, E11349, E113491, E113492, E113493, E113499, E11351, E113511, E113512, E113513, E113519, E113521, E113522, E113523, E113529, E113531, E113532, E113533, E113539, E113541, E113542, E113543, E113549, E113551, E113552, E113553, E113559, E11359, E113591, E113592, E113593, E113599, E1136, E1137X1, E1137X2, E1137X3, E1137X9, E1139, E1140, E1141, E1142, E1143, E1144, E1149, E1151, E1152, E1159, E11610, E11618, E11620, E11621, E11622, E11628, E11630, E11638, E11641, E11649, E1165, E1169, E118, E1321, E1322, E1329, E13311, E13319, E13321, E133211, E133212, E133213, E133219, E13329, E133291, E133292, E133293, E133299, E13331, E133311, E133312, E133313, E133319, E13339, E133391, E133392, E133393, E133399, E13341, E133411, E133412, E133413, E133419, E13349, E133491, E133492, E133493, E133499, E13351, E133511, E133512, E133513, E133519, E133521, E133522, E133523, E133529, E133531, E133532, E133533, E133539, E133541, E133542, E133543, E133549, E133551, E133552, E133553, E133559, E13359, E133591, E133592, E133593, E133599, E1336, E1337X1, E1337X2, E1337X3, E1337X9, E1339, E1340, E1341, E1342, E1343, E1344, E1349, E1351, E1352, E1359, E13610, E13618, E13620, E13621, E13622, E13628, E13630, E13638, E13641, E13649, E1365, E1369, E138, P702 |

© 2020 Asch DA et al. JAMA Internal Medicine.

| | |
|---|---|
| Substance Use Disorder | F1110, F1111, F11120, F11121, F11122, F11129, F1114, F11150, F11151, F11159, F11181, F11182, F11188, F1119, F1120, F1121, F11220, F11221, F11222, F11229, F1123, F1124, F11250, F11251, F11259, F11281, F11282, F11288, F1129, F1210, F1211, F12120, F12121, F12122, F12129, F12150, F12151, F12159, F12180, F12188, F1219, F1220, F1221, F12220, F12221, F12222, F12229, F1223, F12250, F12251, F12259, F12280, F12288, F1229, F1310, F1311, F13120, F13121, F13129, F1314, F13150, F13151, F13159, F13180, F13181, F13182, F13188, F1319, F1320, F1321, F13220, F13221, F13229, F13230, F13231, F13232, F13239, F1324, F13250, F13251, F13259, F1326, F1327, F13280, F13281, F13282, F13288, F1329, F1410, F1411, F14120, F14121, F14122, F14129, F1414, F14150, F14151, F14159, F14180, F14181, F14182, F14188, F1419, F1420, F1421, F14220, F14221, F14222, F14229, F1423, F1424, F14250, F14251, F14259, F14280, F14281, F14282, F14288, F1429, F1510, F1511, F15120, F15121, F15122, F15129, F1514, F15150, F15151, F15159, F15180, F15181, F15182, F15188, F1519, F1520, F1521, F15220, F15221, F15222, F15229, F1523, F1524, F15250, F15251, F15259, F15280, F15281, F15282, F15288, F1529, F1610, F1611, F16120, F16121, F16122, F16129, F1614, F16150, F16151, F16159, F16180, F16183, F16188, F1619, F1620, F1621, F16220, F16221, F16229, F1624, F16250, F16251, F16259, F16280, F16283, F16288, F1629, F1810, F1811, F18120, F18121, F18129, F1814, F18150, F18151, F18159, F1817, F18180, F18188, F1819, F1820, F1821, F18220, F18221, F18229, F1824, F18250, F18251, F18259, F1827, F18280, F18288, F1829, F1910, F1911, F19120, F19121, F19122, F19129, F1914, F19150, F19151, F19159, F1916, F1917, F19180, F19181, F19182, F19188, F1919, F1920, F1921, F19220, F19221, F19222, F19229, F19230, F19231, F19232, F19239, F1924, F19250, F19251, F19259, F1926, F1927, F19280, F19281, F19282, F19288, F1929, F550, F551, F552, F553, F554, F558, O99320, O99321, O99322, O99323, O99324, O99325 |
| Hypertension | I10, I110, I119, I120, I129, I130, I1310, I1311, I132, I150, I151, I152, I158, I159, I160, I161, I169, I674, O10011, O10012, O10013, O10019, O1002, O1003, O10111, O10112, O10113, O10119, O1012, O1013, O10211, O10212, O10213, O10219, O1022, O1023, O10311, O10312, O10313, O10319, O1032, O1033, O10411, O10412, O10413, O10419, O1042, O1043, O10911, O10912, O10913, O10919, O1092, O1093, O111, O112, O113, O114, O115, O119, O161, O162, O163, O164, O165, O169 |
| Hypothyrodism | E000, E001, E002, E009, E018, E02, E030, E031, E032, E033, E038, E039, E890 |
| Liver Disease | B180, B181, B182, I8500, I8501, I8510, I8511, K700, K702, K7030, K7031, K7040, K7041, K709, K7210, K7211, K7290, K7291, K730, K731, K732, K738, K739, K740, K741, K742, K743, K744, K745, K7460, K7469, K754, K7581, K760, K766, K7689, K769, Z944 |

© 2020 Asch DA et al. JAMA Internal Medicine.

| | |
|---|---|
| Lymphoma | C8100, C8101, C8102, C8103, C8104, C8105, C8106, C8107, C8108, C8109, C8110, C8111, C8112, C8113, C8114, C8115, C8116, C8117, C8118, C8119, C8120, C8121, C8122, C8123, C8124, C8125, C8126, C8127, C8128, C8129, C8130, C8131, C8132, C8133, C8134, C8135, C8136, C8137, C8138, C8139, C8140, C8141, C8142, C8143, C8144, C8145, C8146, C8147, C8148, C8149, C8170, C8171, C8172, C8173, C8174, C8175, C8176, C8177, C8178, C8179, C8190, C8191, C8192, C8193, C8194, C8195, C8196, C8197, C8198, C8199, C8200, C8201, C8202, C8203, C8204, C8205, C8206, C8207, C8208, C8209, C8210, C8211, C8212, C8213, C8214, C8215, C8216, C8217, C8218, C8219, C8220, C8221, C8222, C8223, C8224, C8225, C8226, C8227, C8228, C8229, C8230, C8231, C8232, C8233, C8234, C8235, C8236, C8237, C8238, C8239, C8240, C8241, C8242, C8243, C8244, C8245, C8246, C8247, C8248, C8249, C8250, C8251, C8252, C8253, C8254, C8255, C8256, C8257, C8258, C8259, C8260, C8261, C8262, C8263, C8264, C8265, C8266, C8267, C8268, C8269, C8280, C8281, C8282, C8283, C8284, C8285, C8286, C8287, C8288, C8289, C8290, C8291, C8292, C8293, C8294, C8295, C8296, C8297, C8298, C8299, C8300, C8301, C8302, C8303, C8304, C8305, C8306, C8307, C8308, C8309, C8310, C8311, C8312, C8313, C8314, C8315, C8316, C8317, C8318, C8319, C8330, C8331, C8332, C8333, C8334, C8335, C8336, C8337, C8338, C8339, C8350, C8351, C8352, C8353, C8354, C8355, C8356, C8357, C8358, C8359, C8370, C8371, C8372, C8373, C8374, C8375, C8376, C8377, C8378, C8379, C8380, C8381, C8382, C8383, C8384, C8385, C8386, C8387, C8388, C8389, C8390, C8391, C8392, C8393, C8394, C8395, C8396, C8397, C8398, C8399, C8400, C8401, C8402, C8403, C8404, C8405, C8406, C8407, C8408, C8409, C8410, C8411, C8412, C8413, C8414, C8415, C8416, C8417, C8418, C8419, C8440, C8441, C8442, C8443, C8444, C8445, C8446, C8447, C8448, C8449, C8460, C8461, C8462, C8463, C8464, C8465, C8466, C8467, C8468, C8469, C8470, C8471, C8472, C8473, C8474, C8475, C8476, C8477, C8478, C8479, C8490, C8491, C8492, C8493, C8494, C8495, C8496, C8497, C8498, C8499, C84A0, C84A1, C84A2, C84A3, C84A4, C84A5, C84A6, C84A7, C84A8, C84A9, C84Z0, C84Z1, C84Z2, C84Z3, C84Z4, C84Z5, C84Z6, C84Z7, C84Z8, C84Z9, C8510, C8511, C8512, C8513, C8514, C8515, C8516, C8517, C8518, C8519, C8520, C8521, C8522, C8523, C8524, C8525, C8526, C8527, C8528, C8529, C8580, C8581, C8582, C8583, C8584, C8585, C8586, C8587, C8588, C8589, C8590, C8591, C8592, C8593, C8594, C8595, C8596, C8597, C8598, C8599, C860, C861, C862, C863, C864, C865, C866, C880, C882, C883, C884, C888, C889, C9000, C9001, C9002, C9010, C9011, C9012, C9020, C9021, C9022, C9030, C9031, C9032, C960, C962, C9620, C9621, C9622, C9629, C964, C969, C96A, C96Z, D47Z9 |
| Fluid and Electrolyte Disorder | E860, E861, E869, E870, E871, E872, E873, E874, E875, E876, E8770, E8771, E8779, E878 |
| Metastatic Cancer | C770, C771, C772, C773, C774, C775, C778, C779, C7800, C7801, C7802, C781, C782, C7830, C7839, C784, C785, C786, C787, C7880, C7889, C7900, C7901, C7902, C7910, C7911, C7919, C792, C7931, C7932, C7940, C7949, C7951, C7952, C7960, C7961, C7962, C7970, C7971, C7972, C7981, C7982, C7989, C799, C7B00, C7B01, C7B02, C7B03, C7B04, C7B09, C7B1, C7B8, C800, C801, R180 |
| Neurological Disorder | E7500, E7501, E7502, E7509, E7510, E7511, E7519, E7523, E7525, E7526, E7529, E754, F842, G10, G110, G111, G112, G113, G114, G118, G119, G120, G121, G1220, G1221, G1222, G1223, G1224, G1225, G1229, G128, G129, G132, G138, G20, G214, G2401, G2402, G2409, G242, G248, G254, G255, G2581, G300, G301, G308, G309, G3101, G3109, G311, G312, G3181, G3182, G3183, G3184, G3185, G3189, G319, G3281, G35, G361, G368, G369, G370, G371, G372, G373, G374, G375, G378, G379, G40001, G40009, G40011, G40019, G40101, G40109, G40111, G40119, G40201, G40209, G40211, G40219, G40301, G40309, G40311, G40319, G40401, G40409, G40411, G40419, G40501, G40509, G40801, G40802, G40803, G40804, G40811, G40812, G40813, G40814, G40821, G40822, G40823, G40824, G4089, G40901, G40909, G40911, G40919, G40A01, G40A09, G40A11, G40A19, G40B01, G40B09, G40B11, G40B19, G47411, G47419, G47421, G47429, G803, G890, G910, G911, G912, G913, G914, G918, G919, G937, G9389, G939, G94, O99350, O99351, O99352, O99353, O99354, O99355, P9160, P9161, P9162, P9163, R410, R4182, R4701, R5600, R5601, R561, R569 |
| Obesity | E6601, E6609, E661, E662, E668, E669, O99210, O99211, O99212, O99213, O99214, O99215, R939, Z6830, Z6831, Z6832, Z6833, Z6834, Z6835, Z6836, Z6837, Z6838, Z6839, Z6841, Z6842, Z6843, Z6844, Z6845, Z6854 |

© 2020 Asch DA et al. JAMA Internal Medicine.

| | |
|---|---|
| Paralysis | G041, G800, G801, G802, G804, G808, G809, G8100, G8101, G8102, G8103, G8104, G8110, G8111, G8112, G8113, G8114, G8190, G8191, G8192, G8193, G8194, G8220, G8221, G8222, G8250, G8251, G8252, G8253, G8254, G830, G8310, G8311, G8312, G8313, G8314, G8320, G8321, G8322, G8323, G8324, G8330, G8331, G8332, G8333, G8334, G834, G835, G8381, G8382, G8383, G8384, G8389, G839, I69031, I69032, I69033, I69034, I69039, I69041, I69042, I69043, I69044, I69049, I69051, I69052, I69053, I69054, I69059, I69061, I69062, I69063, I69064, I69065, I69069, I69131, I69132, I69133, I69134, I69139, I69141, I69142, I69143, I69144, I69149, I69151, I69152, I69153, I69154, I69159, I69161, I69162, I69163, I69164, I69165, I69169, I69231, I69232, I69233, I69234, I69239, I69241, I69242, I69243, I69244, I69249, I69251, I69252, I69253, I69254, I69259, I69261, I69262, I69263, I69264, I69265, I69269, I69331, I69332, I69333, I69334, I69339, I69341, I69342, I69343, I69344, I69349, I69351, I69352, I69353, I69354, I69359, I69361, I69362, I69363, I69364, I69365, I69369, I69831, I69832, I69833, I69834, I69839, I69841, I69842, I69843, I69844, I69849, I69851, I69852, I69853, I69854, I69859, I69861, I69862, I69863, I69864, I69865, I69869, I69931, I69932, I69933, I69934, I69939, I69941, I69942, I69943, I69944, I69949, I69951, I69952, I69953, I69954, I69959, I69961, I69962, I69963, I69964, I69965, I69969, R532 |
| Peripheral Vascular Disease | I700, I701, I70201, I70202, I70203, I70208, I70209, I70211, I70212, I70213, I70218, I70219, I70221, I70222, I70223, I70228, I70229, I70231, I70232, I70233, I70234, I70235, I70238, I70239, I70241, I70242, I70243, I70244, I70245, I70248, I70249, I7025, I70261, I70262, I70263, I70268, I70269, I70291, I70292, I70293, I70298, I70299, I70301, I70302, I70303, I70308, I70309, I70311, I70312, I70313, I70318, I70319, I70321, I70322, I70323, I70328, I70329, I70331, I70332, I70333, I70334, I70335, I70338, I70339, I70341, I70342, I70343, I70344, I70345, I70348, I70349, I7035, I70361, I70362, I70363, I70368, I70369, I70391, I70392, I70393, I70398, I70399, I70401, I70402, I70403, I70408, I70409, I70411, I70412, I70413, I70418, I70419, I70421, I70422, I70423, I70428, I70429, I70431, I70432, I70433, I70434, I70435, I70438, I70439, I70441, I70442, I70443, I70444, I70445, I70448, I70449, I7045, I70461, I70462, I70463, I70468, I70469, I70491, I70492, I70493, I70498, I70499, I70501, I70502, I70503, I70508, I70509, I70511, I70512, I70513, I70518, I70519, I70521, I70522, I70523, I70528, I70529, I70531, I70532, I70533, I70534, I70535, I70538, I70539, I70541, I70542, I70543, I70544, I70545, I70548, I70549, I7055, I70561, I70562, I70563, I70568, I70569, I70591, I70592, I70593, I70598, I70599, I70601, I70602, I70603, I70608, I70609, I70611, I70612, I70613, I70618, I70619, I70621, I70622, I70623, I70628, I70629, I70631, I70632, I70633, I70634, I70635, I70638, I70639, I70641, I70642, I70643, I70644, I70645, I70648, I70649, I7065, I70661, I70662, I70663, I70668, I70669, I70691, I70692, I70693, I70698, I70699, I70701, I70702, I70703, I70708, I70709, I70711, I70712, I70713, I70718, I70719, I70721, I70722, I70723, I70728, I70729, I70731, I70732, I70733, I70734, I70735, I70738, I70739, I70741, I70742, I70743, I70744, I70745, I70748, I70749, I7075, I70761, I70762, I70763, I70768, I70769, I70791, I70792, I70793, I70798, I70799, I708, I7090, I7091, I7092, I7100, I7101, I7102, I7103, I711, I712, I713, I714, I715, I716, I718, I719, I720, I721, I722, I723, I724, I725, I726, I728, I729, I731, I7381, I7389, I739, I742, I743, I744, I76, I771, I7770, I7771, I7772, I7773, I7774, I7775, I7776, I7777, I7779, I790, I791, I798, K551, K558, K559, Z95820, Z95828 |
| Psychosis | F200, F201, F202, F203, F205, F2081, F2089, F209, F22, F23, F24, F250, F251, F258, F259, F28, F29, F3010, F3011, F3012, F3013, F302, F303, F304, F308, F309, F310, F3110, F3111, F3112, F3113, F312, F3130, F3131, F3132, F314, F315, F3160, F3161, F3162, F3163, F3164, F3170, F3171, F3172, F3173, F3174, F3175, F3176, F3177, F3178, F3181, F3189, F319, F324, F325, F3340, F3341, F3342, F348, F3481, F3489, F349, F39, F4489, F843 |
| Pulmonary Circulation | I2601, I2602, I2609, I2690, I2692, I2699, I270, I271, I2781, I2782, I2783, I2789, I279, I289, T800XXA, T82817A, T82818A |
| Chronic Kidney Disease | I120, I1311, I132, N183, N184, N185, N186, N189, N19, Z4901, Z4902, Z4931, Z4932, Z9115, Z940, Z992 |

© 2020 Asch DA et al. JAMA Internal Medicine.

| | |
|---|---|
| Solid Tumor without Metastasis | C000, C001, C002, C003, C004, C005, C006, C008, C009, C01, C020, C021, C022, C023, C024, C028, C029, C030, C031, C039, C040, C041, C048, C049, C050, C051, C052, C058, C059, C060, C061, C062, C0680, C0689, C069, C07, C080, C081, C089, C090, C091, C098, C099, C100, C101, C102, C103, C104, C108, C109, C110, C111, C112, C113, C118, C119, C12, C130, C131, C132, C138, C139, C140, C142, C148, C153, C154, C155, C158, C159, C160, C161, C162, C163, C164, C165, C166, C168, C169, C170, C171, C172, C173, C178, C179, C180, C181, C182, C183, C184, C185, C186, C187, C188, C189, C19, C20, C210, C211, C212, C218, C220, C221, C222, C223, C224, C227, C228, C229, C23, C240, C241, C248, C249, C250, C251, C252, C253, C254, C257, C258, C259, C260, C261, C269, C300, C301, C310, C311, C312, C313, C318, C319, C320, C321, C322, C323, C328, C329, C33, C3400, C3401, C3402, C3410, C3411, C3412, C342, C3430, C3431, C3432, C3480, C3481, C3482, C3490, C3491, C3492, C37, C380, C381, C382, C383, C384, C388, C390, C399, C4000, C4001, C4002, C4010, C4011, C4012, C4020, C4021, C4022, C4030, C4031, C4032, C4080, C4081, C4082, C4090, C4091, C4092, C410, C411, C412, C413, C414, C419, C430, C4310, C4311, C43111, C43112, C4312, C43121, C43122, C4320, C4321, C4322, C4330, C4331, C4339, C434, C4351, C4352, C4359, C4360, C4361, C4362, C4370, C4371, C4372, C438, C439, C450, C451, C452, C457, C470, C4710, C4711, C4712, C4720, C4721, C4722, C473, C474, C475, C476, C478, C479, C480, C481, C482, C488, C490, C4910, C4911, C4912, C4920, C4921, C4922, C493, C494, C495, C496, C498, C499, C49A0, C49A1, C49A2, C49A3, C49A4, C49A5, C49A9, C4A0, C4A10, C4A11, C4A111, C4A112, C4A12, C4A121, C4A122, C4A20, C4A21, C4A22, C4A30, C4A31, C4A39, C4A4, C4A51, C4A52, C4A59, C4A60, C4A61, C4A62, C4A70, C4A71, C4A72, C4A8, C4A9, C50011, C50012, C50019, C50021, C50022, C50029, C50111, C50112, C50119, C50121, C50122, C50129, C50211, C50212, C50219, C50221, C50222, C50229, C50311, C50312, C50319, C50321, C50322, C50329, C50411, C50412, C50419, C50421, C50422, C50429, C50511, C50512, C50519, C50521, C50522, C50529, C50611, C50612, C50619, C50621, C50622, C50629, C50811, C50812, C50819, C50821, C50822, C50829, C50911, C50912, C50919, C50921, C50922, C50929, C510, C511, C512, C518, C519, C52, C530, C531, C538, C539, C540, C541, C542, C543, C548, C549, C55, C561, C562, C569, C5700, C5701, C5702, C5710, C5711, C5712, C5720, C5721, C5722, C573, C574, C577, C578, C579, C58, C600, C601, C602, C608, C609, C61, C6200, C6201, C6202, C6210, C6211, C6212, C6290, C6291, C6292, C6300, C6301, C6302, C6310, C6311, C6312, C632, C637, C638, C639, C641, C642, C649, C651, C652, C659, C661, C662, C669, C670, C671, C672, C673, C674, C675, C676, C677, C678, C679, C680, C681, C688, C689, C6900, C6901, C6902, C6910, C6911, C6912, C6920, C6921, C6922, C6930, C6931, C6932, C6940, C6941, C6942, C6950, C6951, C6952, C6960, C6961, C6962, C6980, C6981, C6982, C6990, C6991, C6992, C700, C701, C709, C710, C711, C712, C713, C714, C715, C716, C717, C718, C719, C720, C721, C7220, C7221, C7222, C7230, C7231, C7232, C7240, C7241, C7242, C7250, C7259, C729, C73, C7400, C7401, C7402, C7410, C7411, C7412, C7490, C7491, C7492, C750, C751, C752, C753, C754, C755, C758, C759, C760, C761, C762, C763, C7640, C7641, C7642, C7650, C7651, C7652, C768, C7A00, C7A010, C7A011, C7A012, C7A019, C7A020, C7A021, C7A022, C7A023, C7A024, C7A025, C7A026, C7A029, C7A090, C7A091, C7A092, C7A093, C7A094, C7A095, C7A096, C7A098, D030, D0310, D0311, D03111, D03112, D0312, D03121, D03122, D0320, D0321, D0322, D0330, D0339, D034, D0351, D0352, D0359, D0360, D0361, D0362, D0370, D0371, D0372, D038, D039, E3121, E3122, E3123 |
| Peptic Ulcer | K254, K255, K256, K257, K259, K264, K265, K266, K267, K269, K274, K275, K276, K277, K279, K284, K285, K286, K287, K289 |
| Valvular Disorder | A5203, I050, I051, I052, I058, I059, I060, I061, I062, I068, I069, I070, I071, I072, I078, I079, I080, I081, I082, I083, I088, I089, I091, I0989, I340, I341, I342, I348, I349, I350, I351, I352, I358, I359, I360, I361, I362, I368, I369, I370, I371, I372, I378, I379, I38, I39, Q230, Q231, Q232, Q233, Z952, Z953, Z954 |
| Weight Loss | E40, E41, E42, E43, E440, E441, E45, E46, E640, R634, R636 |

© 2020 Asch DA et al. JAMA Internal Medicine.

References for Appendix


1. George EI, Ročková V, Rosenbaum PR, Satopää VA, Silber JH . Mortality rate estimation and standardization for public reporting: medicare's hospital compare, Journal of the American Statistical Association. 2017;112:519, 933-947. DOI: 10.1080/01621459.2016.1276021
2. Silber JH, Rosenbaum PR, Brachet TJ, et al. The Hospital Compare mortality model and the volume-outcome relationship. Health Serv Res. 2010;45(5 Pt 1):1148-1167. doi:10.1111/j.1475-6773.2010.01130.x
3. Silber JH, Satopää VA, Mukherjee N, et al. Improving Medicare's Hospital Compare Mortality Model. Health Serv Res. 2016;51 Suppl 2(Suppl 2):1229-1247. doi:10.1111/1475-6773.12478
4. Silber JH, Rosenbaum PR, Niknam BA, et al. Comparing Outcomes and Costs of Surgical Patients Treated at Major Teaching and Nonteaching Hospitals: A National Matched Analysis. *Ann Surg*. 2020;271(3):412-421. doi:10.1097/SLA.0000000000003602
5. Drye EE, Normand SL, Wang Y, et al. Comparison of hospital risk-standardized mortality rates calculated by using in-hospital and 30-day models: an observational study with implications for hospital profiling [published correction appears in Ann Intern Med. 2012 Mar 6;156(5):404]. *Ann Intern Med*. 2012;156(1 Pt 1):19-26. doi:10.7326/0003-4819-156-1-201201030-00004
6. Normand S-LT, Shahian DM. Statistical and clinical aspects of hospital outcomes profiling. Stat Sci. 2007;22:206-226. doi:10.1214/088342307000000096
7. Self SG, Liang K-Y. Asymptotic properties of maximum likelihood estimators and likelihood ratio yests under nonstandard conditions. J Am Stat Assoc. 1987;83(398):605-10.
8. Stram DO, Lee JW. Variance components testing in the longitudinal mixed effects model. *Biometrics*. 1994;50(4):1171-1177. [published correction appears in Biometrics 1995 Sep;51(3):1196]


eMethods.

An illustration of estimating risk standardized event rates to measure variation in hospital outcomes based on simulated data

Md Nazmul Islam

11/16/2020

Necessary functions

We load the necessary packages and define the inputs and outputs associated with the main functions

used in the illustration. Next we spell out the analytical functions used in the manuscript;

we define them in three sections - (A) data arrangment, (B) model fitting, and (C) visualization respectively.

```{r}

# R version 3.6.3

library(MuMIn); library(glmmTMB); library(MASS); library(dplyr); library(lme4);

library(DescTools); library(psych); library(GLMMadaptive); library(performance); library(fastDummies)


## Definitions (input)

# ylist = Response variable name; "mbr_death_flag"
```

© 2020 Asch DA et al. JAMA Internal Medicine.

# XXX_adj = vector of all covariates; c("age", "gender", "cancer",...)

# ranlist = Random effect; "(`1 | hosp_name)"

# reml = TRUE; Boolean for REML, otherwise ML

# Dataf_original = Original unarranged raw data (Variable names should be exactly as same as defined within this function)

# Dataf = Dataframe containing reponse & covariates after arranging in appropriate category

# Dataf_el = Dataframe containing response and covariates for early and late period with at-least 10 patients in each period

# Data_score = Dataframe with scores having hospital names

# Data_score_earlylate = Dataframe with scores having hospital names with period specifying early and late time

# Data_analytical = Analytical dataframe used in computing standardized scores

# hosp_id = Hospital attributes name (e.g., bed_size; see below for example)

# apnd2 = Extra hospital data (as needed) to add in the dataframe

# comorb_nm = Comorbidity names in details (see below for example)

# addsize = TRUE; Boolean whether size is added in computing scores in hierarchical model

# laplace = TRUE; Boolean if TRUE laplace approximation is adopted; otherwise

#               approximation of integrand is considered via Gaussian quadrature

# AA/BB/CC/DD/EE/FF  = Different filtering options to extract data from the Dataf_original

# s1 = Difference in scores used in Bland-Altman (BA) plot

# s2 = Average of scores used in BA plot

# score_early2j, score_later2j = Scores for early and late period for each site used in BA plot

## Definitions (Output)

# fom3 = glmm object

# hospital_fit_object = Hospital model fit

# LRT_re = LRT of random effects

# aic_null = Akaike information criteria for GLM model

# aic_alt = Akaike information criteria for GLMM model

# SS = Marginal & conditional R-squared (goodness of fit) for GLMM

# Summary = Summary of GLMM fit

# cor_lme_lmer = Correlation of fixed effects between GLM and GLMM

© 2020 Asch DA et al. JAMA Internal Medicine.

# rank_cor = Correlation between predicted probabilities and observed responses (C and Dxy statistic) for GLMM

# nY = Response

# ybar = Observed death rate

# orp = Coefficients estimates (fixed effects) for GLMM

# RANFOM = BLUPs

# or = Odds ratio based on GLMM

# lb = Lower bound of 95% CI for OR

# ub = Upper bound of 95% CI for OR

# pred = Predicted probabilities for Dyre et al. (Numerator)

# estm = Expected probabilities for Dyre et al. (Denominator)

# uid = Site names

# score = Score based on Dyre et al.

# Score_DS_mean = Score based on Silber et al.

# Score_DS = N X K matrix of scores (each patients (total N) probability for each one of the K hospitals)

# unadj_score =  Observed (raw) rate for each hospital

# hosp_orig_vol = Volume for each hospital

## Type of functions used

# A1. arrange_dataf_race()

# A2. arrange_dataf_hospital

# A3. '%!in%'

# A4. filtering_function()

# A5. firstup()

# A6. function_character()

# B1. glmmfit()

# B2. glmmfit_intslope()

# B3. hospital_outcome_regression()

# B4. qunitile_compare()

# B5. glmmfit_patient_hospital()

# C1. plotOR_paper()

# C2. plotScore_paper_single_rotate()

© 2020 Asch DA et al. JAMA Internal Medicine.

```
# C3. bland_altman_all()
# C4. summary_statistics_table()
# C5. Compare_numeric_boxplot_ggplot()
# C6. plotOR_paper_hospital()
# C7. quintile_descriptive()


# A1. Create categorical Dataf_original (Multiple rows per site)
arrange_dataf_race <- function(Dataf_original){



  Dataf_original$academic_flag <- ifelse(Dataf_original$academic_flag == 1, "Academic",
                               ifelse(is.na(Dataf_original$academic_flag) == TRUE, NA, "Non-academic"))

  Dataf_original$non_profit_flag <- ifelse(Dataf_original$non_profit_flag == 1, "Non-profit",
                                ifelse(is.na(Dataf_original$non_profit_flag) == TRUE, NA, "Profit"))



  Dataf_original$icu_beds_class <- ifelse(Dataf_original$icu_beds < 10, "0 <= ICU < 10",
                               ifelse(Dataf_original$icu_beds >= 10 & Dataf_original$icu_beds < 20, "10 <= ICU < 20",
                                   ifelse(Dataf_original$icu_beds >= 20 & Dataf_original$icu_beds < 30, "20 <= ICU < 30",
                                       ifelse(Dataf_original$icu_beds >= 30 & Dataf_original$icu_beds < 40, "30 <= ICU < 40",
                                           ifelse(Dataf_original$icu_beds >= 40 & Dataf_original$icu_beds < 50, "40 <= ICU < 50",
                                               ifelse(Dataf_original$icu_beds >= 50 & Dataf_original$icu_beds < 70, "50 <= ICU < 70",
                                                   ifelse(is.na(Dataf_original$icu_beds) == TRUE, NA, "70 <= ICU")))))))


  table(Dataf_original$icu_beds_class)


  summary(Dataf_original$resident_to_bed_ratio)
  Dataf_original$resident_to_bed_ratio_class <- ifelse(Dataf_original$resident_to_bed_ratio < 0.001, "0 <= Ratio <
0.001",
```

© 2020 Asch DA et al. JAMA Internal Medicine.

```
                              ifelse(Dataf_original$resident_to_bed_ratio >= 0.001 & Dataf_original$resident_to_bed_ratio <
0.01, "0.001 <= Ratio < 0.01",
                                  ifelse(Dataf_original$resident_to_bed_ratio >= 0.01 &
Dataf_original$resident_to_bed_ratio < 0.05, "0.01 <= Ratio < 0.05",
                                      ifelse(Dataf_original$resident_to_bed_ratio >= 0.05 &
Dataf_original$resident_to_bed_ratio < 0.10, "0.05 <= Ratio < 0.10",
                                          ifelse(Dataf_original$resident_to_bed_ratio >= 0.10 &
Dataf_original$resident_to_bed_ratio < 0.15, "0.10 <= Ratio < 0.15",
                                              ifelse(Dataf_original$resident_to_bed_ratio >= 0.15 &
Dataf_original$resident_to_bed_ratio < 0.25, "0.15 <= Ratio < 0.25",
                                                  ifelse(Dataf_original$resident_to_bed_ratio >= 0.25 &
Dataf_original$resident_to_bed_ratio < 0.50, "0.25 <= Ratio < 0.50",
                                                      ifelse(is.na(Dataf_original$resident_to_bed_ratio) == TRUE, NA, "0.50
<= Ratio"))))))))
 table(Dataf_original$resident_to_bed_ratio_class)




 summary(Dataf_original$beds)
 Dataf_original$beds_class <- ifelse(Dataf_original$beds < 200, "0 <= Beds < 200",
               ifelse(Dataf_original$beds >= 200 & Dataf_original$beds < 400, "200 <= Beds < 400",
                   ifelse(Dataf_original$beds >= 400 & Dataf_original$beds < 600, "400 <= Beds < 600",
                       ifelse(is.na(Dataf_original$beds) == TRUE, NA, "600 <= Beds"))))

 table(Dataf_original$beds_class)

 ## Counts of days since Feb1
 count_day <- as.numeric(c(Dataf_original$mbr_admit_dt - as.Date("2020-01-01"))) # year-month-day
 count_day_class <- ifelse(count_day <= 90, "1 < Count <= 90",
               ifelse(count_day > 90 & count_day <= 120, "90 < Count <= 120",
                   ifelse(count_day > 120 & count_day <= 150, "120 < Count <= 150",
                       "150 < Count")))
```

© 2020 Asch DA et al. JAMA Internal Medicine.

```
Dataf_original$count_day  <- count_day

Dataf_original$count_day2 <- count_day^2


Dataf_original$count_day_class <- factor(count_day_class, levels = c("1 < Count <= 90", "90 < Count <= 120",
                                     "120 < Count <= 150", "150 < Count"))


#table(Dataf_original$count_day_class)

#table(Dataf_original$count_day_class, Dataf_original$mbr_death_flag)

#tb[, 2]/ rowSums(tb)


Dataf_original$gender <- ifelse(Dataf_original$gender == "m", "Male",
              ifelse(Dataf_original$gender == "f", "Female", NA))


ixf <- grep("aeci_", colnames(Dataf_original),  value  = F)


QQ <- paste0("Dataf_original$", colnames(Dataf_original)[ixf]," <- Dataf_original %>% select(",
colnames(Dataf_original)[ixf],") %>%
        replace(is.na(.), 0) %>%
        mutate(", colnames(Dataf_original)[ixf]," = ifelse(", colnames(Dataf_original)[ixf]," > 0, 'Yes', 'No')) %>%
        unlist()",
        collapse = ";")
QQ <- eval(parse(text = QQ))


comorb_id <- grep("aeci_", colnames(Dataf_original),  value  = T)
```

© 2020 Asch DA et al. JAMA Internal Medicine.

```
Dataf_original$mapd_comm_flag <- ifelse(Dataf_original$lob == "medicare", "Medicare", "Commercial")


# age class

Dataf_original$age_class <- ifelse(Dataf_original$age <= 45, "18 < age <= 45",
                    #ifelse(Dataf_original$age > 35 & Dataf_original$age <= 45, "35 < age <= 45",
                    ifelse(Dataf_original$age > 45 & Dataf_original$age <= 55, "45 < age <= 55",
                         ifelse(Dataf_original$age > 55 & Dataf_original$age <= 65, "55 < age <= 65",
                              ifelse(Dataf_original$age > 65 & Dataf_original$age <= 75, "65 < age <= 75",
                                   ifelse(Dataf_original$age > 75 & Dataf_original$age <= 85, "75 < age <= 85",

                                        "85 < age")))))




Dataf_original$elix <- ifelse(Dataf_original$elix < 0, 0, Dataf_original$elix); summary(Dataf_original$elix)

Dataf_original$elix[which(is.na(Dataf_original$elix) == TRUE)] <- 0


Dataf_original$elix_class <- ifelse(Dataf_original$elix >= 0 & Dataf_original$elix < 1, "[0, 1)",
                    ifelse(Dataf_original$elix >= 1 & Dataf_original$elix < 10, "[1, 10)",
                         ifelse(Dataf_original$elix >= 10 & Dataf_original$elix < 20, "[10, 20)",
                              ">=20")))



Dataf_original$race_cd[which(is.na(Dataf_original$race_cd) == TRUE | Dataf_original$race_cd %in% c("hispanic",
"native american",
                                                  "other", "asian"))] <- "unknown"


Dataf_original$race_cd_class <- ifelse(Dataf_original$race_cd %!in% c("african-american", "caucasian"),
"other/unknown",
                      Dataf_original$race_cd)
```

© 2020 Asch DA et al. JAMA Internal Medicine.

```
Dataf_original$mbr_death_flag <- ifelse(Dataf_original$mbr_death_flag == 1, "Event", "Alive")
Dataf_original$mbr_death_flag  <- Dataf_original$mbr_death_flag %>% replace(is.na(.), "Alive")


Dataf_original$mbr_mortality_flag <- ifelse(Dataf_original$mbr_mortality_flag == 1, "Event", "Alive")
Dataf_original$mbr_mortality_flag  <- Dataf_original$mbr_mortality_flag %>% replace(is.na(.), "Alive")


Dataf_original$snf_nursing_flag <- ifelse(Dataf_original$snf_nursing_flag == 1, "Yes", "No")
Dataf_original$snf_nursing_flag  <- Dataf_original$snf_nursing_flag %>% replace(is.na(.), "No")

Dataf_original$hosp_region <- ifelse(Dataf_original$hosp_region == "ct", "Central",
                         ifelse(Dataf_original$hosp_region == "ne", "North-east",
                           ifelse(Dataf_original$hosp_region == "se", "South-east",
                             ifelse(Dataf_original$hosp_region == "we", "West", "Other"))))


Dataf_original$hosp_usr_class <- ifelse(Dataf_original$hosp_usr_class == "U", "Urban",
                          ifelse(Dataf_original$hosp_usr_class == "R", "Rural",
                            ifelse(Dataf_original$hosp_usr_class == "S", "Semi-urban",
                              "Other")))


Dataf_original$hosp_income_class <- ifelse(Dataf_original$hosp_avg_income <= 50000, "50K >= income",
                           ifelse(Dataf_original$hosp_avg_income > 50000 & Dataf_original$hosp_avg_income <= 65000, "50K
< income <= 65K",
                             ifelse(Dataf_original$hosp_avg_income > 65000 & Dataf_original$hosp_avg_income <= 80000,
"65K < income <= 80K",
                               "80K < income")))
```

© 2020 Asch DA et al. JAMA Internal Medicine.

```
  Dataf_original$mbr_income_class <- ifelse(Dataf_original$mbr_income <= 50000, "50K >= income", #"60K < income")
                              ifelse(Dataf_original$mbr_income > 50000 & Dataf_original$mbr_income <= 65000, "50K < income
<= 65K",
                                   ifelse(Dataf_original$mbr_income > 65000 & Dataf_original$mbr_income <= 100000, "65K <
income <= 100K",
                                        "100K < income")))



  output = list(Dataf_original = Dataf_original, comorb_id = comorb_id)
}



# A2. Create data for hospital regression and turn one row per site data
arrange_dataf_hospital <- function(Data_score, Data_analytical, hosp_id, apnd2,
                              period = FALSE, allsite = "Yes-Median", name2add, name2add_num){

  symbol <- "\u2265"; symbol2 <- intToUtf8(8804)
  mid <- (Data_score$reference)
  Data7 <- do.call(rbind, lapply(seq_len(length(mid)), function(uu)
   Data_analytical[which(Data_analytical$hosp_name == mid[uu])[1], # same for all subjects
        colnames(Data_analytical) %in% c(hosp_id, "resident_to_bed_ratio","hosp_name", "icu_beds", "beds")]
  ))

  ## Redefining ICU
  mt <- match(Data7$hosp_name, apnd2$hosp_name)
  sum(is.na(mt))
  Data7$icu_beds <- apnd2$ICU_beds[mt]



  ## Redefining profit
```

© 2020 Asch DA et al. JAMA Internal Medicine.

```
Data7$non_profit_flag <- apnd2$Profit_flag[mt]
Data7$non_profit_flag <- ifelse(Data7$non_profit_flag == "For Profit", "Profit",
                        ifelse(Data7$non_profit_flag == "Not for profit", "Non-profit", "Other"))
table(Data7$non_profit_flag)

## Cases
Data7$cases_per_10k <- apnd2$cases_per_10k[mt]
Data7$`Early.Period.1/1-4/30` <- apnd2$`Early.Period.1/1-4/30`[mt]
Data7$`Late.Period:.5/1-6/30` <- apnd2$`Late.Period:.5/1-6/30`[mt]
Data7$`%.change` <- apnd2$`%.change`[mt]



## Deaths
Data7$deaths_per_10k <- apnd2$deaths_per_10k[mt]

## Redefining user class
Data7$hosp_usr_class <- apnd2$Rural_urban[mt]
Data7$hosp_usr_class <- ifelse(Data7$hosp_usr_class == "R", "Rural", "Urban")






if(period == FALSE){
  Data7$score <- unlist(lapply(seq_len(length(mid)), function(uu)
    Data_score$score[which(Data_score$reference == mid[uu])]))

  Data7$unadj_score <- unlist(lapply(seq_len(length(mid)), function(uu)
    Data_score$unadjusted_score[which(Data_score$reference == mid[uu])]))
```

© 2020 Asch DA et al. JAMA Internal Medicine.

```
  if(allsite == "Yes-Median") {
    grp1 <- which(Data7$score <= median(Data7$score))
    Data7$event <- rep("Worse", nrow(Data7))
    Data7$event[grp1] <- "Better"
  }

} else if(period == "Early"){

  Data7$score <- unlist(lapply(seq_len(length(mid)), function(uu)
    Data_score$early_score[which(Data_score$reference == mid[uu])]))

  Data7$unadj_score <- unlist(lapply(seq_len(length(mid)), function(uu)
    Data_score$early_unadj_score[which(Data_score$reference == mid[uu])]))

} else if(period == "Later"){

  Data7$score <- unlist(lapply(seq_len(length(mid)), function(uu)
    Data_score$later_score[which(Data_score$reference == mid[uu])]))

  Data7$unadj_score <- unlist(lapply(seq_len(length(mid)), function(uu)
    Data_score$later_unadj_score[which(Data_score$reference == mid[uu])]))
} else if(period == "Both"){
  ## Defining response %increment
  Data7$early_score <- unlist(lapply(seq_len(length(mid)), function(uu)
    Data_score$early_score[which(Data_score$reference == mid[uu])]))

  Data7$later_score <- unlist(lapply(seq_len(length(mid)), function(uu)
    Data_score$later_score[which(Data_score$reference == mid[uu])]))

  incr <- (Data7$later_score - Data7$early_score) / Data7$early_score * 100
  grp1 <- which(incr <= -50)
```

© 2020 Asch DA et al. JAMA Internal Medicine.

```
  length(grp1)

  Data7$event <- rep("Worse", nrow(Data7))
  Data7$event[grp1] <- "Better"
  table(Data7$event)

  Data7$difference <- Data7$later_score - Data7$early_score
}

dim(Data7)


# icu
Data7$icu_beds_class <- ifelse(Data7$icu_beds < 20, "0 <= ICU < 20",
                        ifelse(Data7$icu_beds >= 20 & Data7$icu_beds < 60, "20 <= ICU < 60",
                            #ifelse(Data7$icu_beds >= 60 & Data7$icu_beds < 100, "60 <= ICU < 100",
                            # ifelse(Data7$icu_beds >= 90 & Data7$icu_beds < 120, "90 <= ICU < 120",
                            ifelse(is.na(Data7$icu_beds) == TRUE, NA, "60 <= ICU")))
table(Data7$icu_beds_class); sum(is.na(Data7$icu_beds_class))
t1 <- table(Data7$icu_beds_class, Data7$event)
t1[, 2] / rowSums(t1)

# beds
Data7$beds_class <- ifelse(Data7$beds < 150, "0 <= Beds < 150",
                      ifelse(Data7$beds >= 150 & Data7$beds < 300, "150 <= Beds < 300",
                          ifelse(Data7$beds >= 300 & Data7$beds < 450, "300 <= Beds < 450",
                              #ifelse(Data7$beds >= 450 & Data7$beds < 600, "450 <= Beds < 600",
                              ifelse(is.na(Data7$beds) == TRUE, NA, "450 <= Beds"))))
table(Data7$beds_class); sum(is.na(Data7$beds_class))
t1 <- table(Data7$beds_class, Data7$event)
t1[, 2] / rowSums(t1)
```

© 2020 Asch DA et al. JAMA Internal Medicine.

```
# ratio
Data7$resident_to_bed_ratio_class <- ifelse(Data7$resident_to_bed_ratio < 0.05, "0 <= Ratio < 0.05",
                                     ifelse(Data7$resident_to_bed_ratio >= 0.05 & Data7$resident_to_bed_ratio < 0.25, "0.05 <=
Ratio < 0.25",
                                       ifelse(is.na(Data7$resident_to_bed_ratio) == TRUE, NA, "0.25 <= Ratio")))
table(Data7$resident_to_bed_ratio_class); sum(is.na(Data7$resident_to_bed_ratio_class))

table(Data7$hosp_usr_class)

# cases
summary(Data7$cases_per_10k)
summary(Data7$deaths_per_10k)

Data7$cases_class <- ifelse(Data7$cases_per_10k < 20, "0 <= Cases < 20",
                      ifelse(Data7$cases_per_10k >= 20 & Data7$cases_per_10k < 40, "20 <= Cases < 40",
                        ifelse(is.na(Data7$cases_per_10k) == TRUE, NA, "40 <= Cases")))

Data7$cases_class_early <- ifelse(Data7$`Early.Period.1/1-4/30` < 20, "0 <= Cases < 20",
                            ifelse(Data7$`Early.Period.1/1-4/30` >= 20 & Data7$`Early.Period.1/1-4/30` < 40, "20 <= Cases < 40",

                              ifelse(is.na(Data7$`Early.Period.1/1-4/30`) == TRUE, NA, "40 <= Cases")))

Data7$cases_class_late <- ifelse(Data7$`Late.Period:.5/1-6/30` < 20, "0 <= Cases < 20",
                           ifelse(Data7$`Late.Period:.5/1-6/30` >= 20 & Data7$`Late.Period:.5/1-6/30` < 40, "20 <= Cases < 40",

                             ifelse(is.na(Data7$`Late.Period:.5/1-6/30`) == TRUE, NA, "40 <= Cases")))


Data7$cases_class_late_diff <- ifelse(Data7$`Late.Period:.5/1-6/30` < 30, "0 <= Cases < 30",
```

© 2020 Asch DA et al. JAMA Internal Medicine.

```
ifelse(Data7$`Late.Period:.5/1-6/30` >= 30 & Data7$`Late.Period:.5/1-6/30` < 60, "30 <= Cases <
60",
        ifelse(Data7$`Late.Period:.5/1-6/30` >= 60 & Data7$`Late.Period:.5/1-6/30` < 90, "60 <= Cases
< 90",
            ifelse(is.na(Data7$`Late.Period:.5/1-6/30`) == TRUE, NA, "90 <= Cases"))))


Data7$cases_change_binary <- ifelse(Data7$`%.change` < 0, "Decrease",
                       ifelse(is.na(Data7$`%.change`) == TRUE, NA, "Increase"))


Data7$cases_interaction <- ifelse(Data7$cases_change_binary == "Increase" & Data7$cases_class_early == "20 <=
Cases < 40", "20 <= Cases < 40 & Increase",
                      ifelse(Data7$cases_change_binary == "Increase" & Data7$cases_class_early == "40 <= Cases", "40
<= Cases & Increase", "otherwise"))


Data7$`%.change` <- Data7$`%.change` * 100

Data7$cases_change <- ifelse(Data7$`%.change` < 0, "<0%",
                 ifelse(Data7$`%.change` >= 0 & Data7$`%.change` < 100, "[0,100)%",
                     ifelse(Data7$`%.change` >= 100 & Data7$`%.change` < 250, "[100,250)%",
                         ifelse(is.na(Data7$`%.change`) == TRUE, NA, ">=250%"))))


table(Data7$cases_class_late); sum(is.na(Data7$cases_class))

table(Data7$cases_change)

t1 <- table(Data7$cases_class, Data7$event)

t1[, 2] / rowSums(t1)


# deaths
```

© 2020 Asch DA et al. JAMA Internal Medicine.

```
Data7$deaths_class <- ifelse(Data7$deaths_per_10k < 1, "0 <= Deaths < 1",
                        ifelse(Data7$deaths_per_10k >= 1 & Data7$deaths_per_10k < 3, "1 <= Deaths < 3",
                          ifelse(Data7$deaths_per_10k >= 3 & Data7$deaths_per_10k < 5, "3 <= Deaths < 5",
                            ifelse(is.na(Data7$deaths_per_10k) == TRUE, NA, "5 <= Deaths"))))
table(Data7$deaths_class); sum(is.na(Data7$deaths_class))
t1 <- table(Data7$deaths_class, Data7$event)
t1[, 2] / rowSums(t1)



# Combination
Data7$combination1_flag <- ifelse(Data7$resident_to_bed_ratio <= 0.25 & Data7$academic_flag == "Academic",
                          "Academic|<=0.25", ifelse(Data7$resident_to_bed_ratio > 0.25 & Data7$academic_flag ==
"Academic",
                                    "Academic|>0.25", "Non"))


Data7$combination2_flag <- ifelse(Data7$resident_to_bed_ratio <= 0.25 & Data7$academic_flag == "Academic",
"Academic|<=0.25",
                          ifelse(Data7$resident_to_bed_ratio > 0.25 & Data7$academic_flag == "Academic",
"Academic|>0.25",
                            ifelse(Data7$resident_to_bed_ratio <= 0.25 & Data7$academic_flag == "Non-academic",
"Non|<=0.25",
                              ifelse(Data7$resident_to_bed_ratio > 0.25 & Data7$academic_flag == "Non-academic",
"Non|>0.25", NA))))


table(Data7$combination2_flag)




Data7 <- Data7 %>% mutate_if(is.character, as.factor)
Data7 <- within(Data7, event <- relevel(event, ref = "Better"))
Data7 <- within(Data7, icu_beds_class <- relevel(icu_beds_class, ref = "0 <= ICU < 20"))
Data7 <- within(Data7, resident_to_bed_ratio_class <- relevel(resident_to_bed_ratio_class, ref = "0 <= Ratio < 0.05"))
Data7 <- within(Data7, beds_class <- relevel(beds_class, ref = "0 <= Beds < 150"))
Data7 <- within(Data7, academic_flag <- relevel(academic_flag, ref = "Academic"))
```

© 2020 Asch DA et al. JAMA Internal Medicine.

```
Data7 <- within(Data7, hosp_usr_class  <- relevel(hosp_usr_class, ref = "Rural"))

Data7 <- within(Data7, non_profit_flag  <- relevel(non_profit_flag, ref = "Profit"))

Data7 <- within(Data7, hosp_region  <- relevel(hosp_region, ref = "Central"))

Data7 <- within(Data7, deaths_class  <- relevel(deaths_class, ref = "0 <= Deaths < 1"))

Data7 <- within(Data7, cases_class  <- relevel(cases_class, ref = "0 <= Cases < 20"))

Data7 <- within(Data7, cases_class_early  <- relevel(cases_class_early, ref = "0 <= Cases < 20"))

Data7 <- within(Data7, cases_class_late  <- relevel(cases_class_late, ref = "0 <= Cases < 20"))

Data7 <- within(Data7, cases_class_late_diff  <- relevel(cases_class_late_diff, ref = "0 <= Cases < 30"))

Data7 <- within(Data7, cases_change  <- relevel(cases_change, ref = "<0%"))

Data7 <- within(Data7, cases_interaction  <- relevel(cases_interaction, ref = "otherwise"))

Data7 <- within(Data7, cases_change_binary  <- relevel(cases_change_binary, ref = "Decrease"))

Data7 <- within(Data7, combination1_flag  <- relevel(combination1_flag, ref = "Non"))

Data7 <- within(Data7, combination2_flag  <- relevel(combination2_flag, ref = "Non|>0.25"))




## Hospital levels variables

# user

mlist_user <- c("hosp_usr_class"); varlist_user <- c("Location::Urban");

reflist_user <- rep("Rural", length(varlist_user)); mlist0_user <- rep("hosp_usr_class", length(varlist_user))


# ratio

mlist_ratio <- c("resident_to_bed_ratio_class"); varlist_ratio <- c("Resident to bed ratio::[0.05,0.25)", paste0("Resident
to bed ratio::", symbol,"0.25"));

reflist_ratio <- rep("[0,0.05)", length(varlist_ratio)); mlist0_ratio <- rep("resident_to_bed_ratio_class",
length(varlist_ratio))


# ICU

mlist_icu <- c("icu_beds_class"); varlist_icu <- c("Number of ICU beds::[20,60)",

                                   #"Number of ICU beds::[60,100)",
```

© 2020 Asch DA et al. JAMA Internal Medicine.

```
                                   paste0("Number of ICU beds::", symbol,"60"));
 reflist_icu <- rep("[0,20)", length(varlist_icu)); mlist0_icu <- rep("icu_beds_class", length(varlist_icu))



 # Combination1
 mlist_combination1 <- c("combination1_flag"); varlist_combination1 <- c(paste0("Academic|",symbol2,"0.25",
"Academic|>0.25"));
 reflist_combination1 <- rep("Non", length(varlist_combination1));
 mlist0_combination1 <- rep("combination1_flag", length(varlist_combination1))


 # Combination2
 mlist_combination2 <- c("combination2_flag"); varlist_combination2 <- c(paste0("Academic|",symbol2,"0.25"),
                                                 "Academic|>0.25", paste0("Non|",symbol2,"0.25"));
 reflist_combination2 <- rep("Non|>0.25", length(varlist_combination2));
 mlist0_combination2 <- rep("combination2_flag", length(varlist_combination2))




 # region
 mlist_region <- c("hosp_region"); varlist_region <- c("Region::North-east",
                                    "Region::South-east",
                                    "Region::West");
 reflist_region <- rep("Central", length(varlist_region)); mlist0_region <- rep("hosp_region", length(varlist_region))


 # beds
 mlist_beds <- c("beds_class"); varlist_beds <- c("Number of beds::[150,300)",
                                "Number of beds::[300,450)",
                                paste0("Number of beds::", symbol,"450"));
 reflist_beds <- rep("[0,150)", length(varlist_beds)); mlist0_beds <- rep("beds_class", length(varlist_beds))


 # academic
```

© 2020 Asch DA et al. JAMA Internal Medicine.

```
  mlist_academic <- c("academic_flag"); varlist_academic <- c("Hospital affiliation::Non-academic");
  reflist_academic <- rep("Academic", length(varlist_academic)); mlist0_academic <- rep("academic_flag",
length(varlist_academic))

  # profit
  mlist_profit <- c("non_profit_flag"); varlist_profit <- c("Profit status::Non-profit", "Profit status::Other");
  reflist_profit <- rep("For profit", length(varlist_profit)); mlist0_profit <- rep("non_profit_flag", length(varlist_profit))

  # cases
  mlist_cases <- c("cases_class"); varlist_cases <- c("Cases per 10K::[20,40)",
                                        paste0("Cases per 10K::", symbol,"40"));
  reflist_cases <- rep("[0,20)", length(varlist_cases)); mlist0_cases <- rep("cases_class", length(varlist_cases))

  # cases early
  mlist_casesearly <- c("cases_class_early"); varlist_casesearly <- c("Cases (early) per 10K::[20,40)",
                                        paste0("Cases (early) per 10K::", symbol,"40"));
  reflist_casesearly <- rep("[0,20)", length(varlist_casesearly)); mlist0_casesearly <- rep("cases_class_early",
length(varlist_casesearly))

  # cases late
  mlist_caseslate <- c("cases_class_late"); varlist_caseslate <- c("Cases (late) per 10K::[20,40)",
                                        paste0("Cases (late) per 10K::", symbol,"40"));
  reflist_caseslate <- rep("[0,20)", length(varlist_caseslate)); mlist0_caseslate <- rep("cases_class_late",
length(varlist_caseslate))

  # cases change
  mlist_caseschange <- c("cases_change"); varlist_caseschange <- c("Cases change %::[0,100)", "Cases change
%::[100,250)",
                                        paste0("Cases change %::", symbol,"250"));
  reflist_caseschange <- rep("<0%", length(varlist_caseschange)); mlist0_caseschange <- rep("cases_change",
length(varlist_caseschange))
```

© 2020 Asch DA et al. JAMA Internal Medicine.

```
# cases change binary

mlist_caseschangebinary <- c("cases_change_binary"); varlist_caseschangebinary <- c("Cases change::Increase");

reflist_caseschangebinary <- rep("Decrease", length(varlist_caseschangebinary));

mlist0_caseschangebinary <- rep("cases_change_binary", length(varlist_caseschangebinary))



# cases interaction (MATCH HOW MANY CASE BUCKETS YOU HAVE)

mlist_casesinteraction <- c("cases_interaction"); varlist_casesinteraction <- c("Cases::[20,40) & Increase",
paste0("Cases::", symbol,"40 & Increase"));

reflist_casesinteraction <- rep("otherwise", length(varlist_casesinteraction)); mlist0_casesinteraction <-
rep("cases_interaction",
                                                                     length(varlist_casesinteraction))



# cases late

mlist_caseslatediff <- c("cases_class_late_diff"); varlist_caseslatediff <- c("Cases (late) per 10K::[30,60)", "Cases (late)
per 10K::[60,90)",
                                                  paste0("Cases (late) per 10K::", symbol,"90"));

reflist_caseslatediff <- rep("[0,30)", length(varlist_caseslatediff)); mlist0_caseslatediff <- rep("cases_class_late_diff",
length(varlist_caseslatediff))



# deaths

mlist_deaths <- c("cases_class"); varlist_deaths <- c("Deaths per 10K::[1,3)", "Deaths per 10K::[3,5)",
                                     paste0("Deaths per 10K::", symbol,"5"));

reflist_deaths <- rep("[0,1)", length(varlist_deaths)); mlist0_deaths <- rep("deaths_class", length(varlist_deaths))



name2add <- name2add;
```

© 2020 Asch DA et al. JAMA Internal Medicine.

```
name2add_num <- name2add_num

## This to use for descriptive statistics
Data73 <- Data7[, colnames(Data7) %in% c("score", "cases_class", "cases_class_early",
                                    "cases_class_late", "cases_class_late_diff",
                                    "cases_change", "cases_interaction", "cases_change_binary", "deaths_class",
                                    "academic_flag", "non_profit_flag",
                                    "beds_class", "hosp_usr_class",
                                    "combination1_flag", "combination2_flag",
                                    "icu_beds_class", "resident_to_bed_ratio_class",
                                    "hosp_name", "event",
                                    "hosp_state", "hosp_region", name2add)]
if(period == "Both"){
  Data73 <- Data7[, colnames(Data7) %in% c("score", "difference", "cases_class", "cases_class_early",
                                     "cases_class_late", "cases_interaction", "cases_class_late_diff",
                                     "cases_change", "cases_change_binary", "deaths_class",
                                     "academic_flag", "non_profit_flag",
                                     "beds_class", "hosp_usr_class",
                                     "combination1_flag", "combination2_flag",
                                     "icu_beds_class", "resident_to_bed_ratio_class",
                                     "hosp_name", "event",
                                     "hosp_state", "hosp_region", name2add)]
}

Data73 <- Data73[complete.cases(Data73), ] # for descriptive statistics

Data72 <- Data7[, colnames(Data7) %in% c("score", name2add, "cases_class", "cases_class_early",
                                    "cases_class_late", "cases_interaction", "cases_class_late_diff",
                                    "cases_change", "cases_change_binary", "hosp_name", "event",
                                    "hosp_state", "hosp_region")]
dim(Data72)
```

© 2020 Asch DA et al. JAMA Internal Medicine.

```
if(period == "Both"){
  Data72 <- Data7[, colnames(Data7) %in% c("score", "cases_class", "cases_class_early",
                                   "cases_class_late", "cases_interaction", "cases_class_late_diff",
                                   "cases_change", "cases_change_binary",
                                   "difference", name2add, "hosp_name", "event",
                                   "hosp_state", "hosp_region")]
}

Data72 <- Data72[complete.cases(Data72), ]



output = list(Data7 = Data7, Data72 = Data72, Data73 = Data73,

              name2add = name2add, name2add_num = name2add_num,

              mlist_combination2 = mlist_combination2,  reflist_combination2 =  reflist_combination2,
              varlist_combination2 = varlist_combination2, mlist0_combination2 = mlist0_combination2,

              mlist_combination1 = mlist_combination1,  reflist_combination1 =  reflist_combination1,
              varlist_combination1 = varlist_combination1, mlist0_combination1 = mlist0_combination1,

              mlist_user =  mlist_user, varlist_user = varlist_user, reflist_user = reflist_user, mlist0_user  = mlist0_user,

              mlist_region = mlist_region, mlist0_region = mlist0_region, varlist_region = varlist_region, reflist_region =
reflist_region,

              mlist_profit = mlist_profit, mlist0_profit = mlist0_profit, varlist_profit = varlist_profit, reflist_profit =
reflist_profit,

              mlist_beds = mlist_beds,  reflist_beds =  reflist_beds,
```

© 2020 Asch DA et al. JAMA Internal Medicine.

mlist0_beds = mlist0_beds, varlist_beds = varlist_beds,

mlist_academic  = mlist_academic, reflist_academic  = reflist_academic,

varlist_academic = varlist_academic, mlist0_academic =  mlist0_academic,

mlist_icu = mlist_icu, reflist_icu  = reflist_icu,

mlist0_icu = mlist0_icu, varlist_icu = varlist_icu,

mlist_deaths = mlist_deaths, varlist_deaths = varlist_deaths,

reflist_deaths =   reflist_deaths, mlist0_deaths = mlist0_deaths,

mlist_cases = mlist_cases, varlist_cases = varlist_cases,

reflist_cases  =  reflist_cases, mlist0_cases = mlist0_cases,

mlist_caseschange = mlist_caseschange, varlist_caseschange = varlist_caseschange,

reflist_caseschange  =  reflist_caseschange, mlist0_caseschange = mlist0_caseschange,

mlist_caseschangebinary = mlist_caseschangebinary, varlist_caseschangebinary = varlist_caseschangebinary,

reflist_caseschangebinary  =  reflist_caseschangebinary, mlist0_caseschangebinary = mlist0_caseschangebinary,

mlist_casesearly = mlist_casesearly, varlist_casesearly = varlist_casesearly,

reflist_casesearly  =  reflist_casesearly, mlist0_casesearly = mlist0_casesearly,

mlist_caseslate = mlist_caseslate, varlist_caseslate = varlist_caseslate,

reflist_caseslate  =  reflist_caseslate, mlist0_caseslate = mlist0_caseslate,

mlist_caseslatediff = mlist_caseslatediff, varlist_caseslatediff = varlist_caseslatediff,

reflist_caseslatediff  =  reflist_caseslatediff, mlist0_caseslatediff = mlist0_caseslatediff,

© 2020 Asch DA et al. JAMA Internal Medicine.

```
            mlist_casesinteraction = mlist_casesinteraction, varlist_casesinteraction = varlist_casesinteraction,
            reflist_casesinteraction = reflist_casesinteraction, mlist0_casesinteraction = mlist0_casesinteraction,

            mlist_ratio  = mlist_ratio, varlist_ratio  = varlist_ratio, reflist_ratio = reflist_ratio, mlist0_ratio  = mlist0_ratio)
}



# A3. Not exist
'%!in%' <- function(x, y) !('%in%'(x, y))

# A4. Filtering function
filtering_function <- function(AA, BB, CC, DD, EE, FF, Main){

  Main2G <- Main
  dim(Main2G)

  if(AA != "none"){
    mystring <- AA #input$filter_desc1
    splt <- unlist(strsplit(mystring, "::"))
    AA1 <- splt[1]
    AA2 <- ifelse(splt[2] == "Yes", 'Yes', ifelse(splt[2] == "No", 'No', splt[2]))
    filter <- paste0("which(Main2G$", AA1, " == '", AA2, "')", sep = "")
    filter <- eval(parse(text = filter))
    if(length(filter) > 0){
      Main2G <- Main2G[filter, ] # creating subset by filters
    } else{
      Main2G <- Main2G
    }
  }
  dim(Main2G)
```

© 2020 Asch DA et al. JAMA Internal Medicine.

```
if(BB != "none"){

  mystring <- BB #input$filter_desc2 # then do it for second filter

  if(mystring == ">= 1mo in 2019"){
    filter2 <- which(Main2G$mm_2019 >= 1)
  } else if(mystring == ">= 2mo in 2019"){
    filter2 <- which(Main2G$mm_2019 >= 2)
  } else if(mystring == ">= 3mo in 2019"){
    filter2 <- which(Main2G$mm_2019 >= 3)
  } else if(mystring == ">= 4mo in 2019"){
    filter2 <- which(Main2G$mm_2019 >= 4)
  } else if(mystring == ">= 5mo in 2019"){
    filter2 <- which(Main2G$mm_2019 >= 5)
  } else if(mystring == ">= 6mo in 2019"){
    filter2 <- which(Main2G$mm_2019 >= 6)
  } else if(mystring == ">= 7mo in 2019"){
    filter2 <- which(Main2G$mm_2019 >= 7)
  } else if(mystring == ">= 8mo in 2019"){
    filter2 <- which(Main2G$mm_2019 >= 8)
  } else if(mystring == ">= 9mo in 2019"){
    filter2 <- which(Main2G$mm_2019 >= 9)
  } else if(mystring == ">= 10mo in 2019"){
    filter2 <- which(Main2G$mm_2019 >= 10)
  } else if(mystring == ">= 11mo in 2019"){
    filter2 <- which(Main2G$mm_2019 >= 11)
  } else if(mystring == "12mo in 2019"){
    filter2 <- which(Main2G$mm_2019 >= 12)
  }
  length(filter2)
```

© 2020 Asch DA et al. JAMA Internal Medicine.

```
  if(length(filter2) > 0){
    Main2G <- Main2G[filter2, ] # creating subset by filters
  } else{
    Main2G <- Main2G
  }

  #Main2G <- Main2G[filter2, ]

}
dim(Main2G)

CC_num <- 1
if(CC != "none"){ ## Counts

  #CC <- "Patient counts >= 10"
  CC_num <- as.numeric(strsplit(CC, ">=")[[1]][2])

  filter3 <- which(Main2G$hosp_patient_cnts >= CC_num)

  if(length(filter3) > 0){
    Main2G <- Main2G[filter3, ] # creating subset by filters
    #Main2G <- Main2G[Main2G$hosp_name %in% select_hosp, ]
  } else{
    Main2G <- Main2G
  }
}
dim(Main2G)

if(DD != "none"){# lob
  filter4 <- which(Main2G$mapd_comm_flag == DD)
```

© 2020 Asch DA et al. JAMA Internal Medicine.

```
  if(length(filter4) > 0){
    Main2G <- Main2G[filter4, ] # creating subset by filters
  } else{
    Main2G <- Main2G
  }

  #Main2G <- Main2G[which(Main2G$mapd_comm_flag == DD), ]
}
dim(Main2G)

if(EE != "none"){ #EE

  filter5 <- which(Main2G$age >=
                   as.numeric(strsplit(EE, ">=")[[1]][2]))

  if(length(filter5) > 0){
    Main2G <- Main2G[filter5, ] # creating subset by filters
  } else{
    Main2G <- Main2G
  }

}
dim(Main2G)



if(FF != "none"){ # readmitted/transferred

  filter6 <- which(Main2G$diff_hosp_flag == ifelse(FF == "Excluding readmission/transfer patients", 0, 1))

  if(length(filter6) > 0){
    Main2G <- Main2G[filter6, ] # creating subset by filters
```

© 2020 Asch DA et al. JAMA Internal Medicine.

```
    } else{
      Main2G <- Main2G
    }

    #Main2G <- Main2G[which(Main2G$diff_hosp_flag == ifelse(FF == "Excluding readmission/transfer patients", 0, 1)), ]
  }
  dim(Main2G)

  output = list(Main2G = Main2G, CC_num = CC_num)
}

# A5. Upper case
firstup <- function(x) {
  substr(x, 1, 1) <- toupper(substr(x, 1, 1))
  x
}

# A6. Set reference group in categorical data
function_character <- function(Data22, variable_list_BAR, count_variable = TRUE){

  symbol <- "\u2265"; symbol2 <- intToUtf8(8804)

  Data22 <- Data22 %>% mutate_if(is.character, as.factor)

  if(length(grep(variable_list_BAR, "Income", value = TRUE)) > 0){
    Data22 <- within(Data22, hosp_income_class <- relevel(hosp_income_class, ref = "80K < income"))
  }
  if(length(grep(variable_list_BAR, "ICU", value = TRUE)) > 0){
    Data22 <- within(Data22, icu_beds_class <- relevel(icu_beds_class, ref = "70 <= ICU"))
  }
  if(length(grep(variable_list_BAR, "Ratio", value = TRUE)) > 0){
```

© 2020 Asch DA et al. JAMA Internal Medicine.

```
  Data22 <- within(Data22, resident_to_bed_ratio_class <- relevel(resident_to_bed_ratio_class, ref = "0.50 <= Ratio"))
}
if(length(grep(variable_list_BAR, "Beds", value = TRUE)) > 0){
  Data22 <- within(Data22, beds_class <- relevel(beds_class, ref = "600 <= Beds"))
}
if(length(grep(variable_list_BAR, "Academic", value = TRUE)) > 0){
  Data22 <- within(Data22, academic_flag <- relevel(academic_flag, ref = "Academic"))
}
if(length(grep(variable_list_BAR, "Profit", value = TRUE)) > 0){
  Data22 <- within(Data22, non_profit_flag <- relevel(non_profit_flag, ref = "Non-profit"))
}
if(length(grep(variable_list_BAR, "User", value = TRUE)) > 0){
  Data22 <- within(Data22, hosp_usr_class  <- relevel(hosp_usr_class, ref = "Rural"))
}



if(length(grep(variable_list_BAR, "Race", value = TRUE)) > 0){
  Data22 <- within(Data22, race_cd_class <- relevel(race_cd_class, ref = "caucasian"))
}
if(length(grep(variable_list_BAR, "Age", value = TRUE)) > 0){
  Data22 <- within(Data22, age_class <- relevel(age_class, ref = "18 < age <= 45"))
}
if(length(grep(variable_list_BAR, "Gender", value = TRUE)) > 0){
  Data22 <- within(Data22, gender <- relevel(gender, ref = "Female"))
}
if(length(grep(variable_list_BAR, "Elix", value = TRUE)) > 0){
  Data22 <- within(Data22, elix_class <- relevel(elix_class, ref = "[0, 1)"))
}
if(count_variable  == TRUE){
  if(length(grep(variable_list_BAR, "Count", value = TRUE)) > 0){
    Data22 <- within(Data22, count_day_class <- relevel(count_day_class, ref = "150 < Count"))
```

© 2020 Asch DA et al. JAMA Internal Medicine.

```
    }
  }

  if(length(grep(variable_list_BAR, "Comorbidity", value = TRUE)) > 0){
    QQ <- paste0("Data22 <- within(Data22, ", comorb_id, " <- relevel(",comorb_id, ", ref = 'No'))", collapse = "; ")
    QQ <- eval(parse(text = QQ))
  }
  if(length(grep(variable_list_BAR, "Nurse", value = TRUE)) > 0){
    Data22 <- within(Data22, snf_nursing_flag  <- relevel(snf_nursing_flag, ref = "No"))
  }

  if(length(grep(variable_list_BAR, "LOB", value = TRUE)) > 0){
    Data22 <- within(Data22, lob  <- relevel(lob, ref = "commercial"))
  }
  if(length(grep(variable_list_BAR, "Numeric", value = TRUE)) > 0){
    Data22$count_day <- scale(Data22$count_day)
    Data22$count_day2 <- scale(Data22$count_day2)
  }

  try(Data22 <- within(Data22, mbr_death_flag <- relevel(mbr_death_flag, ref = "Alive")))

  try(Data22 <- within(Data22, mbr_mortality_flag <- relevel(mbr_mortality_flag, ref = "Alive")))


  Data22 <- Data22[order(Data22$hosp_name), ] # order by hosp name

  output = list(Data22 = Data22)
}


# B1. Single GLMM fit
```

© 2020 Asch DA et al. JAMA Internal Medicine.

```
glmmfit <- function(ylist, XXX_adj, ranlist, reml, Dataf, addsize = FALSE, laplace = TRUE){

 ## Base model (GLM) ##
 a1 <- paste0("glm(", ylist," ~ ")
 a2 <- paste0("", XXX_adj, "", collapse = " + ")
 QQ <- paste0("", a1, "", a2, ", data = Dataf, family = binomial(link = 'logit'))") # remove NAs by default
 fom <- try(eval(parse(text = QQ)))


 ## glmm
 if(laplace == TRUE){
  a1 <- paste0("glmmTMB(", ylist," ~ ", ranlist, " + ")
  a2 <- paste0("", XXX_adj, "", collapse = " + ")
  QQ <- paste0("", a1, "", a2, ", REML = reml, data = Dataf, family = binomial)") # remove NAs by default
  system.time(fom3 <- try(eval(parse(text = QQ))))
  summary(fom3)
 } else if(laplace == FALSE){
  # Get the parenthesis and what is inside
  # Remove parenthesis
  ranlist <- substring(ranlist, 2, nchar(ranlist) - 1) # extract within parenthesis
  a1 <- paste0("", ylist," ~ ")
  a2 <- paste0("", XXX_adj, "", collapse = " + ")
  QQ <- paste0("GLMMadaptive::mixed_model(fixed = ", a1, "", a2, ", random = ~", ranlist, ",
        data = Dataf, family = binomial(), control = list(nAGQ = 11))")
  system.time(fom3 <- try(eval(parse(text = QQ))))
  summary(fom3)
 }

 ## Testing variance (RE)
 x2 <- as.numeric(-2 * logLik(fom) + 2 * logLik(fom3))
 LRT_re <- 0.5 * (1 - pchisq(x2, df = 1))
```

© 2020 Asch DA et al. JAMA Internal Medicine.

```
## AIC
aic_null <- AIC(logLik(fom))
aic_alt <- AIC(logLik(fom3))

bic_null <- BIC(fom)
bic_alt <- BIC(fom3)


if(laplace == TRUE){
  ## Correlation test
  cor_lme_lmer <- cor.test(coef(fom), fixef(fom3)$cond) ## Assessing model performance (conditional/marginal R2)
  SS <- MuMIn::r.squaredGLMM(fom3) ## confidence intervals for the variances of the random intercepts
  varint <- confint(fom3)[-c(1 : length(fixef(fom3)$cond)), ]
  VIFfx <- NULL
} else if(laplace == FALSE){
  ## Correlation test
  cor_lme_lmer <- cor.test(coef(fom), fixef(fom3)) ## Assessing model performance (conditional/marginal R2)
  SS <- r2_nakagawa(fom3, by_group = FALSE)
  varint <- confint(fom3, parm = "var-cov", level = 0.95) ## confidence intervals for the variances of the random
intercepts
  VIFfx <- VIF(fom3, type = "fixed") ## Variance inflation
}

## C and Dxy: correlation between predicted probabilities and observed responses
if(ylist == "mbr_death_flag"){
  nY <- ifelse(Dataf$mbr_death_flag == "Event", 1, 0)
} else if(ylist == "mbr_mortality_flag"){
  nY <- ifelse(Dataf$mbr_mortality_flag == "Event", 1, 0)
}
probs <- binomial()$linkinv(predict(fom3))
```

© 2020 Asch DA et al. JAMA Internal Medicine.

```
rank_cor <- .somers2(probs, as.numeric(nY))



## Generating scores
sm <- summary(fom3)
if(laplace == TRUE){
  orp <- sm$coefficients$cond[-1, 1] #$coefficients[-1, 1]
  or <- exp(orp)
  VCV <- vcov(fom3, sandwich = TRUE)
  sdorp <- sqrt(diag(VCV$cond)[-1])
  BN <- exp(confint(fom3, level = 0.95))
  BN <- BN[-c(1, nrow(BN)), ] # taking-out intercept and variance
} else if(laplace == FALSE){
  orp <- sm$coef_table[-1, 1]
  or <- exp(orp)
  VCV <- vcov(fom3, sandwich = TRUE)
  sdorp <- sqrt(diag(VCV)[-c(1, length(diag(VCV)))])
  BN <- exp(confint(fom3, parm = "fixed-effects", level = 0.95))[-1, ]
}
lb <- BN[, 1]
ub <- BN[, 3]



## Estimating scores
if(laplace == TRUE){
  XFOM <- getME(fom3, "X")
  ZFOM <- getME(fom3, "Z")
  FIXFOM <- fixef(fom3)$cond
  RANFOM <- ranef(fom3)$cond$hosp_name

  LP <- XFOM %*% FIXFOM + ZFOM %*% RANFOM$`(Intercept)`
```

© 2020 Asch DA et al. JAMA Internal Medicine.

```
  FLP <- XFOM %*% FIXFOM

} else if(laplace == FALSE){
  XFOM <- model.matrix(fom3, type = "fixed")
  ZFOM  <- fastDummies::dummy_cols(Dataf$hosp_name)[, -1];
  FIXFOM <- fixef(fom3)
  RANFOM <- ranef(fom3)
  LP <- XFOM %*% FIXFOM + as.matrix(ZFOM) %*% RANFOM
  FLP <- XFOM %*% FIXFOM
}
pred <- as.vector(exp(LP) / (1 + exp(LP)))
estm <- as.vector(exp(FLP) / (1 + exp(FLP)))



if(ylist == "mbr_death_flag"){
  tb <- table(Dataf$mbr_death_flag)
  ybar <- as.numeric(tb[names(tb) %in% "Event"]) /
    length(Dataf$mbr_death_flag)
} else if(ylist == "mbr_mortality_flag"){
  tb <- table(Dataf$mbr_mortality_flag)
  ybar <- as.numeric(tb[names(tb) %in% "Event"]) /
    length(Dataf$mbr_mortality_flag)
}


### Method 1: Drye et al.
uid <- rownames(RANFOM) #rownames(ranef(fom3)$hosp_name)
score <- list()
for(ll in 1 : length(uid)){
  num <- pred[which(Dataf$hosp_name == uid[ll])]
```

© 2020 Asch DA et al. JAMA Internal Medicine.

```
    den <- estm[which(Dataf$hosp_name == uid[ll])]

    score[[ll]] <- sum(num) / sum(den) * ybar # 1/ni got cancelled out in numer and denom

  }

  score <- unlist(score)


  ### Method 2: Jeff et al.

  bigMat <- do.call(cbind, lapply(seq_len(length(uid)), function(ll){


    if(addsize == FALSE){

      # first part is specific-to-patient and second part (hypothetically) for each one of H hospitals

      if(laplace == TRUE){

        XFOM %*% FIXFOM +  # RE if subject belongs to this hospital

          RANFOM$`(Intercept)`[which(rownames(RANFOM) == uid[ll])] # logit()

      } else if(laplace == FALSE){

        XFOM %*% FIXFOM +  # RE if subject belongs to this hospital

          RANFOM[which(rownames(RANFOM) == uid[ll])] # logit()

      }


    } else if(addsize == TRUE){

      if(laplace == TRUE){

        XFOM[, colnames(XFOM) %!in% "size"] %*% FIXFOM[names(FIXFOM) %!in% "size"] + # size changes for different h

          RANFOM$`(Intercept)`[which(rownames(RANFOM) == uid[ll])] +              # RE if subject belongs to this hospital

          Dataf$size[which(Dataf$hosp_name == uid[ll])][1] * FIXFOM[names(FIXFOM) %in% "size"] # size if subject belongs
to this hospital

      } else if(laplace == FALSE){

        XFOM[, colnames(XFOM) %!in% "size"] %*% FIXFOM[names(FIXFOM) %!in% "size"] + # size changes for different h

          RANFOM[which(rownames(RANFOM) == uid[ll])] +              # RE if subject belongs to this hospital

          Dataf$size[which(Dataf$hosp_name == uid[ll])][1] * FIXFOM[names(FIXFOM) %in% "size"] # size if subject belongs
to this hospital

      }

    }
```

© 2020 Asch DA et al. JAMA Internal Medicine.

```
}))

logitinv <- function(ff) {exp(ff) / (1 + exp(ff))}
score_DS <- apply(bigMat, 2, logitinv)
score_DS_mean <- apply(score_DS, 2, mean)

## Unadjusted score
if(ylist == "mbr_death_flag"){
  unadj_score <- unlist(lapply(seq_len(length(uid)),
                               function(uu) {
                                 tr <- table(Dataf$mbr_death_flag[Dataf$hosp_name == uid[uu]])

                                 if(length(which(names(tr) %in% "Event")) > 0){
                                   tr[names(tr) %in% "Event"] / sum(tr)
                                 } else if(length(which(names(tr) %in% "Event")) == 0){
                                   0
                                 }

                               }))
  length(unadj_score)

  ## Volume
  I <- length(uid)
  hosp_orig_vol <- unlist(lapply(seq_len(I), function(ii) {
    ir1 <- which(as.numeric(Dataf$hosp_name) == ii)
    ir2 <- table(Dataf$mbr_death_flag[ir1])
    paste0("", ir2[which(names(ir2) %in% "Event")], "/", sum(ir2), "")
  }))
} else if(ylist == "mbr_mortality_flag"){
  unadj_score <- unlist(lapply(seq_len(length(uid)),
                               function(uu) {
```

© 2020 Asch DA et al. JAMA Internal Medicine.

```
                        tr <- table(Dataf$mbr_mortality_flag[Dataf$hosp_name == uid[uu]])

                        if(length(which(names(tr) %in% "Event")) > 0){
                          tr[names(tr) %in% "Event"] / sum(tr)
                        } else if(length(which(names(tr) %in% "Event")) == 0){
                          0
                        }

                      }))
  length(unadj_score)

  ## Volume
  I <- length(uid)
  hosp_orig_vol <- unlist(lapply(seq_len(I), function(ii) {
    ir1 <- which(as.numeric(Dataf$hosp_name) == ii)
    ir2 <- table(Dataf$mbr_mortality_flag[ir1])
    paste0("", ir2[which(names(ir2) %in% "Event")], "/", sum(ir2), "")
  }))


}

output = list(fom3 = fom3, LRT_re = LRT_re, aic_null = aic_null,
          aic_alt  = aic_alt, bic_null =  bic_null, bic_alt = bic_alt,
          SS = SS, cor_lme_lmer = cor_lme_lmer,
          probs = probs, rank_cor = rank_cor, varint = varint, VIFfx = VIFfx,
          nY = nY, lb = lb,
          ub = ub, sdorp = sdorp, or = or, orp = orp, pred = pred,
          estm = estm, ybar = ybar, uid = uid,
          score = score, score_DS = score_DS,
          score_DS_mean = score_DS_mean, summary = sm,
```

© 2020 Asch DA et al. JAMA Internal Medicine.

```
               unadj_score = unadj_score, hosp_orig_vol = hosp_orig_vol)
}



# B2. Single GLMM with random intercept/slope effects
glmmfit_intslope <- function(ylist, XXX_adj,
                            ranlist = "(1 + period|hosp_name)",
                            reml, Dataf_el, addsize = TRUE, sizedef = "Dynamic",
                            method = "Silber", laplace = TRUE){

  ## Volume calculation
  unm <- unique(Dataf_el$hosp_name)
  # Total (early +  late)
  Dataf_el$size_tt <- rep(NA, nrow(Dataf_el))
  for(uu in 1 : length(unm)){
    iz <- which(Dataf_el$hosp_name == unm[uu])
    Dataf_el$size_tt[iz] <- rep(length(iz), length(iz))
  }
  sum(is.na(Dataf_el$size_tt))
  summary(Dataf_el$size_tt)

  # Total (early & early+late)
  Dataf_el$size_el <- Dataf_el$size_tt #rep(NA, nrow(Dataf_el))
  for(uu in 1 : length(unm)){
    iz <- which(Dataf_el$hosp_name == unm[uu] & Dataf_el$period == 0)
    Dataf_el$size_el[iz] <- rep(length(iz), length(iz))
  }

  # Total (early & late)
  Dataf_el$size_eln <- rep(NA, nrow(Dataf_el))
  for(uu in 1 : length(unm)){
```

© 2020 Asch DA et al. JAMA Internal Medicine.

```
  iz <- which(Dataf_el$hosp_name == unm[uu] & Dataf_el$period == 0)
  Dataf_el$size_eln[iz] <- rep(length(iz), length(iz))
}
for(uu in 1 : length(unm)){
  iz <- which(Dataf_el$hosp_name == unm[uu] & Dataf_el$period == 1)
  Dataf_el$size_eln[iz] <- rep(length(iz), length(iz))
}
if(sum(is.na(Dataf_el$size_eln) > 0)){
  stop("check here")
}

if(sizedef == "Dynamic"){
  Dataf_el$size <- Dataf_el$size_el
} else if(sizedef == "Together"){
  Dataf_el$size <- Dataf_el$size_tt
} else if(sizedef == "Dynamic-non"){
  Dataf_el$size <- Dataf_el$size_eln
}
Dataf_el$size <- log(Dataf_el$size)


if("period" %!in% XXX_adj){
  XXX_adj <- c(XXX_adj, "period")
}
XXX_adj


## Base model (GLM)
a1 <- paste0("glm(", ylist," ~ 1 + period + ")
a2 <- paste0("", XXX_adj, "", collapse = " + ")
QQ <- paste0("", a1, "", a2, ", data = Dataf_el, family = binomial(link = 'logit'))") # remove NAs by default
```

© 2020 Asch DA et al. JAMA Internal Medicine.

```
fom <- try(eval(parse(text = QQ)))



## GLMM
if(laplace == TRUE){
  a1 <- paste0("glmmTMB(", ylist," ~ 1 + period + ", ranlist, " + ")
  a2 <- paste0("", XXX_adj, "", collapse = " + ")
  QQ <- paste0("", a1, "", a2, ", REML = reml, data = Dataf_el, family = binomial)") # remove NAs by default
  system.time(fom30 <- try(eval(parse(text = QQ))))

}else if(laplace == FALSE){
  # Get the parenthesis and what is inside
  # Remove parenthesis
  ranlist0 <- substring(ranlist, 2, nchar(ranlist) - 1) # extract within parenthesis
  ranlist <- strsplit(ranlist0, "+ ")[[1]][3]

  a1 <- paste0("", ylist," ~ ")
  a2 <- paste0("", XXX_adj, "", collapse = " + ")
  QQ <- paste0("GLMMadaptive::mixed_model(fixed = ", a1, "", a2, " + period, random = ~", ranlist, ",
              data = Dataf_el, family = binomial(), control = list(nAGQ = 11))")
  system.time(fom3 <- try(eval(parse(text = QQ))))
  summary(fom3)
}



## Testing variance (RE)
x2 <- as.numeric(-2 * logLik(fom) + 2 * logLik(fom3))
LRT_re <- 0.5 * (1 - pchisq(x2, df = 1))

## AIC
aic_null <- AIC(logLik(fom))
```

© 2020 Asch DA et al. JAMA Internal Medicine.

```
aic_alt <- AIC(logLik(fom3))


bic_null <- BIC(fom)

bic_alt <- BIC(fom3)


if(laplace == TRUE){

  ## Correlation test

  cor_lme_lmer <- cor.test(coef(fom), fixef(fom3)$cond) ## Assessing model performance (conditional/marginal R2)

  SS <- MuMIn::r.squaredGLMM(fom3) ## confidence intervals for the variances of the random intercepts

  varint <- confint(fom3)[-c(1 : length(fixef(fom3)$cond)), ]

  VIFfx <- NULL

} else if(laplace == FALSE){

  ## Correlation test

  cor_lme_lmer <- cor.test(coef(fom), fixef(fom3)) ## Assessing model performance (conditional/marginal R2)

  SS <- r2_nakagawa(fom3, by_group = FALSE)

  varint <- confint(fom3, parm = "var-cov", level = 0.95) ## confidence intervals for the variances of the random
intercepts

  VIFfx <- VIF(fom3, type = "fixed") ## Variance inflation

}



## C and Dxy: correlation between predicted probabilities and observed responses

if(ylist == "mbr_death_flag"){

  nY <- ifelse(Dataf_el$mbr_death_flag == "Event", 1, 0)

} else if(ylist == "mbr_mortality_flag"){

  nY <- ifelse(Dataf_el$mbr_mortality_flag == "Event", 1, 0)

}


probs <- binomial()$linkinv(predict(fom3))

rank_cor <- .somers2(probs, as.numeric(nY))
```

© 2020 Asch DA et al. JAMA Internal Medicine.

```
## Generating scores
sm <- summary(fom3)
if(laplace == TRUE){
  orp <- sm$coefficients$cond[-1, 1]
  or <- exp(orp)
  VCV <- vcov(fom3, sandwich = TRUE)
  sdorp <- sqrt(diag(VCV$cond)[-1])
  BN <- exp(confint(fom3, level = 0.95))
  BN <- BN[-c(1, nrow(BN)), ] # taking-out intercept and variance
} else if(laplace == FALSE){
  orp <- sm$coef_table[-1, 1]
  or <- exp(orp)
  VCV <- vcov(fom3, sandwich = TRUE)
  sdorp <- sqrt(diag(VCV))
  sdorp <- sdorp[names(sdorp) %in% names(or)]
  BN <- exp(confint(fom3, parm = "fixed-effects", level = 0.95))[-1, ]
}

lb <- BN[, 1]
ub <- BN[, 3]

## Estimating scores
if(laplace == TRUE){
  XFOM <- getME(fom3, "X")
  ZFOM <- getME(fom3, "Z"); dim(ZFOM)
  FIXFOM <- fixef(fom3)$cond
  RANFOM <- c(ranef(fom3)$cond$hosp_name[,1],
            ranef(fom3)$cond$hosp_name[,2])
  LP <- XFOM %*% FIXFOM + ZFOM %*% RANFOM
  FLP <- XFOM %*% FIXFOM
```

© 2020 Asch DA et al. JAMA Internal Medicine.

```
  uid <- rownames(ranef(fom3)$cond$hosp_name)

} else if(laplace == FALSE){
  XFOM <- model.matrix(fom3, type = "fixed")
  QQ <- paste0("mform <- ", ylist," ~ ",ranlist,"")
  QQ <- eval(parse(text = QQ))
  bar.f <- findbars(mform) # list with 3 terms
  mf <- model.frame(subbars(mform),data = Dataf_el)
  rt <- lme4::mkReTrms(bar.f, mf)
  ZFOM <- t(rt$Zt)

  FIXFOM <- fixef(fom3)
  RANFOM <- ranef(fom3)
  LP <- XFOM %*% FIXFOM + as.matrix(ZFOM) %*% as.vector(t(RANFOM))
  FLP <- XFOM %*% FIXFOM

  uid <- rownames(ranef(fom3))
}
pred <- as.vector(exp(LP) / (1 + exp(LP)))
estm <- as.vector(exp(FLP) / (1 + exp(FLP)))



if(ylist == "mbr_death_flag"){
  tb <- table(Dataf_el$mbr_death_flag)
  ybar <- as.numeric(tb[names(tb) %in% "Event"]) /
    length(Dataf_el$mbr_death_flag)
} else if(ylist == "mbr_mortality_flag"){
  tb <- table(Dataf_el$mbr_mortality_flag)
  ybar <- as.numeric(tb[names(tb) %in% "Event"]) /
```

© 2020 Asch DA et al. JAMA Internal Medicine.

```
    length(Dataf_el$mbr_mortality_flag)
}




## Score caluclation based on Method 1: Drye et al.
score <- score_early2 <-  score_early2j <- score_later2 <- score_later2j <- list()
for(ll in 1 : length(uid)){
  num <- pred[which(Dataf_el$hosp_name == uid[ll])]
  den <- estm[which(Dataf_el$hosp_name == uid[ll])]
  score[[ll]] <- sum(num) / sum(den) * ybar # 1/ni got cancelled out in numer and denom
}
score <- unlist(score)

## Early score Method 1: Drye et al.
if(ylist == "mbr_death_flag"){
  tb <- table(Dataf_el$mbr_death_flag[which(Dataf_el$period == 0)])
  ybar_early <- as.numeric(tb[names(tb) %in% "Event"]) /
    length(Dataf_el$mbr_death_flag[which(Dataf_el$period == 0)])
} else if(ylist == "mbr_mortality_flag"){
  tb <- table(Dataf_el$mbr_mortality_flag[which(Dataf_el$period == 0)])
  ybar_early <- as.numeric(tb[names(tb) %in% "Event"]) /
    length(Dataf_el$mbr_mortality_flag[which(Dataf_el$period == 0)])

}



for(ll in 1 : length(uid)){
  num <- pred[which(Dataf_el$hosp_name == uid[ll] & Dataf_el$period == 0)]
  den <- estm[which(Dataf_el$hosp_name == uid[ll] & Dataf_el$period == 0)]
```

© 2020 Asch DA et al. JAMA Internal Medicine.

```
  score_early2[[ll]] <- sum(num) / sum(den) * ybar
  score_early2j[[ll]] <- sum(num) / sum(den) * ybar_early # 1/ni got cancelled out in numer and denom
}
score_early2 <- unlist(score_early2)
score_early2j <- unlist(score_early2j)


## Late score Method 1: Drye et al.
if(ylist == "mbr_death_flag"){
  tb <- table(Dataf_el$mbr_death_flag[which(Dataf_el$period == 1)])
  ybar_later <- as.numeric(tb[names(tb) %in% "Event"]) /
    length(Dataf_el$mbr_death_flag[which(Dataf_el$period == 1)])
} else if(ylist == "mbr_mortality_flag"){
  tb <- table(Dataf_el$mbr_mortality_flag[which(Dataf_el$period == 1)])
  ybar_later <- as.numeric(tb[names(tb) %in% "Event"]) /
    length(Dataf_el$mbr_mortality_flag[which(Dataf_el$period == 1)])
}


for(ll in 1 : length(uid)){
  num <- pred[which(Dataf_el$hosp_name == uid[ll] & Dataf_el$period == 1)]
  den <- estm[which(Dataf_el$hosp_name == uid[ll] & Dataf_el$period == 1)]
  score_later2[[ll]] <- sum(num) / sum(den) * ybar
  score_later2j[[ll]] <- sum(num) / sum(den) * ybar_later # 1/ni got cancelled out in numer and denom
}
score_later2 <- unlist(score_later2)
score_later2j <- unlist(score_later2j)
summary(score_later2)
summary(score_later2j)
```

© 2020 Asch DA et al. JAMA Internal Medicine.

```
length(score_early2); length(score_later2)

### Score calculation based on Method 2: Silber et al.

## Bigmat (Overall) : NOT USED
bigMat <- do.call(cbind, lapply(seq_len(length(uid)), function(ll){

  if(laplace == TRUE){
    if(addsize == FALSE){
      # first part is specific-to-patient and second part (hypothetically) for each one of H hospitals
      XFOM %*% FIXFOM +      # RE if subject belongs to this hospital
        Dataf_el$period * ranef(fom3)$cond$hosp_name[which(rownames(ranef(fom3)$cond$hosp_name)  == uid[ll]), 2]
+
        ranef(fom3)$cond$hosp_name[which(rownames(ranef(fom3)$cond$hosp_name)  == uid[ll]), 1]
    } else if(addsize == TRUE){
      XFOM[, colnames(XFOM) %!in% "size"] %*% FIXFOM[names(FIXFOM) %!in% "size"] +  # RE if subject belongs to this
hospital
        ranef(fom3)$cond$hosp_name[which(rownames(ranef(fom3)$cond$hosp_name)  == uid[ll]), 1] +
        Dataf_el$period * ranef(fom3)$cond$hosp_name[which(rownames(ranef(fom3)$cond$hosp_name)  == uid[ll]), 2]
+
        mean(Dataf_el$size[which(Dataf_el$hosp_name == uid[ll])]) * FIXFOM[names(FIXFOM) %in% "size"] # as size
changes
    } # taking mean(Dataf_el$size[]) as size changes between early and late for each hospital
  }else if(laplace == FALSE){
    if(addsize == FALSE){
      # first part is specific-to-patient and second part (hypothetically) for each one of H hospitals
      XFOM %*% FIXFOM +      # RE if subject belongs to this hospital
        Dataf_el$period * ranef(fom3)[which(rownames(ranef(fom3))  == uid[ll]), 2] +
        ranef(fom3)[which(rownames(ranef(fom3))  == uid[ll]), 1]
    } else if(addsize == TRUE){
      XFOM[, colnames(XFOM) %!in% "size"] %*% FIXFOM[names(FIXFOM) %!in% "size"] +  # RE if subject belongs to this
hospital
        ranef(fom3)[which(rownames(ranef(fom3))  == uid[ll]), 1] +
```

© 2020 Asch DA et al. JAMA Internal Medicine.

```
        Dataf_el$period * ranef(fom3)[which(rownames(ranef(fom3))  == uid[ll]), 2] +
        mean(Dataf_el$size[which(Dataf_el$hosp_name == uid[ll])]) * FIXFOM[names(FIXFOM) %in% "size"] # as size
changes
    }


   }
 }))
 logitinv <- function(ff) {exp(ff) / (1 + exp(ff))}
 score_DS <- apply(bigMat, 2, logitinv)
 score_DS_mean <- apply(score_DS, 2, mean)



 ## Early Method 2: Silber (exploting BOTH periods info)
 bigMat_early <- do.call(cbind, lapply(seq_len(length(uid)), function(ll){


   if(laplace == TRUE){
    if(addsize == FALSE){
      # first part is specific-to-patient and second part (hypothetically) for each one of H hospitals
      XFOM[, colnames(XFOM) %!in% "period"] %*% FIXFOM[colnames(XFOM) %!in% "period"] +      # RE if subject
belongs to this hospital
        0 * FIXFOM[colnames(XFOM) %in% "period"] + # adjusted for fixed early time-effects
        0 * ranef(fom3)$cond$hosp_name[which(rownames(ranef(fom3)$cond$hosp_name)  == uid[ll]), 2] +
        ranef(fom3)$cond$hosp_name[which(rownames(ranef(fom3)$cond$hosp_name)  == uid[ll]), 1]
    } else if(addsize == TRUE){
      XFOM[ , colnames(XFOM) %!in% c("size", "period")] %*% FIXFOM[names(FIXFOM) %!in% c("size", "period")] +  # RE
if subject belongs to this hospital
        0 * FIXFOM[colnames(XFOM) %in% "period"] + # adjusted for fixed time- effects
        ranef(fom3)$cond$hosp_name[which(rownames(ranef(fom3)$cond$hosp_name)  == uid[ll]), 1] + # random
intercept for hypothetical site
        0 * ranef(fom3)$cond$hosp_name[which(rownames(ranef(fom3)$cond$hosp_name)  == uid[ll]), 2] + # random
slope for hypothetical site
        Dataf_el$size[which(Dataf_el$hosp_name == uid[ll] & Dataf_el$period == 0)][1] * FIXFOM[names(FIXFOM) %in%
"size"] # size for hypothetical site
```

© 2020 Asch DA et al. JAMA Internal Medicine.

```
    }
   } else if(laplace == FALSE){
     if(addsize == FALSE){
       # first part is specific-to-patient and second part (hypothetically) for each one of H hospitals
       XFOM[, colnames(XFOM) %!in% "period"] %*% FIXFOM[colnames(XFOM) %!in% "period"] +      # RE if subject belongs to this hospital
         0 * FIXFOM[colnames(XFOM) %in% "period"] + # adjusted for fixed early time-effects
         0 * ranef(fom3)[which(rownames(ranef(fom3))  == uid[ll]), 2] +
         ranef(fom3)[which(rownames(ranef(fom3))  == uid[ll]), 1]
     } else if(addsize == TRUE){
       XFOM[ , colnames(XFOM) %!in% c("size", "period")] %*% FIXFOM[names(FIXFOM) %!in% c("size", "period")] +  # RE if subject belongs to this hospital
         0 * FIXFOM[colnames(XFOM) %in% "period"] + # adjusted for fixed time- effects
         ranef(fom3)[which(rownames(ranef(fom3))  == uid[ll]), 1] + # random intercept for hypothetical site
         0 * ranef(fom3)[which(rownames(ranef(fom3))  == uid[ll]), 2] + # random slope for hypothetical site
         Dataf_el$size[which(Dataf_el$hosp_name == uid[ll] & Dataf_el$period == 0)][1] * FIXFOM[names(FIXFOM) %in% "size"] # size for hypothetical site
     }
   }
  }))
  score_DS_early <- apply(bigMat_early, 2, logitinv)
  score_DS_mean_early <- apply(score_DS_early, 2, mean)



  ## Late Method 2: Silber et al.
  bigMat_late <- do.call(cbind, lapply(seq_len(length(uid)), function(ll){

   if(laplace == TRUE){
     if(addsize == FALSE){
       # first part is specific-to-patient and second part (hypothetically) for each one of H hospitals
       XFOM[, colnames(XFOM) %!in% "period"] %*% FIXFOM[colnames(XFOM) %!in% "period"] +      # RE if subject belongs to this hospital
```

© 2020 Asch DA et al. JAMA Internal Medicine.

```
        1 * FIXFOM[colnames(XFOM) %in% "period"] + #adjusted for fixed late
        1 * ranef(fom3)$cond$hosp_name[which(rownames(ranef(fom3)$cond$hosp_name)  == uid[ll]), 2] +
        ranef(fom3)$cond$hosp_name[which(rownames(ranef(fom3)$cond$hosp_name)  == uid[ll]), 1]
    } else if(addsize == TRUE){
      XFOM[, colnames(XFOM) %!in% c("size", "period")] %*% FIXFOM[names(FIXFOM) %!in% c("size", "period")] +  # RE if subject belongs to this hospital
        1 * FIXFOM[colnames(XFOM) %in% "period"] + #adjusted for fixed late effect
        ranef(fom3)$cond$hosp_name[which(rownames(ranef(fom3)$cond$hosp_name)  == uid[ll]), 1] + # random intercept for hypothetical site
        1 * ranef(fom3)$cond$hosp_name[which(rownames(ranef(fom3)$cond$hosp_name)  == uid[ll]), 2] +# random slope for hypothetical site
        Dataf_el$size[which(Dataf_el$hosp_name == uid[ll] & Dataf_el$period == 1)][1] * FIXFOM[names(FIXFOM) %in% "size"] # fixed size for hypothetical site
      # size changes in early/late. Hypothetically each patient comes from each period and each hospital
    }
  } else if(laplace == FALSE){
    if(addsize == FALSE){
      # first part is specific-to-patient and second part (hypothetically) for each one of H hospitals
      XFOM[, colnames(XFOM) %!in% "period"] %*% FIXFOM[colnames(XFOM) %!in% "period"] +      # RE if subject belongs to this hospital
        1 * FIXFOM[colnames(XFOM) %in% "period"] + #adjusted for fixed late
        1 * ranef(fom3)[which(rownames(ranef(fom3))  == uid[ll]), 2] +
        ranef(fom3)[which(rownames(ranef(fom3))  == uid[ll]), 1]
    } else if(addsize == TRUE){
      XFOM[, colnames(XFOM) %!in% c("size", "period")] %*% FIXFOM[names(FIXFOM) %!in% c("size", "period")] +  # RE if subject belongs to this hospital
        1 * FIXFOM[colnames(XFOM) %in% "period"] + #adjusted for fixed late effect
        ranef(fom3)[which(rownames(ranef(fom3))  == uid[ll]), 1] + # random intercept for hypothetical site
        1 * ranef(fom3)[which(rownames(ranef(fom3))  == uid[ll]), 2] + # random slope for hypothetical site
        Dataf_el$size[which(Dataf_el$hosp_name == uid[ll] & Dataf_el$period == 1)][1] * FIXFOM[names(FIXFOM) %in% "size"] # fixed size for hypothetical site
      # size changes in early/late. Hypothetically each patient comes from each period and each hospital
    }
```

© 2020 Asch DA et al. JAMA Internal Medicine.

```
  }
 }))
 score_DS_late <- apply(bigMat_late, 2, logitinv)
 score_DS_mean_late <- apply(score_DS_late, 2, mean)




 ## Early Method 2: Silber et al. (early and late separate 10K for early and 17K for late kind of)
 bigMat_early2 <- do.call(cbind, lapply(seq_len(length(uid)), function(ll){

   if(laplace == TRUE){
    if(addsize == FALSE){
      # first part is specific-to-patient and second part (hypothetically) for each one of H hospitals
      XFOM[which(Dataf_el$period == 0), ] %*% FIXFOM +      # RE if subject belongs to this hospital
        Dataf_el$period[which(Dataf_el$period == 0)] *
ranef(fom3)$cond$hosp_name[which(rownames(ranef(fom3)$cond$hosp_name)  == uid[ll]), 2] +
        ranef(fom3)$cond$hosp_name[which(rownames(ranef(fom3)$cond$hosp_name)  == uid[ll]), 1]
    } else if(addsize == TRUE){
      XFOM[which(Dataf_el$period == 0), colnames(XFOM) %!in% "size"] %*% FIXFOM[names(FIXFOM) %!in% "size"] +  #
RE if subject belongs to this hospital
        ranef(fom3)$cond$hosp_name[which(rownames(ranef(fom3)$cond$hosp_name)  == uid[ll]), 1] + #
        Dataf_el$period[which(Dataf_el$period == 0)] *
ranef(fom3)$cond$hosp_name[which(rownames(ranef(fom3)$cond$hosp_name)  == uid[ll]), 2] +
        Dataf_el$size[which(Dataf_el$hosp_name == uid[ll] & Dataf_el$period == 0)][1] * FIXFOM[names(FIXFOM) %in%
"size"]
    }
   } else if(laplace == FALSE){
    if(addsize == FALSE){
      # first part is specific-to-patient and second part (hypothetically) for each one of H hospitals
      XFOM[which(Dataf_el$period == 0), ] %*% FIXFOM +      # RE if subject belongs to this hospital
        Dataf_el$period[which(Dataf_el$period == 0)] * ranef(fom3)[which(rownames(ranef(fom3))  == uid[ll]), 2] +
```

© 2020 Asch DA et al. JAMA Internal Medicine.

```
        ranef(fom3)[which(rownames(ranef(fom3))  == uid[ll]), 1]

      } else if(addsize == TRUE){

        XFOM[which(Dataf_el$period == 0), colnames(XFOM) %!in% "size"] %*% FIXFOM[names(FIXFOM) %!in% "size"] +  # 
RE if subject belongs to this hospital

          ranef(fom3)[which(rownames(ranef(fom3))  == uid[ll]), 1] + #

          Dataf_el$period[which(Dataf_el$period == 0)] * ranef(fom3)[which(rownames(ranef(fom3))  == uid[ll]), 2] +

          Dataf_el$size[which(Dataf_el$hosp_name == uid[ll] & Dataf_el$period == 0)][1] * FIXFOM[names(FIXFOM) %in% 
"size"]

      }

    }

  }))

  score_DS_early2 <- apply(bigMat_early2, 2, logitinv)

  score_DS_mean_early2 <- apply(score_DS_early2, 2, mean)



  ## Late Method 2: Silber et al.

  bigMat_late2 <- do.call(cbind, lapply(seq_len(length(uid)), function(ll){

    if(laplace == TRUE){

      if(addsize == FALSE){

        # first part is specific-to-patient and second part (hypothetically) for each one of H hospitals

        XFOM[which(Dataf_el$period == 1), ] %*% FIXFOM +      # RE if subject belongs to this hospital

          Dataf_el$period[which(Dataf_el$period == 1)] * 
ranef(fom3)$cond$hosp_name[which(rownames(ranef(fom3)$cond$hosp_name)  == uid[ll]), 2] +

          ranef(fom3)$cond$hosp_name[which(rownames(ranef(fom3)$cond$hosp_name)  == uid[ll]), 1]

      } else if(addsize == TRUE){

        XFOM[which(Dataf_el$period == 1), colnames(XFOM) %!in% "size"] %*% FIXFOM[names(FIXFOM) %!in% "size"] +  # 
RE if subject belongs to this hospital

          ranef(fom3)$cond$hosp_name[which(rownames(ranef(fom3)$cond$hosp_name)  == uid[ll]), 1] + #

          Dataf_el$period[which(Dataf_el$period == 1)] * 
ranef(fom3)$cond$hosp_name[which(rownames(ranef(fom3)$cond$hosp_name)  == uid[ll]), 2] +

          Dataf_el$size[which(Dataf_el$hosp_name == uid[ll] & Dataf_el$period == 1)][1] * FIXFOM[names(FIXFOM) %in% 
"size"]

      }
```

© 2020 Asch DA et al. JAMA Internal Medicine.

```
   } else if(laplace == FALSE){
     if(addsize == FALSE){
       # first part is specific-to-patient and second part (hypothetically) for each one of H hospitals
       XFOM[which(Dataf_el$period == 1), ] %*% FIXFOM +      # RE if subject belongs to this hospital
         Dataf_el$period[which(Dataf_el$period == 1)] * ranef(fom3)[which(rownames(ranef(fom3))  == uid[ll]), 2] +
         ranef(fom3)[which(rownames(ranef(fom3))  == uid[ll]), 1]
     } else if(addsize == TRUE){
       XFOM[which(Dataf_el$period == 1), colnames(XFOM) %!in% "size"] %*% FIXFOM[names(FIXFOM) %!in% "size"] +  #
RE if subject belongs to this hospital
         ranef(fom3)[which(rownames(ranef(fom3))  == uid[ll]), 1] + #
         Dataf_el$period[which(Dataf_el$period == 1)] * ranef(fom3)[which(rownames(ranef(fom3))  == uid[ll]), 2] +
         Dataf_el$size[which(Dataf_el$hosp_name == uid[ll] & Dataf_el$period == 1)][1] * FIXFOM[names(FIXFOM) %in%
"size"]
     }
   }
  }))
  score_DS_late2 <- apply(bigMat_late2, 2, logitinv)
  score_DS_mean_late2 <- apply(score_DS_late2, 2, mean)



  me_early <- 1.96 * apply(score_DS_early, 2, sd) / sqrt(nrow(score_DS_early))
  score_boot_q1_early <- apply(score_DS_early, 2, summary_25l)
  score_boot_q3_early <- apply(score_DS_early, 2, summary_75l)
  score_boot_025_early <- apply(score_DS_early, 2, summary_025l)
  score_boot_975_early <- apply(score_DS_early, 2, summary_975l)

  me_late <- 1.96 * apply(score_DS_late, 2, sd) / sqrt(nrow(score_DS_late))
  score_boot_q1_late <- apply(score_DS_late, 2, summary_25l)
  score_boot_q3_late <- apply(score_DS_late, 2, summary_75l)
  score_boot_025_late <- apply(score_DS_late, 2, summary_025l)
  score_boot_975_late <- apply(score_DS_late, 2, summary_975l)
```

© 2020 Asch DA et al. JAMA Internal Medicine.

```
## Bland-altman plot
if(method == "Drye"){
  s1 <- score_later2j - score_early2j
  s2 <- (score_later2j + score_early2j) / 2
} else if(method == "Silber"){
  s1 <- score_DS_mean_late - score_DS_mean_early
  s2 <- (score_DS_mean_late + score_DS_mean_early) / 2
}

## Volume
I <- length(uid)

## Unadjusted score
if(ylist == "mbr_death_flag"){
  hosp_orig_vol_early <- unlist(lapply(seq_len(I),
                                  function(ii) {
                                    ir1 <- which(Dataf_el$hosp_name == uid[ii] & Dataf_el$period == 0)
                                    ir2 <- table(Dataf_el$mbr_death_flag[ir1])
                                    paste0("", ir2[which(names(ir2) %in% "Event")], "/", sum(ir2), "")
                                  }))

  hosp_orig_vol_later <- unlist(lapply(seq_len(I), function(ii) {
    ir1 <- which(Dataf_el$hosp_name == uid[ii] & Dataf_el$period == 1)
    ir2 <- table(Dataf_el$mbr_death_flag[ir1])
    paste0("", ir2[which(names(ir2) %in% "Event")], "/", sum(ir2), "")
  }))

  unadj_score <- unlist(lapply(seq_len(length(uid)),
```

© 2020 Asch DA et al. JAMA Internal Medicine.

```
                          function(uu) {
                            tr <- table(Dataf_el$mbr_death_flag[Dataf_el$hosp_name == uid[uu]])


                            if(length(which(names(tr) %in% "Event")) > 0){
                              tr[names(tr) %in% "Event"] / sum(tr)
                            } else if(length(which(names(tr) %in% "Event")) == 0){
                              0
                            }


                          }))
  hosp_orig_vol <- unlist(lapply(seq_len(I), function(ii) {
    ir1 <- which(as.numeric(Dataf_el$hosp_name) == ii)
    ir2 <- table(Dataf_el$mbr_death_flag[ir1])
    paste0("", ir2[which(names(ir2) %in% "Event")], "/", sum(ir2), "")
  }))

} else if(ylist == "mbr_mortality_flag"){

  hosp_orig_vol_early <- unlist(lapply(seq_len(I),
                                function(ii) {
                                  ir1 <- which(Dataf_el$hosp_name == uid[ii] & Dataf_el$period == 0)
                                  ir2 <- table(Dataf_el$mbr_mortality_flag[ir1])
                                  paste0("", ir2[which(names(ir2) %in% "Event")], "/", sum(ir2), "")
                                }))

  hosp_orig_vol_later <- unlist(lapply(seq_len(I), function(ii) {
    ir1 <- which(Dataf_el$hosp_name == uid[ii] & Dataf_el$period == 1)
    ir2 <- table(Dataf_el$mbr_mortality_flag[ir1])
    paste0("", ir2[which(names(ir2) %in% "Event")], "/", sum(ir2), "")
  }))
```

© 2020 Asch DA et al. JAMA Internal Medicine.

```
  unadj_score <- unlist(lapply(seq_len(length(uid)),
                          function(uu) {
                            tr <- table(Dataf_el$mbr_mortality_flag[Dataf_el$hosp_name == uid[uu]])

                            if(length(which(names(tr) %in% "Event")) > 0){
                              tr[names(tr) %in% "Event"] / sum(tr)
                            } else if(length(which(names(tr) %in% "Event")) == 0){
                              0
                            }

                          }))

  hosp_orig_vol <- unlist(lapply(seq_len(I), function(ii) {
    ir1 <- which(as.numeric(Dataf_el$hosp_name) == ii)
    ir2 <- table(Dataf_el$mbr_mortality_flag[ir1])
    paste0("", ir2[which(names(ir2) %in% "Event")], "/", sum(ir2), "")
  }))

}




hosp_orig_vol_early[1]; hosp_orig_vol_later[1]
length(unadj_score)



output = list(fom3 = fom3, LRT_re = LRT_re, aic_null = aic_null, aic_alt  = aic_alt,
              varint = varint, VIFfx = VIFfx, bic_null = bic_null, bic_alt = bic_alt,
              score_early2 = score_early2, score_later2 = score_later2,
              SS = SS, cor_lme_lmer = cor_lme_lmer,
              probs = probs, rank_cor = rank_cor, nY = nY, lb = lb,
```

© 2020 Asch DA et al. JAMA Internal Medicine.

```
            ub = ub, sdorp = sdorp, or = or, orp = orp, pred = pred,
            estm = estm, ybar = ybar, uid = uid, score = score, summary = sm,
            unadj_score = unadj_score, hosp_orig_vol_later = hosp_orig_vol_later,
            hosp_orig_vol_early = hosp_orig_vol_early,
            hosp_orig_vol = hosp_orig_vol,
            s1 = s1, s2 = s2,  score_later2j =  score_later2j,
            score_later2 =  score_later2j,  score_early2j =  score_early2j,
            score_early2 =  score_early2, ybar_early = ybar_early,
            ybar_later = ybar_later,

            ## Results for Silber
            score_DS_late = score_DS_late, score_DS_late2 = score_DS_late2,
            score_DS_mean_late = score_DS_mean_late, score_DS_mean_late2 = score_DS_mean_late2,

            me_late = me_late,  score_boot_q1_late =  score_boot_q1_late, score_boot_q3_late = score_boot_q3_late,
            score_boot_025_late = score_boot_025_late, score_boot_975_late  = score_boot_975_late,

            score_DS_early = score_DS_early, score_DS_early2 = score_DS_early2,
            score_DS_mean_early  = score_DS_mean_early, score_DS_mean_early2  = score_DS_mean_early2,

            me_early =  me_early, score_boot_q1_early = score_boot_q1_early, score_boot_q3_early =
score_boot_q3_early,
            score_boot_025_early = score_boot_025_early, score_boot_975_early = score_boot_975_early
  )
}



# B3. Hospital outcome regression
hospital_outcome_regression <- function(Data_analytical, Data_score,
                              hosp_id = c("hosp_usr_class", "hosp_income_class", "hosp_region", "hosp_state",
"snf_nursing_flag", "beds_class", "icu_beds_class", "resident_to_bed_ratio_class", "non_profit_flag","academic_flag"),
```

© 2020 Asch DA et al. JAMA Internal Medicine.

```
                        apnd2,
                        reml = FALSE,
                        period = FALSE,
                        transformation = "scale",
                        glmlog = FALSE,
                        allsite = "No",
                        ranlist = "(1 | hosp_state)",
                        addregion = TRUE,
                        name2add = c("icu_beds_class", "resident_to_bed_ratio_class", "beds_class",
                              "non_profit_flag", "hosp_usr_class", "deaths_class"),
                        name2add_num  = c("icu_beds", "resident_to_bed_ratio", "beds", "non_profit_flag",
                                "hosp_usr_class", "deaths_class"),
                        varlist_bar = "ICU:Ratio:Beds:Profit:User:Deaths"){

symbol <- "\u2265"; symbol2 <- intToUtf8(8804)

length(unique(Data_analytical$hosp_name))
am3 <- arrange_dataf_hospital(Data_score, Data_analytical, hosp_id, apnd2, period = period,
                    allsite = allsite, name2add = name2add, name2add_num = name2add_num)
Data7 <- am3$Data7; Data72 <- am3$Data72; Data73 <- am3$Data73

mlist_combination1 <- am3$mlist_combination1; varlist_combination1 <- am3$varlist_combination1;
reflist_combination1 <- am3$reflist_combination1; mlist0_combination1 <- am3$mlist0_combination1

mlist_casesinteraction <- am3$mlist_casesinteraction; varlist_casesinteraction <- am3$varlist_casesinteraction;
reflist_casesinteraction <- am3$reflist_casesinteraction; mlist0_casesinteraction <- am3$mlist0_casesinteraction


mlist_combination2 <- am3$mlist_combination2; varlist_combination2 <- am3$varlist_combination2;
reflist_combination2 <- am3$reflist_combination2; mlist0_combination2 <- am3$mlist0_combination2
```

© 2020 Asch DA et al. JAMA Internal Medicine.

```
mlist_beds <- am3$mlist_beds; try(mlist_academic <- am3$mlist_academic);
mlist_icu <- am3$mlist_icu; mlist_ratio <- am3$mlist_ratio;
mlist_user <- am3$mlist_user; try(mlist_profit <- am3$mlist_profit);
mlist_region <- am3$mlist_region; mlist_cases <- am3$mlist_cases; mlist_deaths <- am3$mlist_deaths
mlist_casesearly <- am3$mlist_casesearly; mlist_caseslate <- am3$mlist_caseslate;
mlist_caseslatediff <- am3$mlist_caseslatediff; mlist_caseschange <- am3$mlist_caseschange;
mlist_caseschangebinary <- am3$mlist_caseschangebinary

reflist_beds <- am3$reflist_beds; try(reflist_academic <- am3$reflist_academic);
reflist_icu <- am3$reflist_icu; reflist_ratio <- am3$reflist_ratio;
reflist_user <- am3$reflist_user; try(reflist_profit <- am3$reflist_profit);
reflist_region <- am3$reflist_region; reflist_cases <- am3$reflist_cases; reflist_deaths <- am3$reflist_deaths
reflist_casesearly <- am3$reflist_casesearly; reflist_caseslate <- am3$reflist_caseslate;
reflist_caseslatediff <- am3$reflist_caseslatediff; reflist_caseschange <- am3$reflist_caseschange
reflist_caseschangebinary <- am3$reflist_caseschangebinary

mlist0_beds <- am3$mlist0_beds; try(mlist0_academic <- am3$mlist0_academic);
mlist0_icu <- am3$mlist0_icu; mlist0_ratio <- am3$mlist0_ratio
mlist0_user <- am3$mlist0_user; try(mlist0_profit <- am3$mlist0_profit);
mlist0_region <- am3$mlist0_region;  mlist0_cases <- am3$mlist0_cases; mlist0_deaths <- am3$mlist0_deaths
mlist0_casesearly <- am3$mlist0_casesearly; mlist0_caseslate <- am3$mlist0_caseslate;
mlist0_caseslatediff <- am3$mlist0_caseslatediff; mlist0_caseschange <- am3$mlist0_caseschange
mlist0_caseschangebinary <- am3$mlist0_caseschangebinary

varlist_beds <- am3$varlist_beds; varlist_academic <- am3$varlist_academic;
varlist_icu <- am3$varlist_icu; varlist_ratio <- am3$varlist_ratio;
varlist_user <- am3$varlist_user; try(varlist_profit <- am3$varlist_profit);
varlist_region <- am3$varlist_region; varlist_cases <- am3$varlist_cases; varlist_deaths <- am3$varlist_deaths
varlist_casesearly <- am3$varlist_casesearly; varlist_caseslate <- am3$varlist_caseslate;
varlist_caseslatediff <- am3$varlist_caseslatediff; varlist_caseschange <- am3$varlist_caseschange
varlist_caseschangebinary <- am3$varlist_caseschangebinary
```

© 2020 Asch DA et al. JAMA Internal Medicine.

```
if(addregion == TRUE){
  name2add <- c(am3$name2add, "hosp_region")
} else{
  name2add <- am3$name2add
}


## Numeric scores
if(period != "Both"){
  Data72$scoreScale <- scale(Data72$score) # - mean(Data72$score)
  Data72$scoreLog <- log(Data72$score)
  Data72$scoreSqrt <- sqrt(Data72$score)

  if(transformation == "scale"){
    Data72$scoreme <- as.numeric(Data72$scoreScale)
  } else if(transformation == "log"){
    Data72$scoreme <- as.numeric(log(Data72$score))
  } else if(transformation == "sqrt"){
    Data72$scoreme <- as.numeric(sqrt(Data72$score))
  } else if(transformation == "min-max"){
    Data72$scoreme <- (Data72$score - min(Data72$score)) /
      (max(Data72$score) - min(Data72$score))
  } else if(transformation == "original"){
    Data72$scoreme <- as.numeric(Data72$score)
  }
} else if(period == "Both"){
  Data72$scoreme <- Data72$difference
}

## LRT for each variables GLM with random effects
```

© 2020 Asch DA et al. JAMA Internal Medicine.

```
if(glmlog == FALSE){
  a1 <- paste0("lm(scoreme ~ ")
  a2 <- paste0("", name2add, "", collapse = " + ")
  QQ <- paste0("", a1, "", a2, ", singular.ok = TRUE, data = Data72)")
  fit11<- eval(parse(text = QQ))
} else if(glmlog == TRUE){
  a1 <- paste0("glm(scoreme ~ ") #
  a2 <- paste0("", name2add, "", collapse = " + ")
  QQ <- paste0("", a1, "", a2, " , singular.ok = TRUE,
             family = gaussian(link = 'log'),
             data = Data72)")
  fit11 <- eval(parse(text = QQ))
}
summary(fit11)
dim(Data72)
length(Data72$hosp_name)

vif <- DescTools::VIF(fit11)
vif

# Full model mixed
if(is.null(ranlist) == FALSE){
  fit1 <- lmer(as.formula(paste0('scoreme ~ ', paste0(name2add, collapse = '+' ), '+', ranlist,'')),
             data = Data72, REML = reml)
  try(aic_alt <- AIC(logLik(fit1)))
} else{
  fit1 <- aic_alt <- NULL
}

test_table1 <- test_table2 <- list(); fit0 <- NULL
for(ii in 1 : length(name2add)){
```

© 2020 Asch DA et al. JAMA Internal Medicine.

```
  ## Null Hypothesis
  if(glmlog == FALSE){
   a1 <- paste0("lm(scoreme ~ ")
   a2 <- paste0("", name2add[name2add %!in% name2add[ii]], "", collapse = " + ")
   QQ <- paste0("", a1, "", a2, ", singular.ok = TRUE, data = Data72)")
   fit00 <- eval(parse(text = QQ))

   if(is.null(ranlist) == FALSE){
    fit0 <- lmer(as.formula(paste0('scoreme ~ ', paste0(name2add[name2add %!in% name2add[ii]],
                                  collapse = '+' ), '+', ranlist,'')), data = Data72, REML = reml)
    test_table2[[ii]] <- stats::anova(fit0, fit1, test = "LRT")
   } else{
    fit0 <- NULL
   }
  } else if(glmlog == TRUE){

   a1 <- paste0("glm(scoreme ~ ")
   a2 <- paste0("", name2add[name2add %!in% name2add[ii]], "", collapse = " + ")
   QQ <- paste0("", a1, "", a2, ", singular.ok = TRUE, family = gaussian(link = 'log'),
          data = Data72)")
   fit00 <- eval(parse(text = QQ))
  }
  test_table1[[ii]] <- stats::anova(fit00, fit11, test = "LRT")
}


if(is.null(ranlist) == FALSE) {
  if(isSingular(fit1) == TRUE){
   fit_final <- fit11 # LM
  } else{
```

© 2020 Asch DA et al. JAMA Internal Medicine.

```
    fit_final <- fit1 # Mixed
  }
} else{
  fit_final <- fit11 # LM
}


## Test of random effects
if(is.null(ranlist) == FALSE){
  ## Testing variance (RE)
  x2 <- as.numeric(-2 * logLik(fit11) + 2 * logLik(fit1))
  LRT_re <- 0.5 * (1 - pchisq(x2, df = 1))
  LRT_re
  if(LRT_re > 0.05){
    print("Random effect is not needed. Drop random term and refit")
  }
}

## AIC
aic_null <- AIC(logLik(fit11))


## Not systematic pattern implying assumption of homoscedaticity
pred <- predict(fit_final)
resd <- Data72$scoreme - pred


# fixed-effect
b.lme4 <- summary(fit_final)$coef[, 1]
b.sd.lme4 <- summary(fit_final)$coef[, 2]
```

© 2020 Asch DA et al. JAMA Internal Medicine.

```
dataplot_CM <- data.frame("Effect" = b.lme4,
                    "lb" = b.lme4 - 1.96 * b.sd.lme4,
                    "ub" = b.lme4 + 1.96 * b.sd.lme4,
                    "SE" = b.sd.lme4,
                    "Variable" = names(b.lme4),
                    "pv" = 2 * (1 - pnorm(abs(b.lme4/b.sd.lme4))))

dataplot_CM

vv <- varlist_bar
txt <- strsplit(vv, ":")[[1]]
## final name
txt2 <- paste0("mlist0_", tolower(txt), "", sep = "", collapse = ", ")
QQ <- paste0("c(", txt2,")")
QQ <-  eval(parse(text = QQ))


dataplot_CM$finalname <- c("Intercept", QQ)

## ylab_name
txt2 <- paste0("varlist_", tolower(txt), "", sep = "", collapse = ", ")
QQ <- paste0("c(", txt2,")")
QQ <- eval(parse(text = QQ))
dataplot_CM$ylab_name <- c("Intercept", QQ)

## reference
txt2 <- paste0("reflist_", tolower(txt), "", sep = "", collapse = ", ")
QQ <- paste0("c(", txt2,")")
QQ <- eval(parse(text = QQ))
dataplot_CM$reference <- c(NA, QQ)
```

© 2020 Asch DA et al. JAMA Internal Medicine.

```
dataplot_CM$allpoint <- unlist(lapply(seq_len(nrow(dataplot_CM)), function(hh)
  paste0("",sprintf("%5.2f",round(dataplot_CM$Effect[hh], 2))," (",sprintf("%5.2f",round(dataplot_CM$lb[hh], 2)),",",sprintf("%5.2f",round(dataplot_CM$ub[hh], 2)), ")")))

dataplot_CM$pv <- sprintf("%0.4f", as.numeric(dataplot_CM$pv))
dataplot_CM$pv[which(dataplot_CM$pv == "0.0000")] <- "<0.0001"

dataplot_CM <- dataplot_CM[-1, ]

## Obtain cases
dataplot_CM$ylab_name_numb <- t(do.call(cbind, lapply(seq_len(nrow(dataplot_CM)), function(uu) {

  where <- dataplot_CM$finalname[uu]
  where
  numb <- table(Data72[, colnames(Data72) %in% where])

  snumb <- sum(numb)

  if("1" %!in% names(numb)){ # RUN IFF VALUES 1/0 IS NOT USED FOR CATEGORY
    what <- strsplit(as.character(dataplot_CM$Variable[uu]), where)[[1]][2]
    numb <- numb[which(names(numb) == what)]
  } else{
    what <- as.character(1)
    numb <- numb[which(names(numb) == what)]
  }

  GG <- paste0("median(Data72$score[which(Data72$", where," == '", what,"')])")
  GG <- eval(parse(text = GG))
```

© 2020 Asch DA et al. JAMA Internal Medicine.

```
    as.vector(c(numb, round(((numb/snumb) * 100), 2), GG))
  }))) 



  output = list(vif = vif, pred = pred, resd = resd,
                Data72 = Data72, fit1 = fit1, fit11 = fit11, fit0 = fit0, fit00 = fit00,
                test_table1 = test_table1, test_table2 = test_table2, dataplot_CM = dataplot_CM,
                aic_alt = aic_alt, aic_null = aic_null)
}



# B4. Quintile comparison (model-based)
qunitile_compare <- function(Data_score, take_log = FALSE, glmlog = FALSE){

  q1 <- quantile(Data_score$score, 0.20)
  q2 <- quantile(Data_score$score, 0.40)
  q3 <- quantile(Data_score$score, 0.60)
  q4 <- quantile(Data_score$score, 0.80)

  iq1 <- which(Data_score$score <= q1)
  for(jj in 2 : 4){
    QQ <- paste0("iq",jj," <- which(Data_score$score > q",(jj-1), " & Data_score$score <= q",jj,")")
    QQ <- eval(parse(text = QQ))
  }
  iq5 <- which(Data_score$score > q4)

  id_q <- unlist(lapply(seq_len(5), function(kk){
    QQ <- paste0("rep(as.character('Q",kk,"'), length(iq", kk,"))")
    QQ <- eval(parse(text = QQ))
  }))
```

© 2020 Asch DA et al. JAMA Internal Medicine.

```
if(take_log == FALSE){
  dataq <- data.frame("score" = Data_score$score,
                    "group" = id_q,
                    "reference" = Data_score$reference)
} else if(take_log == TRUE){
  dataq <- data.frame("score" = log(Data_score$score),
                    "group" = id_q,
                    "reference" = Data_score$reference)
}

dataq$group <- relevel(dataq$group, ref = "Q1")

if(glmlog == FALSE){
  fit_expl <- lm(score ~ group, singular.ok = TRUE, data = dataq)
} else if(glmlog ==  TRUE){
  fit_expl <- glm(score ~ group, singular.ok = TRUE,
                family = gaussian(link = "log"),
                data = dataq)
}
summary(fit_expl)

# fixed-effect
b.lm <- summary(fit_expl)$coef[-1, 1]
b.sd.lm <- summary(fit_expl)$coef[-1, 2]

if(take_log == TRUE | glmlog == TRUE){
  datq <- data.frame("Effect" = exp(b.lm),
                   "lb" = exp(b.lm - 1.96 * b.sd.lm),
                   "ub" = exp(b.lm + 1.96 * b.sd.lm),
                   "SE" = b.sd.lm,
                   "Variable" = names(b.lm),
```

© 2020 Asch DA et al. JAMA Internal Medicine.

```
                "pv" = 2 * (1 - pnorm(abs(b.lm/b.sd.lm))))
  } else if(take_log == FALSE & glmlog == FALSE){
   datq <- data.frame("Effect" = b.lm,
                "lb" = b.lm - 1.96 * b.sd.lm,
                "ub" = b.lm + 1.96 * b.sd.lm,
                "SE" = b.sd.lm,
                "Variable" = names(b.lm),
                "pv" = 2 * (1 - pnorm(abs(b.lm/b.sd.lm))))
  }

  output = list(datq = datq, id_q = id_q)
}



# B5. Single GLMM for patient + hospital attributes
glmmfit_patient_hospital <- function(Data_analytical, hospital_fit_object, XXX_adj, add2, ranlist = paste0("(1 |
hosp_name)"),
                        ylist = "mbr_death_flag", variable_list = "Age:Gender:Nurse", comorb_nm, comorb_id){

  ## Getting data for hosp attributes
  XXX_adj2 <- c(XXX_adj, add2)

  Data23 <- Data_analytical; dim(Data23)
  uid2 <- unique(Data23$hosp_name); L <- length(uid2)

  Data23$beds_class <- Data23$icu_beds_class <- Data23$non_profit_flag <-
   Data23$hosp_usr_class <- Data23$academic_flag <- Data23$cases_class <-
   Data23$cases_class_early <- Data23$cases_class_late <-
   Data23$cases_class_late_diff <- Data23$cases_change <-
   Data23$cases_change_binary <- Data23$cases_interaction <-
```

© 2020 Asch DA et al. JAMA Internal Medicine.

```
  Data23$hosp_region <- rep(NA, nrow(Data23))

for(ll in 1 : L){
  rep_id <- which(Data23$hosp_name == uid2[ll])
  take_id <- which(hospital_fit_object$Data72$hosp_name == uid2[ll])
  Li <- length(rep_id)

  val_bed  <- hospital_fit_object$Data72$beds_class[take_id]
  val_icu <- hospital_fit_object$Data72$icu_beds_class[take_id]

  val_case <- hospital_fit_object$Data72$cases_class[take_id]
  val_case_early <- hospital_fit_object$Data72$cases_class_early[take_id]
  val_case_late <- hospital_fit_object$Data72$cases_class_late[take_id]
  val_case_late_diff <- hospital_fit_object$Data72$cases_class_late_diff[take_id]
  val_case_change <- hospital_fit_object$Data72$cases_change[take_id]
  val_case_change_binary <- hospital_fit_object$Data72$cases_change_binary[take_id]
  val_case_interaction <- hospital_fit_object$Data72$cases_interaction[take_id]

  val_pro <- hospital_fit_object$Data72$non_profit_flag[take_id]
  val_usr <- hospital_fit_object$Data72$hosp_usr_class[take_id]
  val_aca <- hospital_fit_object$Data72$academic_flag[take_id]
  val_reg <- hospital_fit_object$Data72$hosp_region[take_id]

  Data23$beds_class[rep_id] <- rep(as.character(val_bed), Li)
  Data23$icu_beds_class[rep_id] <- rep(as.character(val_icu), Li)
  Data23$non_profit_flag[rep_id] <- rep(as.character(val_pro), Li)
  Data23$hosp_usr_class[rep_id] <- rep(as.character(val_usr), Li)
  Data23$academic_flag[rep_id] <- rep(as.character(val_aca), Li)

  Data23$cases_class[rep_id] <- rep(as.character(val_case), Li)
  Data23$cases_class_early[rep_id] <- rep(as.character(val_case_early), Li)
```

© 2020 Asch DA et al. JAMA Internal Medicine.

```
  Data23$cases_class_late[rep_id] <- rep(as.character(val_case_late), Li)
  Data23$cases_class_late_diff[rep_id] <- rep(as.character(val_case_late_diff), Li)

  Data23$cases_change[rep_id] <- rep(as.character(val_case_change), Li)
  Data23$cases_change_binary[rep_id] <- rep(as.character(val_case_change_binary), Li)
  Data23$cases_interaction[rep_id] <- rep(as.character(val_case_interaction), Li)

  Data23$hosp_region[rep_id] <- rep(as.character(val_reg), Li)
}

## setting reference category
Data23 <- Data23 %>% mutate_if(is.character, as.factor)
Data23 <- within(Data23, icu_beds_class <- relevel(icu_beds_class, ref = "0 <= ICU < 20"))
Data23 <- within(Data23, beds_class <- relevel(beds_class, ref = "0 <= Beds < 150"))
Data23 <- within(Data23, academic_flag <- relevel(academic_flag, ref = "Academic"))
Data23 <- within(Data23, hosp_usr_class  <- relevel(hosp_usr_class, ref = "Rural"))
Data23 <- within(Data23, non_profit_flag  <- relevel(non_profit_flag, ref = "Profit"))
Data23 <- within(Data23, hosp_region  <- relevel(hosp_region, ref = "Central"))
Data23 <- within(Data23, cases_class_early  <- relevel(cases_class_early, ref = "0 <= Cases < 20"))
Data23 <- within(Data23, cases_interaction  <- relevel(cases_interaction, ref = "otherwise"))
Data23 <- within(Data23, cases_change_binary  <- relevel(cases_change_binary, ref = "Decrease"))
Data23 <- within(Data23, cases_class  <- relevel(cases_class, ref = "0 <= Cases < 20"))
Data23 <- within(Data23, cases_class_early  <- relevel(cases_class_early, ref = "0 <= Cases < 20"))
Data23 <- within(Data23, cases_class_late  <- relevel(cases_class_late, ref = "0 <= Cases < 20"))
Data23 <- within(Data23, cases_class_late_diff  <- relevel(cases_class_late_diff, ref = "0 <= Cases < 30"))
Data23 <- within(Data23, cases_change  <- relevel(cases_change, ref = "<0%"))

## Model fitting
gmft_hosp <- glmmfit(ylist, XXX_adj = XXX_adj2, ranlist, reml = TRUE, Data_analytical = Data23, addsize = TRUE, laplace
= FALSE)
summary(gmft_hosp$fom3)
```

© 2020 Asch DA et al. JAMA Internal Medicine.

```
lb_hosp <- gmft_hosp$lb; ub_hosp <- gmft_hosp$ub; sdorp_hosp <- gmft_hosp$sdorp;
or_hosp <- gmft_hosp$or; orp_hosp <- gmft_hosp$orp;
dat_hosp <- data.frame("Variable" = names(or_hosp),
            "bOR" =  or_hosp,
            "lb" =  lb_hosp,
            "ub" = ub_hosp,
            "pval" = 2 * (1 - pnorm(abs(orp_hosp/sdorp_hosp))))
symbol <- "\u2265"; symbol2 <- intToUtf8(8804)

mlist_age <- c("age_class"); varlist_age <- c("Age::[45,55)", "Age::[55,65)",
                        "Age::[65,75)", "Age::[75,85)", paste0("Age::", symbol,"85"));
reflist_age <- rep("[18,45)", length(varlist_age)); mlist0_age <- rep("age_class", length(varlist_age))

mlist_gender <- c("gender"); varlist_gender <- c("Gender::Male"); reflist_gender <- rep("Female",
length(varlist_gender));
mlist0_gender <- rep("gender", length(varlist_gender))
mlist_elix <- c("elix_class"); varlist_elix <- c("Elixhauser::[1,10)", "Elixhauser::[10,20)",
                         paste0("Elixhauser::", symbol,"20"));
reflist_elix <- rep("[0,1)", length(varlist_elix)); mlist0_elix <- rep("elix_class", length(varlist_elix))

mlist_comorbidity <- c(comorb_id); varlist_comorbidity <- paste0("", comorb_nm, "::Yes", sep = "");
reflist_comorbidity <- rep("No", length(varlist_comorbidity)); mlist0_comorbidity <- comorb_id
mlist_race <- c("race_cd_class"); varlist_race <- c("Race::African-american", "Race::Other");
reflist_race <- rep("Caucasian", length(varlist_race)); mlist0_race <- rep("race_cd_class", length(varlist_race))

mlist_nurse <- c("snf_nursing_flag"); varlist_nurse <- c("Transferred from nursing facility::Yes");
reflist_nurse <- rep("No", length(varlist_nurse)); mlist0_nurse <- rep("snf_nursing_flag", length(varlist_nurse))
mlist_lob <- c("lob"); varlist_lob <- c("LOB::Medicare"); reflist_lob <- "Commercial"; mlist0_lob <- "lob"

mlist_count <- c("count_day_class"); varlist_count <- c("Days from January 1,2020::[0,90)",
```

© 2020 Asch DA et al. JAMA Internal Medicine.

```
                                        "Days from January 1,2020::[90,120)", "Days from January 1,2020::[120,150)")
reflist_count <- rep(paste0("", symbol,"150"), length(varlist_count));
mlist0_count <- rep("count_day_class", length(varlist_count))

mlist_numeric <- c("count_day", "count_day2"); varlist_numeric <- c("Days", "Days^2");
reflist_numeric <- rep(NA, length(mlist_numeric)); mlist0_numeric <- c("count_day", "count_day2")

mlist_size <- c("size"); varlist_size <- c("Log(volume)"); reflist_size <- NA; mlist0_size <- c("size")

## Hospital levels variables
# user
mlist_user <- c("hosp_usr_class"); varlist_user <- c("Location::Urban");
reflist_user <- rep("Rural", length(varlist_user)); mlist0_user <- rep("hosp_usr_class", length(varlist_user))

# ratio
mlist_ratio <- c("resident_to_bed_ratio_class"); varlist_ratio <- c("Resident to bed ratio::[0.05,0.25)",
                                              paste0("Resident to bed ratio::", symbol,"0.25"));
reflist_ratio <- rep("[0,0.05)", length(varlist_ratio)); mlist0_ratio <- rep("resident_to_bed_ratio_class",
                                                   length(varlist_ratio))

# ICU
mlist_icu <- c("icu_beds_class"); varlist_icu <- c("Number of ICU beds::[20,60)",
                                    paste0("Number of ICU beds::", symbol,"60"));
reflist_icu <- rep("[0,20)", length(varlist_icu)); mlist0_icu <- rep("icu_beds_class", length(varlist_icu))

# region
mlist_region <- c("hosp_region"); varlist_region <- c(
  "Region::North-east",
  "Region::South-east",
  "Region::West");
reflist_region <- rep("Central", length(varlist_region)); mlist0_region <- rep("hosp_region", length(varlist_region))
```

© 2020 Asch DA et al. JAMA Internal Medicine.

```
# beds
mlist_beds <- c("beds_class"); varlist_beds <- c("Number of beds:[150,300)",
                                "Number of beds:[300,450)",
                                paste0("Number of beds::", symbol,"450"));
reflist_beds <- rep("[0,150)", length(varlist_beds)); mlist0_beds <- rep("beds_class", length(varlist_beds))

# academic
mlist_academic <- c("academic_flag"); varlist_academic <- c("Hospital affiliation::Non-academic");
reflist_academic <- rep("Academic", length(varlist_academic));
mlist0_academic <- rep("academic_flag", length(varlist_academic))

# profit
mlist_profit <- c("non_profit_flag"); varlist_profit <- c("Profit status::Non-profit", "Profit status::Other");
reflist_profit <- rep("For profit", length(varlist_profit));
mlist0_profit <- rep("non_profit_flag", length(varlist_profit))

# cases
mlist_cases <- c("cases_class"); varlist_cases <- c("Cases per 10K:[20,40)",
                                  paste0("Cases per 10K::", symbol,"40"));
reflist_cases <- rep("[0,20)", length(varlist_cases)); mlist0_cases <- rep("cases_class", length(varlist_cases))


# cases
mlist_cases <- c("cases_class"); varlist_cases <- c("Cases per 10K::[20,40)",
                                  paste0("Cases per 10K::", symbol,"40"));
reflist_cases <- rep("[0,20)", length(varlist_cases)); mlist0_cases <- rep("cases_class", length(varlist_cases))


# cases early
mlist_casesearly <- c("cases_class_early"); varlist_casesearly <- c("Cases (early) per 10K::[20,40)",
```

© 2020 Asch DA et al. JAMA Internal Medicine.

```
                                   paste0("Cases (early) per 10K::", symbol,"40"));
  reflist_casesearly <- rep("[0,20)", length(varlist_casesearly)); mlist0_casesearly <- rep("cases_class_early",
length(varlist_casesearly))

  # cases late
  mlist_caseslate <- c("cases_class_late"); varlist_caseslate <- c("Cases (late) per 10K::[20,40)",
                                   paste0("Cases (late) per 10K::", symbol,"40"));
  reflist_caseslate <- rep("[0,20)", length(varlist_caseslate)); mlist0_caseslate <- rep("cases_class_late",
length(varlist_caseslate))

  # cases change
  mlist_caseschange <- c("cases_change"); varlist_caseschange <- c("Cases change %::[0,100)", "Cases change
%::[100,250)",
                                   paste0("Cases change %::", symbol,"250"));
  reflist_caseschange <- rep("<0%", length(varlist_caseschange)); mlist0_caseschange <- rep("cases_change",
length(varlist_caseschange))


  # cases change binary
  mlist_caseschangebinary <- c("cases_change_binary"); varlist_caseschangebinary <- c("Cases change::Increase");
  reflist_caseschangebinary <- rep("Decrease", length(varlist_caseschangebinary));
  mlist0_caseschangebinary <- rep("cases_change_binary", length(varlist_caseschangebinary))


  # cases interaction
  mlist_casesinteraction <- c("cases_interaction");
  varlist_casesinteraction <- c("Cases::[20,40) & Increase", paste0("Cases::", symbol,"40 & Increase"));
  reflist_casesinteraction <- rep("otherwise", length(varlist_casesinteraction));
  mlist0_casesinteraction <- rep("cases_interaction", length(varlist_casesinteraction))

  # cases late
  mlist_caseslatediff <- c("cases_class_late_diff");
```

© 2020 Asch DA et al. JAMA Internal Medicine.

```
varlist_caseslatediff <- c("Cases (late) per 10K::[30,60)", "Cases (late) per 10K::[60,90)",
                          paste0("Cases (late) per 10K::", symbol,"90"));
reflist_caseslatediff <- rep("[0,30)", length(varlist_caseslatediff));
mlist0_caseslatediff <- rep("cases_class_late_diff", length(varlist_caseslatediff))

# deaths
mlist_deaths <- c("cases_class"); varlist_deaths <- c("Deaths per 10K:[1,3)", "Deaths per 10K:[3,5)",
                                                     paste0("Deaths per 10K::", symbol,"5"));
reflist_deaths <- rep("[0,1)", length(varlist_deaths)); mlist0_deaths <- rep("deaths_class", length(varlist_deaths))



## Rearranging dat for coefficients
txt <- strsplit(variable_list, ":")[[1]]
## final name
txt2 <- paste0("mlist0_", tolower(txt), "", sep = "", collapse = ", ")
QQ <- paste0("c(", txt2,")")
QQ <-  eval(parse(text = QQ))



inout_ix <- unlist(lapply(seq_len(length(QQ)), function(ii) {
  inout <- grep(QQ[ii], dat_hosp$Variable, value = F)
  if(length(inout) >= 1){
    1
  } else{
    9999 # missing
  }
}))

lni <- which(inout_ix == 9999)
if(length(lni) > 0){
  dat_hosp$finalname <-  QQ[- which(inout_ix == 9999)]
```

© 2020 Asch DA et al. JAMA Internal Medicine.

```
} else{
  dat_hosp$finalname <- QQ
}

## ylab_name
txt2 <- paste0("varlist_", tolower(txt), "", sep = "", collapse = ", ")
QQ <- paste0("c(", txt2,")")
QQ <- eval(parse(text = QQ))

if(length(lni) > 0){
  dat_hosp$ylab_name <-  QQ[- which(inout_ix == 9999)]
} else{
  dat_hosp$ylab_name <- QQ
}
##reference
txt2 <- paste0("reflist_", tolower(txt), "", sep = "", collapse = ", ")
QQ <- paste0("c(", txt2,")")
QQ <- eval(parse(text = QQ))

if(length(lni) > 0){
  dat_hosp$reference <- QQ[- which(inout_ix == 9999)]
} else{
  dat_hosp$reference <- QQ
}
name_coef <- as.character(dat_hosp$Variable)

##Obtain cases
ylab_name_numb <- t(do.call(cbind, lapply(seq_len(nrow(dat_hosp)), function(uu) {

  where <- dat_hosp$finalname[uu]
  where
```

© 2020 Asch DA et al. JAMA Internal Medicine.

```
    numb <- table(Data23[, colnames(Data23) %in% where])
    what <- strsplit(name_coef[uu], where)[[1]][2]
    numb <- numb[which(names(numb) == what)]

    QQ <- subset(Data23, select = c(where, ylist))

    numb_event <- length(which(QQ[, 1] == what & QQ[, 2] == "Event"))

    as.vector(c(numb, numb_event))
  })))

  ## Merging all
  dat_hosp$ylab_name_numb <- ylab_name_numb[, 1]
  dat_hosp$ylab_name_numb_event <- ylab_name_numb[, 2]
  dat_hosp$allpoint <- unlist(lapply(seq_len(nrow(dat_hosp)), function(hh)
    paste0("",sprintf("%2.2f",round(dat_hosp$bOR[hh], 2)),"(",sprintf("%2.2f",round(dat_hosp$lb[hh],
2)),",",sprintf("%2.2f",round(dat_hosp$ub[hh], 2)), ")")))

  dat_hosp$ylab_name_numbX = paste0("", dat_hosp$ylab_name_numb_event, "/", dat_hosp$ylab_name_numb, "")

  zerome <- which(dat_hosp$ylab_name_numbX == "0/0")
  if(length(zerome) > 0){
    dat_hosp$ylab_name_numbX[zerome] <- NA
  }

  dat_hosp$pval <- sprintf("%0.4f", dat_hosp$pval)
  dat_hosp$pval[which(dat_hosp$pval == "0.0000")] <- "<0.0001"

  output = list(dat_hosp = dat_hosp, gmft_hosp = gmft_hosp)
}
```

© 2020 Asch DA et al. JAMA Internal Medicine.

```
# Internal function in Hmisc package for somer's C /Dxy statistic
.somers2 = function (x, y, weights = NULL, normwt = FALSE, na.rm = TRUE)
{
  if (length(y) != length(x))
    stop("y must have same length as x")
  y <- as.integer(y)
  wtpres <- length(weights)
  if (wtpres && (wtpres != length(x)))
    stop("weights must have same length as x")
  if (na.rm) {
    miss <- if (wtpres)
      is.na(x + y + weights)
    else is.na(x + y)
    nmiss <- sum(miss)
    if (nmiss > 0) {
      miss <- !miss
      x <- x[miss]
      y <- y[miss]
      if (wtpres)
        weights <- weights[miss]
    }
  }
  else nmiss <- 0
  u <- sort(unique(y))
  if (any(!(y %in% 0:1) ))
    stop("y must be binary")
  if (wtpres) {
    if (normwt)
      weights <- length(x) * weights/sum(weights)
    n <- sum(weights)
```

© 2020 Asch DA et al. JAMA Internal Medicine.

```
  }
  else n <- length(x)
  if (n < 2)
    stop("must have >=2 non-missing observations")
  n1 <- if (wtpres)
    sum(weights[y == 1])
  else sum(y == 1)
  if (n1 == 0 || n1 == n)
    return(c(C = NA, Dxy = NA, n = n, Missing = nmiss))
  mean.rank <- if (wtpres){
    require(Hmisc,quietly=TRUE)
    wtd.mean(wtd.rank(x, weights, na.rm = FALSE), weights *
             y)
  } else mean(rank(x)[y == 1])
  c.index <- (mean.rank - (n1 + 1)/2)/(n - n1)
  dxy <- 2 * (c.index - 0.5)
  r <- c(c.index, dxy, n, nmiss)
  names(r) <- c("C", "Dxy", "n", "Missing")
  r
}



# C1. Forest plot of odds ratio
plotOR_paper <- function(dataplot, xl = 0.2, xu = 5,
                         title = "Main title",
                         txtcol = paste0("No. events/\nCOVID patients"),
                         xlab_title = "Adjusted odd"){
  lc <- nrow(dataplot)
  revy <- seq_len(lc)
  plot(x = dataplot$bOR, y = rev(seq_len(lc)),
       xlab = xlab_title, ylab = '', xaxt = "n",
```

© 2020 Asch DA et al. JAMA Internal Medicine.

```
    yaxt = "n", pch = NA, log = "x",
    lwd = 2, cex.lab = 1.5, cex.main = 2,
    cex = 2, cex.axis = 2,
    xlim = c(xl, xu))

atx <- axTicks(1)
axis(1, at = atx, labels = atx, cex.axis = 1.7)
ven_tex_lb <- xl;
ven_tex_ub <- 0.9;
ven_tex_lb1 <- 1.1;
ven_tex_ub1 <- xu

ven_tex <- nrow(dataplot) + 1.5

RR <- as.character(dataplot$reference)
dataplot$reference <- RR

# Var names
mtext(side = 2, dataplot$ylab_name,
     at = rev(revy), line = 7, las = 2,
     cex = 1, font = 1)

# Reference names
mtext(side = 2, StrAlign(dataplot$reference, sep = "\\r"), at = rev(revy), las = 1, cex = 1,
     font = 1, line = ifelse(txtcol == "No. events/\nCOVID patients", 2, 3), col = "black")

# CI values
mtext(side = 4, StrAlign(dataplot$allpoint, sep = "\\r"),
     at = rev(revy),
     las = 2, cex = 0.8, font = 1)
```

© 2020 Asch DA et al. JAMA Internal Medicine.

```
# P values
col_pv <- ifelse(dataplot$pval <= 0.05, "orange",
              ifelse(dataplot$pval > 0.05 & dataplot$pval <= 0.10, "purple", "black"))

mtext(side = 4, StrAlign(as.character(dataplot$pval), sep = "\\r"),
    at = rev(revy), col = "black",
    las = 2, line = 7, cex = 0.8, font = 2)

# Events
mtext(side = 2, dataplot$ylab_name_numbX, at = rev(revy), las = 2,
    line = 25, cex = 0.8, font = 1, col = "black")



# Column names
mtext(side = 2, txtcol, at = (ven_tex + 1),
    line = 23, las = 1, cex = 1,
    font = 2, col = "black")

mtext(side = 2, paste0("Characteristic better"), at = (ven_tex + 1),
    line = -5,
    las = 1, cex = 0.8, lwd = 1.2,
    font = 3,
    col = "black")

mtext(side = 2, paste0("Characteristic worse"), at = (ven_tex + 1),
    line = -15,
    las = 1, cex = 0.8, lwd = 1.2,
    font = 3, col = "black")

mtext(side = 4, title, at = (ven_tex + 2),
    line = -43,
```

© 2020 Asch DA et al. JAMA Internal Medicine.

```
     las = 1, cex = 1.5, lwd = 1.2,
     font = 21, col = "black")


mtext(side = 2, paste0("Reference"), at = (ven_tex + 1),
     line = 2,
     las = 1, cex = 1, lwd = 1.2,
     font = 2, col = "black")

mtext(side = 2, paste0("Characteristic"), at = (ven_tex + 1),
     line = 9,
     las = 1, cex = 1, lwd = 1.2,
     font = 2, col = "black")

mtext(side = 4, paste0("Estimate (95% CI)"),
     at = (ven_tex + 1),
     line = -1,
     las = 1, cex = 1, lwd = 1.2,
     font = 2, col = "black")

mtext(side = 4, paste0("p-value"),
     at = (ven_tex + 1),
     line = 7,
     las = 1, cex = 1, lwd = 1.2,
     font = 2, col = "black")


segments(x0 = xl, x1 = xu, y0 = revy, y1 = revy,
       col = "#EEEEEE", lty = 1, lwd = 2)
segments(x0 = 1, x1 = 1, y0 = 0, y1 = tail(revy, 1) + 1,
       col = "black", lty = 3, lwd = 2)
```

© 2020 Asch DA et al. JAMA Internal Medicine.

```
  segments(round(dataplot$lb, 2),
           rev(revy), round(dataplot$ub, 2),
           rev(revy),
           col = "black", lty = 1, lwd = 2)

  arrows(ven_tex_lb, (ven_tex -
                        ifelse(txtcol == "No. events/\nCOVID patients", 0.7, 1)),
         ven_tex_ub, (ven_tex -
                        ifelse(txtcol == "No. events/\nCOVID patients", 0.7, 1)), col = "black",
         lwd = 1, code = 1, lty = 5, length = 0.10)
  arrows(ven_tex_lb1, (ven_tex -
                         ifelse(txtcol == "No. events/\nCOVID patients", 0.7, 1)),
         ven_tex_ub1, (ven_tex -
                         ifelse(txtcol == "No. events/\nCOVID patients", 0.7, 1)), col = "black",
         lwd = 1, code = 2, lty = 5, length = 0.10)

  points(dataplot$bOR, rev(seq_len(lc)),
         xlab = '', lwd = 2,
         col = "black",
         cex = 1.5,
         pch = 3)
}

## C2. RSER/RSMR line plot
plotScore_paper_single_rotate <- function(dataplot,
                                          dataplot2 = NULL,
                                          xl = 0,
                                          xu = 0.30,
                                          maxX = 1000,
                                          sec_xl = -0.15,
```

© 2020 Asch DA et al. JAMA Internal Medicine.

```
                    sec_xu = 0.75,
                    atx1 = c(1, 200, 400, 600, 800, 960),
                    type = "early",
                    plot_unadj = TRUE,
                    ybar_early = ybar_early,
                    ybar_later = ybar_later,
                    ybar_orig,
                    confidence = FALSE,
                    title = "Hospital-specific standardized risk score",
                    ylab_title = "Standardized score",
                    xlab_title = "Hospital rank",
                    legend_title = "event"){
lc <- nrow(dataplot)
revy <- seq_len(lc)


if(is.null(type) == TRUE){
  QQ <- dataplot$score
} else if(is.null(type) == FALSE & type != "both"){
  QQ <- paste0("dataplot$", type,"_score")
  QQ <- eval(parse(text = QQ))
} else if(type == "both"){
  QQ1 <- paste0("dataplot$early_score")
  QQ1 <- eval(parse(text = QQ1))

  QQ <- QQ1
  QQ2 <- paste0("dataplot2$later_score")
  QQ2 <- eval(parse(text = QQ2))
}
```

© 2020 Asch DA et al. JAMA Internal Medicine.

```
plot(y = QQ,
    x = rev(seq_len(lc)),
    main = '',
    xlim = c(1, maxX),
    xlab = xlab_title,
    ylab = '', xaxt = "n",
    yaxt = "n", pch = NA, #log = "x",
    lwd = 2,
    cex.lab = 1.8,
    cex = 1.8,
    cex.axis = 1.8,
    ylim = c(xl, xu))

atx <- seq(xl, xu, length.out = 6)

# bottom
axis(2, at = atx, line = 0, col = "black",
    lwd = 2, labels = atx, cex.axis = 1.8)

axis(1, at = atx1, line = 0, col = "black",
    lwd = 2, labels = atx1, cex.axis = 1.8)

# Unadj score
mtext(side = 2, ylab_title, line = 2, font = 21,
     lwd = 1.2, cex = 1.8, las = 3)



## Orginal
segments(y0 = ybar_orig, y1 = ybar_orig,
        x0 = -1, x1 = lc,
        col = "black",
```

© 2020 Asch DA et al. JAMA Internal Medicine.

```
      lty = 2, lwd = 2)

if(is.null(type) == FALSE){
  if(type == "early"){
    # early
    segments(y0 = ybar_early, y1 = ybar_early,
             x0 = -1, x1 = lc,
             col = "black",
             lty = 3, lwd = 1.8)
  }
}
if(is.null(type) == FALSE){
  if(type == "later"){
    # later
    segments(y0 = ybar_later, y1 = ybar_later,
             x0 = -1, x1 = lc,
             col = "black",
             lty = 6, lwd = 1.8)
  }
}

symbol <- "\u2265"; symbol2 <- intToUtf8(8804)

compare_ybar <- ifelse(is.null(type) == TRUE,
                    ybar_orig,
                    ifelse(type == "early",
                       ybar_early, ybar_later))

if(length(type %in% c("early", "both", "later")) != 0){

  points(y = rev(QQ),
```

© 2020 Asch DA et al. JAMA Internal Medicine.

```
    x = rev(seq_len(lc)),
    xlab = '', lwd = 0.8,
    col = ifelse(rev(QQ > compare_ybar),
           "red", "blue"),
    cex = 0.4, pch = ifelse(type == "early", 3, 23))

if(confidence == "SE mean"){
  polygon(x = c(seq_len(lc), rev(seq_len(lc))),
      y = c(dataplot$score_boot_mean_lb,
         rev(dataplot$score_boot_mean_ub)),
      col =  adjustcolor("black", alpha.f = 0.40),
      border = NA)
}else if(confidence == "IQR"){
  polygon(x = c(seq_len(lc), rev(seq_len(lc))),
      y = c(dataplot$score_boot_q1,
         rev(dataplot$score_boot_q3)),
      col =  adjustcolor("gray", alpha.f = 0.40),
      border = NA)
} else if(confidence == "Yale 95%"){
  polygon(x = c(seq_len(lc), rev(seq_len(lc))),
      y = c(dataplot$score_boot_025,
         rev(dataplot$score_boot_975)),
      col =  adjustcolor("orange", alpha.f = 0.20),
      border = NA)
}

points(y = rev(QQ),
    x = rev(seq_len(lc)),
    xlab = '', lwd = 0.8,
    col = ifelse(rev(QQ > compare_ybar),
           "red", "blue"),
```

© 2020 Asch DA et al. JAMA Internal Medicine.

```
       cex = 0.4, pch = ifelse(type == "early", 3, 23))

}



if(is.null(type) == TRUE){

  points(y = rev(QQ),
       x = rev(seq_len(lc)),
       xlab = '', lwd = 0.8,
       col = ifelse(rev(QQ > compare_ybar),
                    "red", "blue"),
       cex = 0.4, pch = 3)
  if(confidence == "SE mean"){
   polygon(x = c(seq_len(lc), rev(seq_len(lc))),
          y = c(dataplot$score_boot_mean_lb,
               rev(dataplot$score_boot_mean_ub)),
          col =  adjustcolor("black", alpha.f = 0.40),
          border = NA)
  }else if(confidence == "IQR"){
   polygon(x = c(seq_len(lc), rev(seq_len(lc))),
          y = c(dataplot$score_boot_q1,
               rev(dataplot$score_boot_q3)),
          col =  adjustcolor("gray", alpha.f = 0.40),
          border = NA)
  } else if(confidence == "Yale 95%"){
   polygon(x = c(seq_len(lc), rev(seq_len(lc))),
          y = c(dataplot$score_boot_025,
               rev(dataplot$score_boot_975)),
          col =  adjustcolor("orange", alpha.f = 0.20),
          border = NA)
```

© 2020 Asch DA et al. JAMA Internal Medicine.

```
  }
  points(y = rev(QQ),
         x = rev(seq_len(lc)),
         xlab = '', lwd = 0.6,
         col = ifelse(rev(QQ > compare_ybar),
                      "red", "blue"),
         cex = 0.4, pch = 3)

}



if(plot_unadj == TRUE){
  ## secondary axes
  par(new = TRUE)  # Add new plot
  ## secondary
  if(is.null(type) == TRUE){
    EE <- sprintf("%0.3f", as.numeric(dataplot$unadj_score))
    EE <- as.numeric(EE)
  } else{
    EE <- paste0("dataplot$", type,"_unadj_score")
    EE <- eval(parse(text = EE))
  }

  dataplot$diff_u <- EE - QQ
  summary(dataplot$diff_u)



  plot(y = dataplot$diff_u,
       x = rev(seq_len(lc)),
       main = '',
       xlim = c(1, maxX),
```

© 2020 Asch DA et al. JAMA Internal Medicine.

```
     xlab = '',
     ylab = '',
     xaxt = "n",
     yaxt = "n",
     pch = NA,
     lwd = 2,
     cex.lab = 2,
     cex = 2,
     cex.axis = 2,
     ylim = c(sec_xl, sec_xu))
atx4 <- seq(sec_xl, sec_xu, by = 0.15)


axis(4, at = atx4,
     line = 0,
     col = "black",
     lwd = 2,
     labels = atx4,
     cex.axis = 1.8)


mtext(side = 4, "Rate differences (unadjusted - standardized)",
      line = 2, font = 21,
      lwd = 1.2, cex = 2)

points(y = rev(dataplot$diff_u),
       x = rev(seq_len(lc)),
       xlab = '', lwd = 1.2,
       col = adjustcolor("gray2", alpha.f = 0.30),
       cex = 1,
       pch = 17)
```

© 2020 Asch DA et al. JAMA Internal Medicine.

```
} # unadj plot ends

# legend
if(plot_unadj == TRUE & is.null(type) == TRUE){

  legend('topleft', bty = "n", cex = 1.4,
         pch = c(NA, 17),
         lwd = c(3, NA),
         lty = c(2),
         col = c("gray2", "black"),
         horiz = F,
         c(paste0("Observed ", legend_title, " rate"),
           paste0("Unadjusted ", legend_title, " rate")))
} else if(plot_unadj == FALSE & is.null(type) == TRUE){


  legend('topleft', bty = "n", cex = 1.2,
         pch = c(NA),
         lwd = c(2),
         lty = c(2),
         col = c("black"),
         horiz = F,
         paste0("Observed ", legend_title, " rate"))

} else if(plot_unadj == FALSE & is.null(type) == FALSE & type != "both"){

  if(type == "early"){
    legend('topleft', bty = "n", cex = 1.2,
           pch = c(NA, NA),
           lwd = c(3, 3),
```

© 2020 Asch DA et al. JAMA Internal Medicine.

```
        lty = c(2, 3),
        col = c("black", "black"),
        horiz = F,
        c(paste0("Observed ", legend_title, " rate"),
          paste0("Observed ", legend_title, " rate in early period (January 1 - April 30, 2020)")))
  }
  if(type == "later"){
    legend('topleft', bty = "n", cex = 1.2,
        pch = c(NA),
        lwd = c(3),
        lty = c(6),
        col = c("black"),
        horiz = F,
        paste0("Observed ", legend_title, " rate in late period (May 1 - June 30, 2020)"))
  }



} else if(plot_unadj == FALSE & is.null(type) == FALSE & type == "both"){


  legend('topleft', bty = "n", cex = 1.2,
       pch = c(NA, NA, NA),
       lwd = c(1.5, 1.5, 1.5),
       lty = c(2, 3, 6),
       col = c("black", "black", "black"),
       horiz = F,
       c(paste0("Observed ", legend_title, " rate"),
         paste0("Observed ", legend_title, " rate in early period (January 1 - April 30, 2020)"),
         paste0("Observed ", legend_title, " rate in late period (May 1 - June 30, 2020)")))
}
```

© 2020 Asch DA et al. JAMA Internal Medicine.

```
}



# C3. Bland-Altman
bland_altman_all <- function(s1, s2,
                        score_early2j,
                        score_later2j,
                        ylm_l = -25, ylm_u = 10, xlm_l = 0, xlm_u = 40,
                        legend_title = "event",
                        region_com, col_region,
                        slope = c(-2/3, -2/7, 2/9),
                        N1 = c(286, 111, 1, 0)){
  par(pty = "s")
  plot(x = s2,
      y = s1,
      cex = 0.9,
      ylab = paste0('Absolute change in risk standardized\n', legend_title, ' rates (Late - Early) %'),
      xlab = paste0('\n\n\n\n\nAverage of early and late risk \nstandardized ', legend_title,' rates (%)'),
      pch = 20,
      col = adjustcolor("gray4", alpha.f = 0.10),
      lwd = 2, cex.lab = 1.3,
      type = "p", axes = FALSE,
      xlim = c(xlm_l, xlm_u),
      ylim = c(ylm_l, ylm_u))


  abline(h = 0, lwd = 2, lty = 5)
  axis(2, at = c(-25, -20, -15, -10, -5, 0, 5, 10),
      labels = sprintf("%2.0f", c(-25, -20, -15, -10, -5, 0, 5, 10)),
      lwd = 2, cex.axis = 1.4)
```

© 2020 Asch DA et al. JAMA Internal Medicine.

```
axis(1, at = seq(xlm_l, xlm_u, length.out = 5),
     labels = seq(xlm_l, xlm_u, length.out = 5), lwd = 2,
     cex.axis = 1.4)



if(is.null(slope) == TRUE){
  segments(x0 = xlm_l, x1 = xlm_u, y0 = 0, y1 = ylm_l,
           col = "black", lty = 5, lwd = 2)
} else{

  polygon(x = c(xlm_l, xlm_u, xlm_u, xlm_l),
          y = c(ylm_l, ylm_l, 0, 0),
          col = adjustcolor("red", alpha.f = 0.10),
          border = adjustcolor("red", alpha.f = 0.2),
          lwd = 1.5,
          lty = c("solid"))

  nr <- ifelse(slope < 0, abs(ylm_l) / abs(slope),
             ylm_u / slope)

  for(uu in 1 : length(nr)){
    if(uu == 1){
      polygon(x = c(xlm_l, xlm_l, nr[uu]),
              y = c(0, ylm_l, ylm_l),
              col = adjustcolor("green", alpha.f = 0.10),
              border = adjustcolor("green", alpha.f = 0.20),
              lwd = 1.5,
              lty = c("solid"))
    }else if(uu == 2){
      polygon(x = c(xlm_l, xlm_u, xlm_u, nr[1]),
              y = c(0, (slope[2] * nr[1]), ylm_l, ylm_l),
```

© 2020 Asch DA et al. JAMA Internal Medicine.

```
            col = adjustcolor("yellow", alpha.f = 0.10),
            border = adjustcolor("yellow", alpha.f = 0.20),
            lwd = 1.5,
            lty = c("solid"))
    } else if(uu == 3){
      polygon(x = c(xlm_l, xlm_u, xlm_u),
            y = c(0, 0, (xlm_u * slope[uu])),
            col = adjustcolor("purple", alpha.f = 0.10),
            border = adjustcolor("purple", alpha.f = 0.20),
            lwd = 1.5,
            lty = c("solid"))
    }
  }
}

## re-adding points
points(x = s2,
      y = s1,
      cex = 0.9,
      pch = 20,
      col = adjustcolor("gray4", alpha.f = 0.10),
      lwd = 2)



symbol <- "\u2265"; symbol2 <- intToUtf8(8804)
mtext(side = 1,
     StrAlign(paste0("n = ",N1[1],""), sep = "\\l"),
     at = 5,
     las = 1, cex = 1,
     font = 22,
```

© 2020 Asch DA et al. JAMA Internal Medicine.

```
      line = -5, col = "black")
  mtext(side = 1, StrAlign(paste0("n = ",N1[2],""), sep = "\\l"),
      at = 35, las = 1, cex = 1, font = 22,
      line = -7, col = "black")
  mtext(side = 1, StrAlign(paste0("n = ",N1[3],""), sep = "\\l"),
      at = 35,
      las = 1, cex = 1,
      font = 22,
      line = -12, col = "black")
  mtext(side = 1, StrAlign(paste0("n = ",N1[4],""), sep = "\\l"),
      at = 35,
      las = 1, cex = 1,
      font = 22,
      line = -17, col = "black")

  # legend
  legend("topleft", bty = "n", cex = 0.8,
       lwd = 7,
       x.intersp = 1,
       y.intersp = 1,
       col = c(adjustcolor("green", alpha.f = 0.50),
             adjustcolor("yellow", alpha.f = 0.50),
             adjustcolor("red", alpha.f = 0.50),
             adjustcolor("purple", alpha.f = 0.50)),
       horiz = F,
       c("50% or more relative reduction",
         "[25-50)% reduction",
         "[0,25)% relative reduction",
         "(0,25]% relative increment"))
}
```

© 2020 Asch DA et al. JAMA Internal Medicine.

```
# C4. Find summary statistics of a vector
summary_statistics_table <- function(score2find){
  stat <- stat.desc(score2find, basic = TRUE)
  stat <- c(stat, quantile(score2find, 0.75) -
            quantile(score2find, 0.25))

  result <- as.matrix(stat)

  rownames(result) <- c(head(rownames(result), (length(stat) - 1)), "IQR")#,


  result <- result[rownames(result) %in% c("nbr.val", "min", "max",
                                    "median", "mean",
                                    "SE.mean",
                                    "var",
                                    "IQR"), ]

  result <- as.matrix(result)
  rownames(result) <- c("Number", "Minimum", "Maximum", "Median", "Mean",
                    "SE of mean",
                    "Variance", "IQR")
  colnames(result) <- "Summary statistics"
  result <- as.data.frame(round(result, 3))

  cimean <- paste0("(", sprintf("%0.2f", mean(score2find) - 1.96 * sd(score2find) /
                             sqrt(length(score2find))), ", ",sprintf("%0.2f", mean(score2find) + 1.96 * sd(score2find) /
                                                   sqrt(length(score2find))),")")

  result$`Summary statistics` <- sprintf("%0.2f", result$`Summary statistics`)
```

© 2020 Asch DA et al. JAMA Internal Medicine.

```
  result2 <- c(result$`Summary statistics`, cimean)
  result2 <- as.matrix(result2)
  rownames(result2) <- c(rownames(result), "95% CI for mean")
  colnames(result2) <- "Summary statistics"
  grid.table(as.data.frame(result2))
}


# c5. Generate bi-variate boxplots
Compare_numeric_boxplot_ggplot <- function(Data_score_earlylate, ylab_title = "Risk standardized event rates") {

  x_pos_cat <- c(0.75, 1.25, 1.85, 2.25)
  x_pos_num <- c(1, 2)

  # Draw plot
  p <- ggplot(data = Data_score_earlylate, aes_string(x = "Period", y = "Score")) +
    # Drawing boxplot
    geom_boxplot(colour = "gray47", fill = c("burlywood", "aquamarine"),
                 notch = FALSE, show.legend = FALSE, outlier.shape = NA) +
    # Giving title
    # Finding median and confidence band for median
    stat_summary(fun.data = f_median, geom = "crossbar",
                 colour = NA, fill = "lemonchiffon3", width = 0.8, alpha = 0.8) +
    # Jitter
    geom_jitter(position = position_jitter(width = 0.08, height = 0.01, seed = 521)) +

    theme_minimal() +

    # changing lable/axes size
    theme(
      axis.text.x = element_text(size = 14),
```

© 2020 Asch DA et al. JAMA Internal Medicine.

```
    axis.text.y = element_text(size = 14),
    axis.title.y = element_text(size = 14),
    axis.title.x = element_text(size = 14),
    axis.title = element_text(size = 14),
    legend.text = element_text(size = 14)) +

   labs(x = "") + labs(y = ylab_title) +
   # Displaying mean
   stat_summary(fun.y = mean, geom = "point",
                shape = 18, size = 5, color = "firebrick2")
  p
}



# C6. Forest plot of hospital attributes
plotOR_paper_hospital <- function(dataplot_CM,
                                  xl = -4, xu = 14,
                                  atx = c(-3, -2, -1, 0, 1, 2, 3),
                                  xlab_title = "Effect of covariates on mean COVID hospitalization (in days)",
                                  title = ""){

  lc <- nrow(dataplot_CM)
  revy <- seq_len(lc)

  adj_x <- dataplot_CM$Effect;
  adj_lb <- dataplot_CM$lb; adj_ub <- dataplot_CM$ub



  adj_xl <- xl
  adj_xu <- xu
  plot(x = adj_x,
```

© 2020 Asch DA et al. JAMA Internal Medicine.

```
    y = rev(seq_len(lc)),
    xlab = xlab_title,
    main = '', cex.axis = 2,
    ylab = '', xaxt = "n", lwd = 2,
    cex.lab = 1.5, cex.main = 2,
    cex = 2, yaxt = "n", pch = NA,
    xlim = c(adj_xl, adj_xu))



atx <- atx
axis(1, at = atx, labels = atx, cex.axis = 1.7, lwd = 1)

ven_tex <- nrow(dataplot_CM) + 1.5

RR <- as.character(dataplot_CM$reference)
dataplot_CM$reference <- RR

# Var names
mtext(side = 2, dataplot_CM$ylab_name,
      at = rev(revy),
      line = 5, las = 2, cex = 0.8,
      font = 1,
      col = "black")

# Count
mtext(side = 2, as.vector(dataplot_CM$ylab_name_numb[,1]),
      at = rev(revy),
      line = 17, las = 2, cex = 0.8,
      font = 1,
      col = "black")
```

© 2020 Asch DA et al. JAMA Internal Medicine.

```
# Reference names
mtext(side = 2, StrAlign(dataplot_CM$reference, sep = "\\l"),
      at = rev(revy),
      las = 1, cex = 0.8, line = 0, font = 1, col = "black")

# CI values
mtext(side = 4, StrAlign(dataplot_CM$allpoint, sep = "\\r"), at = rev(revy), las = 2,
      cex = 0.8, font = 1, col = "black")

col_pv <- "black"

# p-value
mtext(side = 4, StrAlign(dataplot_CM$pv, sep = "\\r"),
      at = rev(revy), las = 2,
      cex = 0.8, line = 8, font = 2,
      col = col_pv)


# Column names
mtext(side = 2, paste0("Reference"), at = (ven_tex),
      line = 0,
      las = 1, cex = 1, lwd = 1,
      font = 2, col = "black")

mtext(side = 2, paste0("Characteristic"), at = (ven_tex),
      line = 7,
      las = 1, cex = 1, lwd = 1, font = 2,
      col = "black")
```

© 2020 Asch DA et al. JAMA Internal Medicine.

```
mtext(side = 2, paste0("Size"), at = (ven_tex),
    line = 17,
    las = 1, cex = 1, lwd = 1, font = 2,
    col = "black")

mtext(side = 4, paste0("Estimate (95% CI)"),
    at = ven_tex,
    line = -1,
    las = 1, cex = 1,
    lwd = 1, font = 2,
    col = "black")

mtext(side = 4, paste0("p-value"),
    at = ven_tex,
    line = 8,
    las = 1, cex = 1,
    lwd = 1, font = 2,
    col = "black")

segments(x0 = xl, x1 = xu, y0 = revy, y1 = revy,
      col = "#EEEEEE", lty = 1, lwd = 2)

segments(round(adj_lb, 2), rev(revy),
      round(adj_ub, 2), rev(revy),
      col = "black", lty = 1, lwd = 2)

segments(x0 = 0, x1 = 0,
      y0 = 0, y1 = tail(revy, 1) + 1,
      col = "black", lty = 2,
      lwd = 2)
```

© 2020 Asch DA et al. JAMA Internal Medicine.

```
  points(adj_x, rev(seq_len(lc)),
         xlab = '', lwd = 2,
         col = "black", cex = 1.5, pch = 3)
}


# C7. Descriptive comparisons
quintile_descriptive <- function(Data_score, id_q){

  dataqa <- data.frame("score" = datam$score,
                    "group" = id_q,
                    "reference" = datam$reference)
  description   <- describeBy(dataqa$score, dataqa$group, digits = 4, mat = TRUE)
  a1 <- round(description[, 5] - 1.96 * description[, 6] / sqrt(description[, 4]), 4)
  a2 <- round(description[, 5] + 1.96 * description[, 6] / sqrt(description[, 4]), 4)


  ## difference from Q5
  lb_dif <- (description$mean[5] - description$mean) - 1.96 *
    sqrt((description$sd^2)/description$n +
         (description$sd[5]^2)/description$n[5])

  mean_dif <- (description$mean[5] - description$mean)

  ub_dif <- (description$mean[5] - description$mean) + 1.96 *
    sqrt((description$sd^2)/description$n +
         (description$sd[5]^2)/description$n[5])

  z_dif <- mean_dif / sqrt((description$sd^2)/description$n + (description$sd[5]^2)/description$n[5])
  p_value_dif <- 2 * (1 - pnorm(abs(z_dif)))
```

© 2020 Asch DA et al. JAMA Internal Medicine.

```
  mean_dif[5] <- ub_dif[5] <- lb_dif[5] <- z_dif[5] <- p_value_dif[5] <- NA

  a3 <- c(colnames(description), "lb_mean", "ub_mean",
        "mean_dif_Q5", "lb_dif_Q5", "ub_dif_Q5", "z_dif", "p_value_dif")
  description <- cbind(description, a1, a2, mean_dif, lb_dif, ub_dif, z_dif, p_value_dif)
  colnames(description) <- a3
  rownames(description) <- c("Q1", "Q2", "Q3", "Q4", "Q5")

  output = list(description = description)
}
```

## Simulating patient outcome data

Here we simulate patient outcome data for different hospitals. Note that data is generated without considering any clinical perspective and thus does not mean anything intuitively. This is solely for the purpose of illustrating the adopted methodologies to compute risk standardized scores. Consider response being a binary variable with 1 denoting patient-specific adverse event and 0 otherwise. The covariates consist of simulated demographic, clinical, and comorbid variables.

```{r simul}
## Simulating data
names <- c("age_class", "gender", "snf_nursing_flag",
        "aeci_aids_flag",  "aeci_alcohol_flag", "aeci_anemdef_flag",
        "aeci_arth_flag", "aeci_bldloss_flag", "aeci_chf_flag", "aeci_chrnlung_flag",
        "aeci_coag_flag", "aeci_depress_flag", "aeci_dm_flag", "aeci_dmcx_flag",
        "aeci_drug_flag", "aeci_htn_flag", "aeci_hypothy_flag", "aeci_liver_flag",
        "aeci_lymph_flag", "aeci_lytes_flag", "aeci_mets_flag", "aeci_neuro_flag",
        "aeci_obese_flag", "aeci_para_flag", "aeci_perivasc_flag", "aeci_psych_flag",
        "aeci_pulmcirc_flag", "aeci_renlfail_flag", "aeci_tumor_flag", "aeci_ulcer_flag",
        "aeci_valve_flag", "aeci_wghtloss_flag",  "count_day_class", "size",
        "mbr_death_flag", "hosp_name")
```

© 2020 Asch DA et al. JAMA Internal Medicine.

```
 # comorbidity names
 tow <- names[names %!in% c('gender', 'snf_nursing_flag', 'size', 'mbr_death_flag', 'hosp_name',
             'count_day_class', 'age_class')]

K <- 500 # Number of sites
dataN <- list()

set.seed(33)
beta_com <- round(rnorm(length(tow), 0, 1), 2)
beta_int <- rnorm(1, -2, 4)
beta_age <- rnorm(1, 1, 1)
beta_nurse <- rnorm(1, -0.5, 0.50)
beta_count <- rnorm(1, 0.10, 0.50)
beta_gender <- rnorm(1, -0.50, 0.50)
beta_size <- 0 #rnorm(1, 0.1, 0.2)
#mbr_death_flag <- list()
c(beta_com, beta_age, beta_count,  beta_nurse, beta_size, beta_int, beta_gender)
dataN_All <- do.call(rbind, lapply(seq_len(K), function(kk) {

  set.seed(7777 + kk)
  nh <- round(runif(1, 10, 200))  # number of patients in each hospital; minimum 10 and maximum 500
  dataN$hosp_name <- rep(paste0("Hospital_", kk,""), nh)
  dataN$size <- rep(nh, nh)
  dataN$size_scale <- log(dataN$size)

  for(jj in 1 : length(names)){
    if(names[jj] == "gender"){
      set.seed(jj + 8000)
      QQ <- paste0("dataN$", names[jj], " <- rbinom(", nh, ", 1, 0.60)")
      QQ <- eval(parse(text = QQ))
    }
```

© 2020 Asch DA et al. JAMA Internal Medicine.

```
    if(names[jj] == "snf_nursing_flag"){
      set.seed(jj + 1)
      QQ <- paste0("dataN$", names[jj], " <- rbinom(", nh, ", 1, 0.40)")
      QQ <- eval(parse(text = QQ))
    }
    if(names[jj] %!in% c("gender", "snf_nursing_flag", "mbr_death_flag", "hosp_name",
                         "size", "count_day_class", "age_class")){
      set.seed(jj + 1000)
      prob_c <- runif(1, 0.3, 0.7)
      QQ <- paste0("dataN$", names[jj], " <- rbinom(", nh, ", 1, prob_c)")
      QQ <- eval(parse(text = QQ))
    }
    if(names[jj] == "age_class"){
      set.seed(jj + 3000)
      dataN$age <- round(rnorm(nh, mean = 65, sd = 20)) # mean age 65 and sd 15
      dataN$age_scale <- (dataN$age - min(dataN$age)) / (max(dataN$age) - min(dataN$age))
    }
    if(names[jj] == "count_day_class"){
      set.seed(jj + 5000)
      dataN$count_day <- round(runif(nh, min = 10, max = 200)) # minimum count of day 10 and maximum 180
      dataN$count_day_scale <- (dataN$count_day - min(dataN$count_day)) / (max(dataN$count_day) -
min(dataN$count_day))
    }
  } # loop for each variable ends
  #as.data.frame(dataN)


  tow <- names[names %!in% c('gender', 'snf_nursing_flag', 'size', 'mbr_death_flag', 'hosp_name',
                             'count_day_class', 'age_class')]


  set.seed(kk + 88)
  bi <- rnorm(1, 0, 1) # 3 site-specific
```

© 2020 Asch DA et al. JAMA Internal Medicine.

```
  eij <- rnorm(nh, 0, 1.5) # each patient-specific



  EE <- paste0("dataN$", tow, " * ", beta_com, "", collapse = "+")
  QQ <- paste0("link_fix <- beta_int + dataN$count_day_scale * beta_count +
               dataN$age_scale * beta_age +
               dataN$snf_nursing_flag * beta_nurse + dataN$gender * beta_gender +
               dataN$size_scale * beta_size + ", EE, " + bi + eij")
  QQ <- eval(parse(text = QQ))
  QQp <- (exp(QQ)/(1 + exp(QQ)))
  QQp
  dataN$mbr_death_flag <- ifelse(QQp  >= 0.5, 1, 0)
  table(dataN$mbr_death_flag)
  as.data.frame(dataN)
} # loop for each hospital ends
))

for(jj in 1 : length(names)){
  if(names[jj] == "gender"){
    QQ <- paste0("dataN_All$", names[jj], " <- ifelse(dataN_All$", names[jj], " == 1,
              'Male', 'Female')")
    QQ <- eval(parse(text = QQ))
  }
  if(names[jj] == "snf_nursing_flag"){
    QQ <- paste0("dataN_All$", names[jj], " <- ifelse(dataN_All$", names[jj], " == 1,
              'Yes', 'No')")
    QQ <- eval(parse(text = QQ))
  }
  if(names[jj] %!in% c("gender", "snf_nursing_flag", "mbr_death_flag", "hosp_name",
                   "size", "count_day_class", "age_class")){
    QQ <- paste0("dataN_All$", names[jj], " <- ifelse(dataN_All$", names[jj], " == 1,
```

© 2020 Asch DA et al. JAMA Internal Medicine.

```
            'Yes', 'No')")
    QQ <- eval(parse(text = QQ))
  }
  if(names[jj] == "mbr_death_flag"){
    QQ <- paste0("dataN_All$", names[jj], " <- ifelse(dataN_All$", names[jj], " == 1,
            'Event', 'Alive')")
    QQ <- eval(parse(text = QQ))
  }
  if(names[jj] == "age_class"){
    dataN_All$age_class <- ifelse(dataN_All$age <= 45, "18 < age <= 45",
     ifelse(dataN_All$age > 45 & dataN_All$age <= 55, "45 < age <= 55",
      ifelse(dataN_All$age > 55 & dataN_All$age <= 65, "55 < age <= 65",
        ifelse(dataN_All$age > 65 & dataN_All$age <= 75, "65 < age <= 75",
         ifelse(dataN_All$age > 75 & dataN_All$age <= 85, "75 < age <= 85", "85 < age")))))
  }
  if(names[jj] == "count_day_class"){
    dataN_All$count_day_class <- ifelse(dataN_All$count_day <= 90, "1 < Count <= 90",
      ifelse(dataN_All$count_day > 90 & dataN_All$count_day <= 120, "90 < Count <= 120",
       ifelse(dataN_All$count_day > 120 & dataN_All$count_day <= 150, "120 < Count <= 150",
                                "150 < Count")))
  }
}


# defining reference categories
dataN_All <- dataN_All %>% mutate_if(is.character, as.factor)
dataN_All <- within(dataN_All, age_class <- relevel(age_class, ref = "18 < age <= 45"))
dataN_All <- within(dataN_All, gender <- relevel(gender, ref = "Female"))
dataN_All <- within(dataN_All, count_day_class <- relevel(count_day_class, ref = "150 < Count"))
dataN_All <- within(dataN_All, snf_nursing_flag  <- relevel(snf_nursing_flag, ref = "No"))
dataN_All <- within(dataN_All, mbr_death_flag <- relevel(mbr_death_flag, ref = "Alive"))
```

© 2020 Asch DA et al. JAMA Internal Medicine.

```
dataN_All$size <- log(dataN_All$size)  # log transformation

comorb <- names[names %!in% c("gender", "snf_nursing_flag", "mbr_death_flag", "size", "hosp_name",
"count_day_class", "age_class")]

for(jj in 1 : length(comorb)){

  QQ <- paste0("dataN_All <- within(dataN_All, ", comorb, " <- relevel(",comorb, ", ref = 'No'))", collapse = "; ")

  QQ <- eval(parse(text = QQ))

}


dim(dataN_All)

table(dataN_All$mbr_death_flag)

length(unique(dataN_All$hosp_name))
```

## Fitting generalized linear mixed model

Following the procedures described in Drye et al. and Silber et al., we compute hospital specific risk standardized rates (RSERs) or risk standardized mortality rates (RSMRs). In this example, we focus only on composite outcomes (i.e., RSERs).

```{r}
system.time(fit0 <- glmmfit(ylist = "mbr_death_flag",

            XXX_adj = c(names[names %!in% c("mbr_death_flag", "hosp_name")]),

            ranlist = "(1 | hosp_name)",

            reml = TRUE,

            Dataf = dataN_All,

            addsize = TRUE,

            laplace = FALSE))
```

We extract the model outputs.

```{r}
# Model outputs
```

© 2020 Asch DA et al. JAMA Internal Medicine.

```
fom <- fit0$fom; fom3 <- fit0$fom3; LRT_re <- fit0$LRT_re; aic_null <- fit0$aic_null;
aic_alt <- fit0$aic_alt; SS <- fit0$SS; cor_lme_lmer <- fit0$cor_lme_lmer;
probs <- fit0$probs; rank_cor <- fit0$rank_cor; nY <- fit0$nY; lb <- fit0$lb
ub <- fit0$ub; sdorp <- fit0$sdorp; or <- fit0$or; orp <- fit0$orp; pred <- fit0$pred;
estm <- fit0$estm; ybar <- fit0$ybar; uid <- fit0$uid;
score <- fit0$score; score_DS_mean <- apply(fit0$score_DS, 2, mean)
unadj_score <- fit0$unadj_score; hosp_orig_vol = fit0$hosp_orig_vol

# Score table
datScore_orig <- data.frame("score" = as.numeric(score),
                     "score_DS_mean" = as.numeric(score_DS_mean),
                     "reference" = as.character(uid),
                     "volume" = hosp_orig_vol,
                     "unadjusted_score" = unadj_score)

# Odds ratio table
dat <- data.frame("Variable" = names(or),
           "bOR" =  or,
           "lb" =  lb,
           "ub" = ub,
           "pval" = 2 * (1 - pnorm(abs(orp/sdorp))))

# Rearranging data for forest plot
symbol <- "\u2265"; symbol2 <- intToUtf8(8804)
ylist <- "mbr_death_flag"
mlist_age <- c("age_class"); varlist_age <- c("Age::[45,55)", "Age::[55,65)", "Age::[65,75)",
            "Age::[75,85)", paste0("Age::", symbol,"85"));
reflist_age <- rep("[18,45)", length(varlist_age)); mlist0_age <- rep("age_class",
                                     length(varlist_age))
comorb_nm <- c("Acquired immune deficiency syndrome", "alcohol abuse",
          "Iron deficiency anemia", "Rheumatoid arthritis", "bloodloss anemia",
```

© 2020 Asch DA et al. JAMA Internal Medicine.

```
        "Congestive heart failure", "Chronic obstructive pulmonary disease",
        "Coagulopathy", "depression", "Diabetes without chronic complication",
        "Diabetes with chronic complication", "drug abuse", "hypertension",
        "hypothyroidism", "liver disease", "lymphoma", "fluid/electrolyte disorder",
        "metastatic cancer", "neurological disorders", "obesity", "paralysis",
        "peripheral vascular disease" ,"psychosis", "pulmonary circulation disorder",
        "Chronic kidney disease", "tumor w/o metastasis", "peptic ulcer disease",
        "valvular disorder", "Weight loss")

mlist_gender <- c("gender"); varlist_gender <- c("Gender::Male"); reflist_gender <- rep("Female",
length(varlist_gender)); mlist0_gender <- rep("gender", length(varlist_gender))
mlist_comorbidity <- c(tow); varlist_comorbidity <- paste0("", comorb_nm, "::Yes", sep = "");
reflist_comorbidity <- rep("No", length(varlist_comorbidity)); mlist0_comorbidity <- tow
mlist_nurse <- c("snf_nursing_flag"); varlist_nurse <- c("Transferred from nursing facility::Yes");
reflist_nurse <- rep("No", length(varlist_nurse)); mlist0_nurse <- rep("snf_nursing_flag",
                                                   length(varlist_nurse))
mlist_count <- c("count_day_class"); varlist_count <- c("Days from January 1,2020::[0,90)",
          "Days from January 1,2020::[90,120)", "Days from January 1,2020::[120,150)")
reflist_count <- rep(paste0("", symbol,"150"), length(varlist_count));
mlist0_count <- rep("count_day_class", length(varlist_count))
mlist_size <- c("size"); varlist_size <- c("Log(volume)"); reflist_size <- NA;
mlist0_size <- c("size")

## Fixed effects
variable_list = "Age:Gender:Nurse:Comorbidity:Count:Size"
txt <- strsplit(variable_list, ":")[[1]]
txt2 <- paste0("mlist_", tolower(txt), "", sep = "", collapse = ", ")
XXX <- paste0("c(", txt2,")")
XXX <- eval(parse(text = XXX))
variable_list_BAR <- paste0("", txt, collapse = "|")
```

© 2020 Asch DA et al. JAMA Internal Medicine.

```
txt <- strsplit(variable_list, ":")[[1]]
## final name
txt2 <- paste0("mlist0_", tolower(txt), "", sep = "", collapse = ", ")
QQ <- paste0("c(", txt2,")")
QQ <-  eval(parse(text = QQ))

inout_ix <- unlist(lapply(seq_len(length(QQ)), function(ii) {
  inout <- grep(QQ[ii], dat$Variable, value = F)
  if(length(inout) >= 1){
    1
  } else{
    9999 # missing
  }
}))

lni <- which(inout_ix == 9999)
if(length(lni) > 0){
  dat$finalname <-  QQ[- which(inout_ix == 9999)]
} else{
  dat$finalname <- QQ
}

txt2 <- paste0("varlist_", tolower(txt), "", sep = "", collapse = ", ")
QQ <- paste0("c(", txt2,")")
QQ <- eval(parse(text = QQ))

if(length(lni) > 0){
  dat$ylab_name <-  QQ[- which(inout_ix == 9999)]
} else{
  dat$ylab_name <- QQ
}
```

© 2020 Asch DA et al. JAMA Internal Medicine.

```
txt2 <- paste0("reflist_", tolower(txt), "", sep = "", collapse = ", ")
QQ <- paste0("c(", txt2,")")
QQ <- eval(parse(text = QQ))

if(length(lni) > 0){
  dat$reference <- QQ[- which(inout_ix == 9999)]
} else{
  dat$reference <- QQ
}
name_coef <- as.character(dat$Variable)

ylab_name_numb <- t(do.call(cbind, lapply(seq_len(nrow(dat)), function(uu) {
  where <- dat$finalname[uu]
  numb <- table(dataN_All[, colnames(dataN_All) %in% where])
  what <- strsplit(name_coef[uu], where)[[1]][2]
  numb <- numb[which(names(numb) == what)]
  QQ <- subset(dataN_All, select = c(where, ylist))
  head(QQ)
  numb_event <- length(which(QQ[, 1] == what & QQ[, 2] == "Event"))
  as.vector(c(numb, numb_event))
})))
dat$ylab_name_numb <- ylab_name_numb[, 1]
dat$ylab_name_numb_event <- ylab_name_numb[, 2]
dat$allpoint <- unlist(lapply(seq_len(nrow(dat)), function(hh)
  paste0("",sprintf("%2.2f",round(dat$bOR[hh], 2)),"(",sprintf("%2.2f",round(dat$lb[hh],
2)),",",sprintf("%2.2f",round(dat$ub[hh], 2)), ")")))
dat$ylab_name_numbX = paste0("", dat$ylab_name_numb_event, "/", dat$ylab_name_numb, "")
zerome <- which(dat$ylab_name_numbX == "0/0")
if(length(zerome) > 0){
  dat$ylab_name_numbX[zerome] <- NA
```

© 2020 Asch DA et al. JAMA Internal Medicine.

```
}
```

## Comparison between two methods

```{r}
sm1 <- summary(datScore_orig$score)   # Dyre et al.
sm2 <- summary(datScore_orig$score_DS_mean) # Silber et al.

cr <- cor(datScore_orig$score, datScore_orig$score_DS_mean)
par(bty = "n", mar = c(5, 5, 5, 5), mgp = c(2, 0.5, 0), xpd = FALSE, tcl = 0.25, cex = 0.9)
plot(x = datScore_orig$score,
    y = datScore_orig$score_DS_mean,
    xlim = c(0, 0.3), ylim = c(0, 0.3),
    xlab = "Dyre et al. (P/E * y_bar)",
    ylab = "Silber et al.",
    cex.axis = 2,
    pch = 19,
    col = adjustcolor("blue", alpha.f = 0.20),
    type = "p",
    cex = 1,
    cex.lab = 1.5)
abline(0, 1, lwd = 1)
text(0.15, 0.02, cex = 1.5, paste0("Correlation between scores = ", round(cr, 4),""))

# variable
legend(x = 0.03, y = 0.35, ncol = 1L, bty = "n",
      cex = 1.5,
      x.intersp = 2.2,
      y.intersp = 1,
      inset = c(0.01, 0),
      text.width = c(strwidth("1,000,00")),
```

© 2020 Asch DA et al. JAMA Internal Medicine.

```
       xjust = 1, yjust = 1,
       title = '',
       legend = c("", c("Minimum", "Q1",
                        "Median", "Mean",
                        "Q3", "Maximum")))

legend(x = 0.09, y = 0.35, ncol = 1L, bty = "n",
       cex = 1.5,
       x.intersp = 2.2,
       y.intersp = 1,
       inset = c(0.01, 0),
       text.width = c(strwidth("1,000,00")),
       xjust = 1, yjust = 1,
       title = '',
       legend = c('Dyre',
                  sprintf("%6.4f", c(round(sm1, 4)))))

legend(x = 0.14, y = 0.35, ncol = 1L, bty = "n",
       cex = 1.5,
       #x.intersp = 1.8,
       #y.intersp = 1,
       x.intersp = 2.2,
       y.intersp = 1,
       inset = c(0.01, 0),
       text.width = c(strwidth("1,000,00")),
       xjust = 1, yjust = 1,
       title = '',
       legend = c('Silber',
                  sprintf("%6.4f", c(round(sm2, 4)))))

ˋˋˋ
```

© 2020 Asch DA et al. JAMA Internal Medicine.

````
## Model performance evaluation

```{r}

validation <- data.frame("Test of random-effects" = sprintf("%0.4f", as.numeric( fit0$LRT_re)),

                    "AIC of null model (GLM)" = sprintf("%0.4f", as.numeric(fit0$aic_null)),

                    "AIC of full model (GLMM)" = sprintf("%0.4f", as.numeric(fit0$aic_alt)),

                    "Correlation of estimates" = sprintf("%0.4f", as.numeric(fit0$cor_lme_lmer$estimate)),

                    "Test of correlation" = sprintf("%0.4f", as.numeric(fit0$cor_lme_lmer$p.value)),

                    "C-statistic (random prediction)" = sprintf("%0.4f", as.numeric(fit0$rank_cor[1])),

                    "Dxy (rank-correlation)" = sprintf("%0.4f", as.numeric(fit0$rank_cor[2])),

                    "Marginal R-square" = sprintf("%0.4f", as.numeric(fit0$SS[2])),

                    "Conditional R-square" = sprintf("%0.4f", as.numeric(fit0$SS[1])))

validation

```


## Estimation of adjusted odds ratio

```{r}

dat$pval <- sprintf("%0.4f", dat$pval)

dat$pval[which(dat$pval == "0.0000")] <- "<0.0001"

par(bty = "n", mar = c(2, 25, 1, 7), mgp = c(2, 0.5, 0), xpd = FALSE, tcl = 0.25, cex = 0.9)


plotOR_paper(dataplot = dat,

          xl = 0.5, xu = 15,

          title = "",

          txtcol = paste0("No. Events/\nCOVID patients"), xlab_title =

  paste0("Estimated odds ratio of death or transfer-to-hospice within 30 days post-admission"))

```


## Hospital specific risk standardized event rates

```{r}

ORDER_FF <- datScore_orig[order(datScore_orig$score_DS_mean), ]
````

© 2020 Asch DA et al. JAMA Internal Medicine.
````

```
dataplotR <- data.frame("score" = as.numeric(ORDER_FF$score_DS_mean),
            "reference" = ORDER_FF$reference,
            "volume" = ORDER_FF$volume,
            "unadj_score" = ORDER_FF$unadjusted_score)

plotScore_paper_single_rotate(dataplot = dataplotR,
              dataplot2 = NULL,
              xl = 0,
              xu = 1,
              maxX = 500,
              sec_xl = -0.15,
              sec_xu = 0.75,
              atx1 = c(1, 100, 200, 300, 400, 500),
              type = NULL,
              plot_unadj = FALSE,
              ybar_early = NULL,
              ybar_later = NULL,
              ybar_orig = fit0$ybar,
              confidence = FALSE,
              title = "Hospital-specific standardized risk score",
              ylab_title = "Standardized score",
              xlab_title = "Hospital rank",
              legend_title = "event")
```

© 2020 Asch DA et al. JAMA Internal Medicine.
